# Cryptic taxonomic diversity and high-latitude melanism in the glossiphoniid leech assemblage from the Eurasian Arctic

**DOI:** 10.1038/s41598-022-24989-7

**Published:** 2022-11-30

**Authors:** Ivan N. Bolotov, Alexander V. Kondakov, Tatyana A. Eliseeva, Olga V. Aksenova, Evgeny S. Babushkin, Yulia V. Bespalaya, Elena S. Chertoprud, Gennady A. Dvoryankin, Mikhail Yu. Gofarov, Anna L. Klass, Ekaterina S. Konopleva, Alexander V. Kropotin, Artem A. Lyubas, Alexander A. Makhrov, Dmitry M. Palatov, Alexander R. Shevchenko, Svetlana E. Sokolova, Vitaly M. Spitsyn, Alena A. Tomilova, Ilya V. Vikhrev, Natalia A. Zubrii, Maxim V. Vinarski

**Affiliations:** 1 N. Laverov Federal Center for Integrated Arctic Research of the Ural Branch of the Russian Academy of Sciences, Northern Dvina Emb. 23, 163069 Arkhangelsk, Russia; 2 SSC/IUCN-Mollusc Specialist Group, Species Survival Commission, International Union for Conservation of Nature, Cambridge, CB2 3QZ UK; 3 Northern Arctic Federal University, Northern Dvina Emb. 17, 163002 Arkhangelsk, Russia; 4 Laboratory of Macroecology and Biogeography of Invertebrates, Saint Petersburg State University, 7/9 Universitetskaya Emb., 199034 Saint Petersburg, Russia; 5 Surgut State University, Lenina Ave., 1, 628403 Surgut, Russia; 6 Tyumen Scientific Center, Siberian Branch of the Russian Academy of Sciences, Malygina St., 86, 625026 Tyumen, Russia; 7 A. N. Severtsov Institute of Ecology and Evolution of the Russian Academy of Sciences, Leninsky Prt., 33, 119071 Moscow, Russia; 8 Department of General Ecology and Hydrobiology, M. V. Lomonosov Moscow State University, 119234 Moscow, Russia; 9 Institute of Molecular Genetics of the National Research Centre “Kurchatov Institute”, Kurchatov Square 2, 123182 Moscow, Russia

**Keywords:** Taxonomy, Biogeography, Biodiversity

## Abstract

The family Glossiphoniidae is a diverse and widespread clade of freshwater leeches, playing a significant role in functioning of aquatic ecosystems. The taxonomy and biogeography of leeches from temperate, subtropical, and tropical regions attracted much attention of zoologists, while their taxonomic richness and distribution in the Arctic are poorly understood. Here, we present an overview of the Eurasian Arctic Glossiphoniidae based on the most comprehensive occurrence and DNA sequence datasets sampled to date. This fauna contains 14 species, belonging to five genera and three subfamilies. One genus and five species are new to science and described here. The world’s northernmost occurrences of glossiphoniids are situated on the Taymyr Peninsula at 72° N, although further records at higher latitudes are expected. Most Arctic leeches are characterized by broad ranges crossing several climatic zones (e.g., *Glossiphonia balcanica* and *G. nebulosa*), although the distribution of two new species may be confined to the high-latitude areas. The Taymyr Peninsula with the nearby Putorana Plateau represents the most species-rich area (totally 9 species), while the European Arctic, Iceland, Kolyma Highland, and Chukotka Peninsula house depleted faunas (2–4 species per subregion). Finally, we show that the high-latitude melanism is a common phenomenon in glossiphoniid leeches.

## Introduction

Freshwater leeches form a widespread ecological group, which contains members of several families of the subclass Hirudinea such as the Glossiphoniidae, Erpobdellidae, Piscicolidae, and others^1^. The family Glossiphoniidae Vaillant, 1890 represents an entirely freshwater clade^2,3^. Species-rich faunas of the glossiphoniid leeches are described from tropical and subtropical regions of South America^4–6^, Asia^7–9^, and Africa^2^, as well as from temperate Europe and North America^2,10^. These areas have historically attracted much attention of researchers.

In contrast, the large body of regional research and a few available global reviews on the taxonomy and biogeography of freshwater leeches barely contain references to the Arctic fauna^1,2,11^. A comprehensive overview of the leech’s adaptations to extreme environments considers only marine piscicolids as common inhabitants of polar oceans, tolerant to very low temperatures^12^. Blanchard^13^ published the first article on freshwater Hirudinea from the Arctic. This author examined a few specimens collected from the northern edge of Scandinavia and described two new species, which were placed in the genus *Placobdella* Blanchard, 1893^13^. Wiedemann^14^ authored a brief note on freshwater and marine leeches from the Arctic using the scarce information available to date, while Lukin^15^ considered the Eurasian Arctic as a separate biogeographic subregion, housing a depleted, taxonomically poor fauna of freshwater leeches. Sawyer^2^ delineated the Boreal Subregion of the Palearctic Region, which, according to him, is characterized by a largely homogeneous freshwater leech fauna, but differs from others based on a few (sub)endemic species such as *Acanthobdella peledina* Grube, 1851 (Acanthobdellidae), *Cystobranchus mamillatus* (Malm, 1863) (Piscicolidae), and *Theromyzon maculosum* (Rathke, 1862) (Glossiphoniidae).

General taxonomic works on freshwater leech fauna of the Eurasian Arctic are virtually absent, although Blanchard’s^13^ article contains descriptions of new taxa from Arctic Scandinavia. There is a small body of literature containing faunal records and checklists of Glossiphoniidae species from different parts of this continuous region, e.g. Iceland^16,17^, Arctic Fennoscandia^13,14,18,19^, Bolshezemelskaya Tundra^20–22^, Yamal Peninsula^23,24^, Arctic Yakutia and Chukotka Peninsula^25^. It should be noted that members of other leech families such as Piscicolidae, Erpobdellidae, and Acanthobdellidae also commonly occur in the Arctic^15^, although the species richness and taxonomy of the two first groups in high-latitude areas are insufficiently known. In contrast, the family Acanthobdellidae contains only two species of primitive leeches, i.e. *Acanthobdella peledina* and *Paracanthobdella livanowi* (Epshtein, 1966), the distribution, morphological traits, and life history of which have attracted an increased attention of researchers^26–31^. Both the species were recorded from the Arctic and use salmonid fish (Salmonidae) as hosts^15,25^.

This study aims to (1) a broad-scale taxonomic reappraisal of glossiphoniid leeches from the Eurasian Arctic based on the most comprehensive dataset sampled to date; (2) description of one new genus and five new species belonging to the Arctic fauna; and (3) discussion on the species richness, cryptic diversity, distribution, and melanism patterns of the Arctic Glossiphoniidae. Here, sampling localities were considered as belonging to the Arctic, if they are situated north of the Arctic Circle (66.5636° N)^32^. Additionally, a few subarctic areas such as Iceland, the southern edge of the Malozemelskaya Tundra (Mezen town; northeast of European Russia), and the southern part of the Chukotka Peninsula were also assigned to the Arctic Region based on comparable environmental conditions.

## Results

### Taxonomic richness of the Eurasian Arctic glossiphoniid leech assemblage

**Figure 1:**
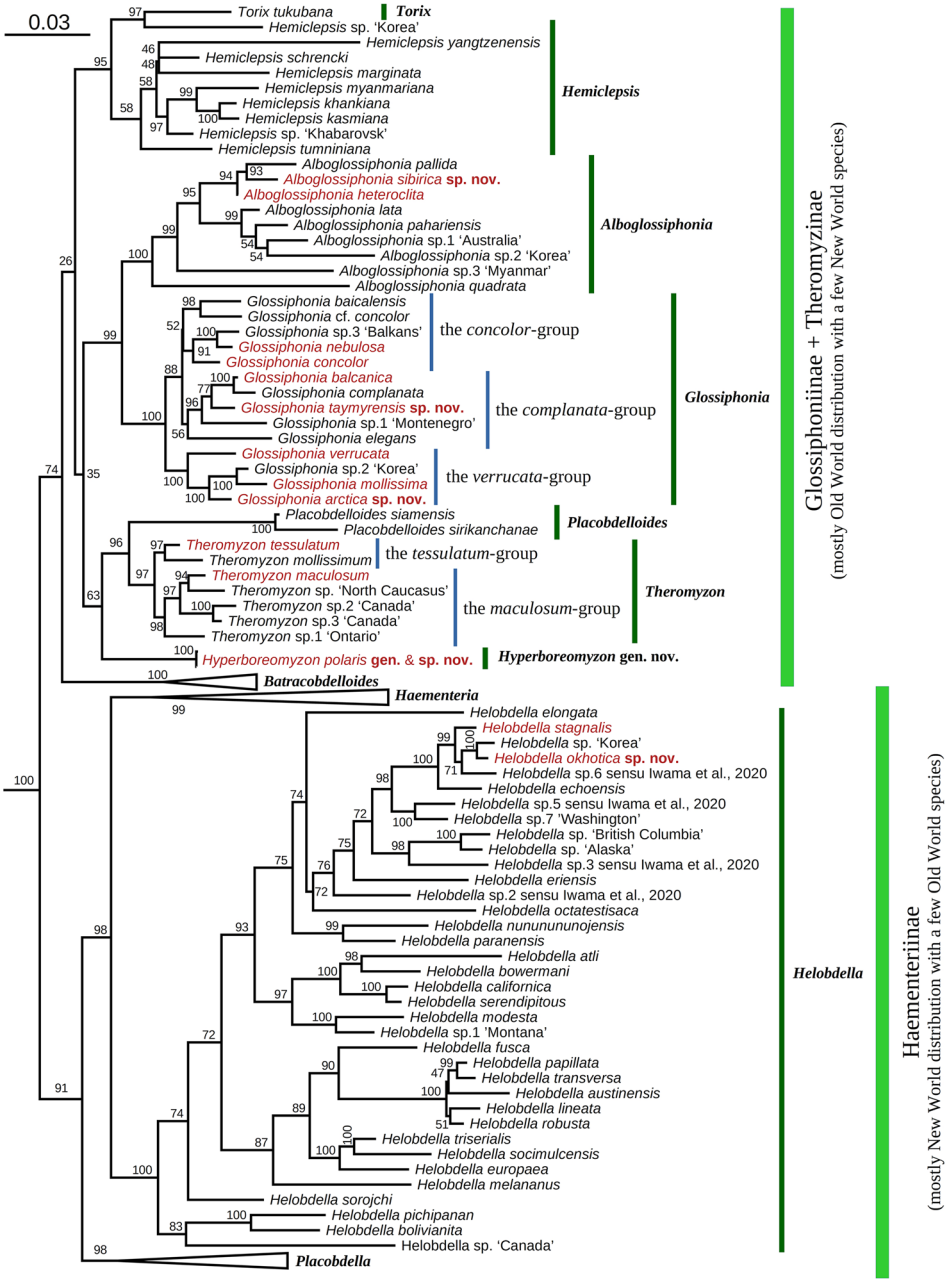
Two-locus maximum likelihood phylogeny (*COI* + *18S rRNA*) of freshwater leeches (Glossiphoniidae). Names of species presented in the Arctic fauna are red. Black numbers near nodes are bootstrap support (BS) values of IQ-TREE v. 1.6.12. Non-target genus level clades are collapsed for visualization purposes. Outgroup taxa (*Acanthobdella peledina* and nine clades represented by the Erpobdellidae, Gastrostomobdellidae, Haemadipsidae, Haemopidae, Hirudinidae, Orobdellidae, Ozobranchidae, Piscicolidae, and Salifidae) are not shown. Information on the DNA sequences is given in Supplementary Table S1.

**Figure 2:**
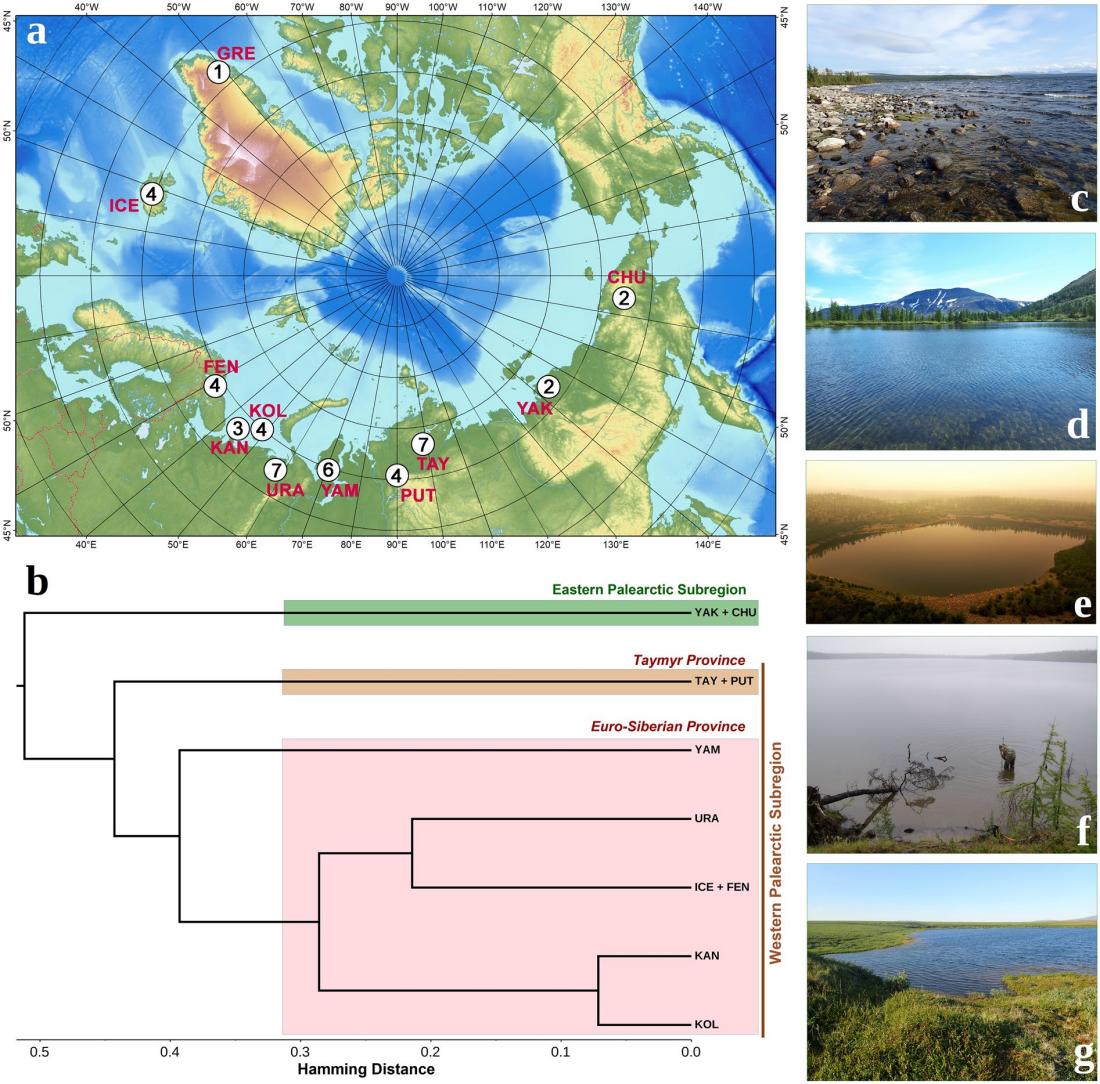
Species richness, biogeography, and habitats of freshwater leeches (Glossiphoniidae) in the Eurasian Arctic. The subregion’s codes are as follows: Iceland (ICE); Fennoscandia: Arctic Scandinavia and Kola Peninsula (FEN); Kanin Peninsula and Malozemelskaya Tundra (KAN); Kolguev Island (KOL); Bolshezemelskaya Tundra (eastern part) and Polar Urals (URA); Yamal Peninsula (YAM); Taymyr Peninsula (TAY); Putorana Plateau (PUT); Arctic Yakutia (YAK); and Chukotka Peninsula (CHU). (**a**) Map of species richness. The open circles indicate the Arctic subregions; the numbers in circles indicate the species richness of freshwater leeches in a given subregion (see Table 2 for the presence-absence data). The map was created using ESRI ArcGIS 10 software (www.esri.com/arcgis). (**b**) Dendrogram of cluster analysis (UPGMA) based on the presence-absence dataset of glossiphoniid leeches throughout subregions of the Eurasian Arctic: *pink*, Europe, the Urals, and Western Siberia; *brown*, Eastern Siberia; and *green*, Far East. The raw distribution data is presented in Table 2 and Supplementary Dataset S3. (**c**) Lake Imandra on Kola Peninsula, habitat of *Glossiphonia balcanica*. (**d**) A lake near Sob' railway station, Polar Urals, habitat of *G. arctica* sp. nov. (the type locality), *G. balcanica*, *G. concolor*, and *Helobdella stagnalis*. (**e**) An alpine lake on Putorana Plateau, the type locality of *Hyperboreomyzon polaris* gen. & sp. nov. (**f**) Olenye Lake near Khatanga on Taymyr Peninsula, the most northern locality examined by us and inhabited by the glossiphoniid leeches (72.01° N), habitat of *G. balcanica*. (**g**) A small lake near Amguema on Chukotka Peninsula, habitat of *G. mollissima*. Photos: Olga V. Aksenova (**c**,**g**); Alexander V. Kondakov (**d**); Elena S. Chertoprud (**e**); and Svetlana E. Sokolova (**f**).

**Figure 3:**
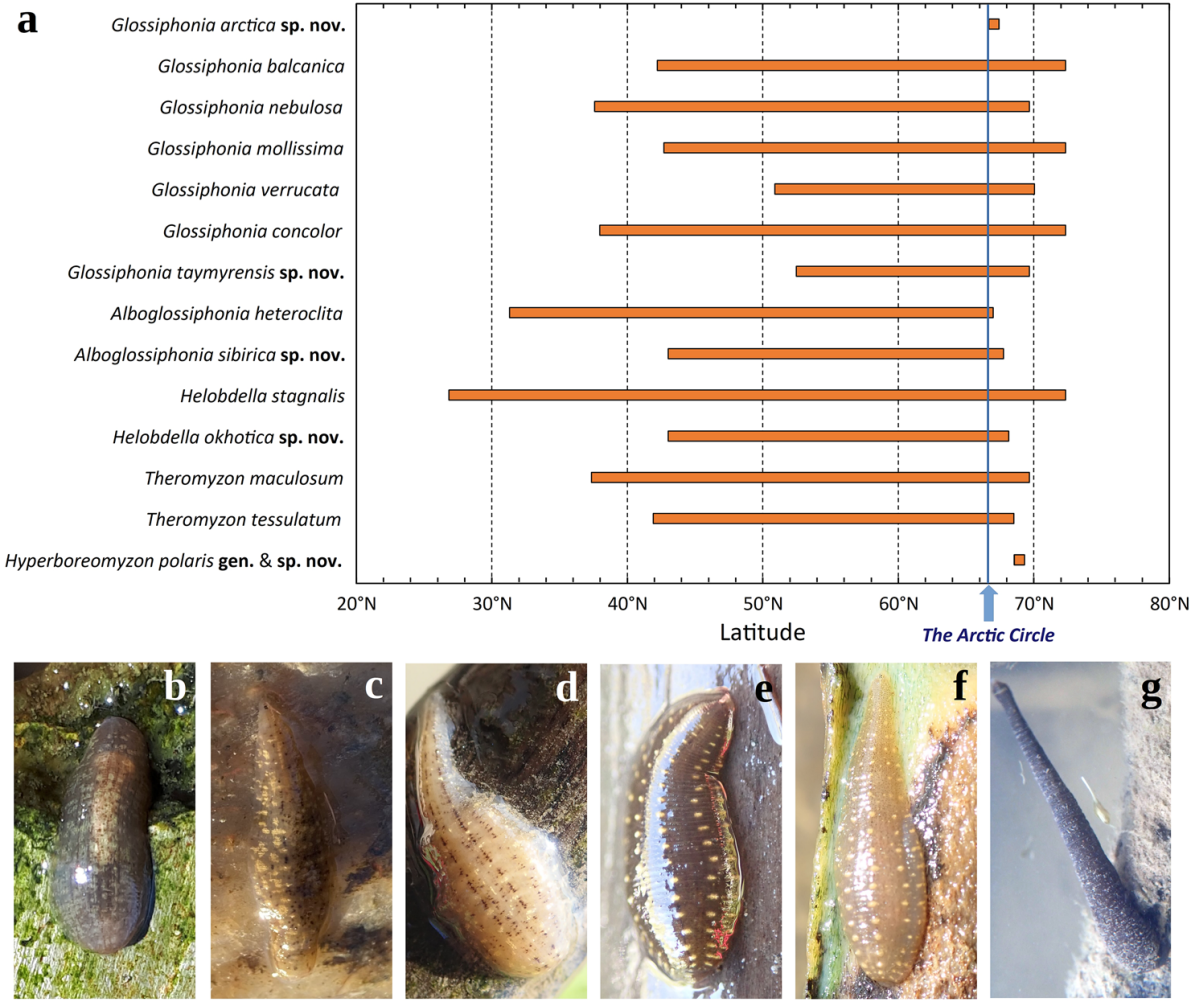
Latitudinal ranges and living individuals of the Arctic Glossiphoniidae species. (**a**) Plot of latitudinal ranges based on the extreme occurrences (northernmost and southernmost record of each species). The occurrence data with precise geographic co-ordinates is presented in Supplementary Dataset S2. (**b**) *Glossiphonia arctica* sp. nov., a lake near Sob' railway station, Polar Urals (the type locality). (**c**) *G. balcanica* (melanic form: f. ‘maculosa’), the same lake. (**d**) *G. concolor*, a small lake, Dudinka, Taymyr Peninsula. (**e**) *G. mollissima* (melanic form), Sette-Tala Lake, Chokurdakh, Arctic Yakutia. (**f**) *G. mollissima* (light-colored form), a small lake, Amguema, Chukotka Peninsula. (**g**) *Theromyzon maculosum* (melanic form), a small lake, Dudinka, Taymyr Peninsula. Photos: Alexander V. Kondakov (**b**,**c**) and Olga V. Aksenova (**d**–**g**).

**Figure 4:**
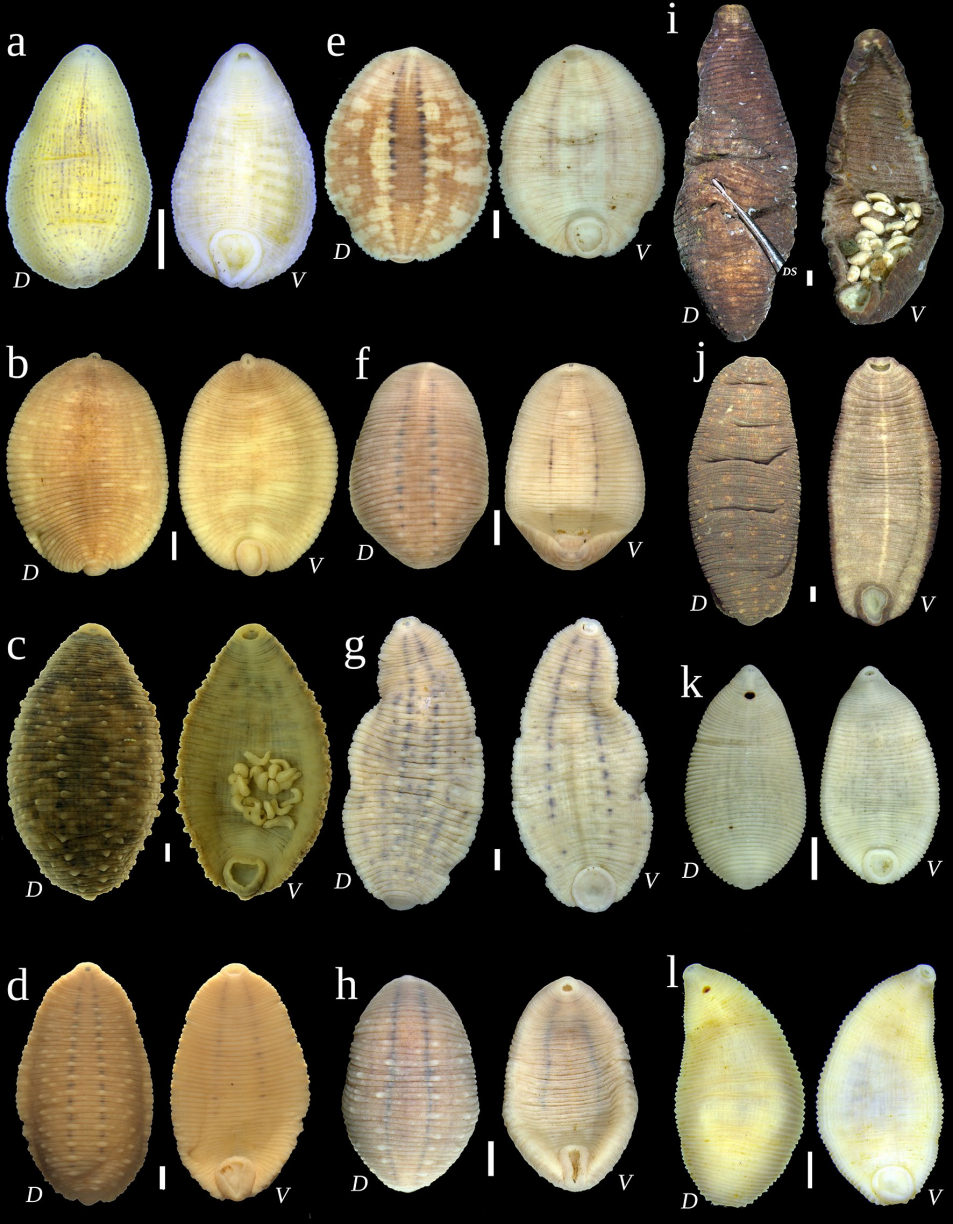
Dorsal (*D*) and ventral (*V*) views of Glossiphoniidae species under discussion. (**a**) *Alboglossiphonia sibirica* sp. nov. (holotype RMBH Hir_0542_2-H, Primorye). (**b**) *Glossiphonia arctica* sp. nov. (holotype RMBH Hir_0457_2_1-H, Polar Urals). (**c**) *G. verrucata* (melanic form; specimen RMBH Hir_0605_1, Putorana Plateau). (**d**) *G. mollissima* (light-colored form; specimen RMBH Hir_0188_2, Yakutia Republic). (**e**) *G. taymyrensis* sp. nov. (melanic form; holotype RMBH Hir_0258_1-H, Taymyr). (**f**) *G. balcanica* (light-colored form; specimen RMBH Hir_0250, Kola Peninsula). (**g**) *G. concolor* (specimen RMBH Hir_0263_2, Taymyr). (**h**) *G. nebulosa* (specimen RMBH Hir_0261_1, Taymyr). (**i**) *Theromyzon maculosum* (melanic form; specimen RMBH Hir_0263_3, Taymyr). (**j**) *Hyperboreomyzon polaris* gen. & sp. nov. (holotype RMBH Hir_0486-H, Putorana Plateau). (**k**) *Helobdella stagnalis* (specimen RMBH Hir_0269, Taymyr). (**l**) *H. okhotica* sp. nov. (holotype RMBH Hir_251_1-H, Chukotka Peninsula). Abbreviations: *DS*, dissecting needle. Scale bars = 1.0 mm. Photos: Anna L. Klass and Tatyana A. Eliseeva.

**Figure 5:**
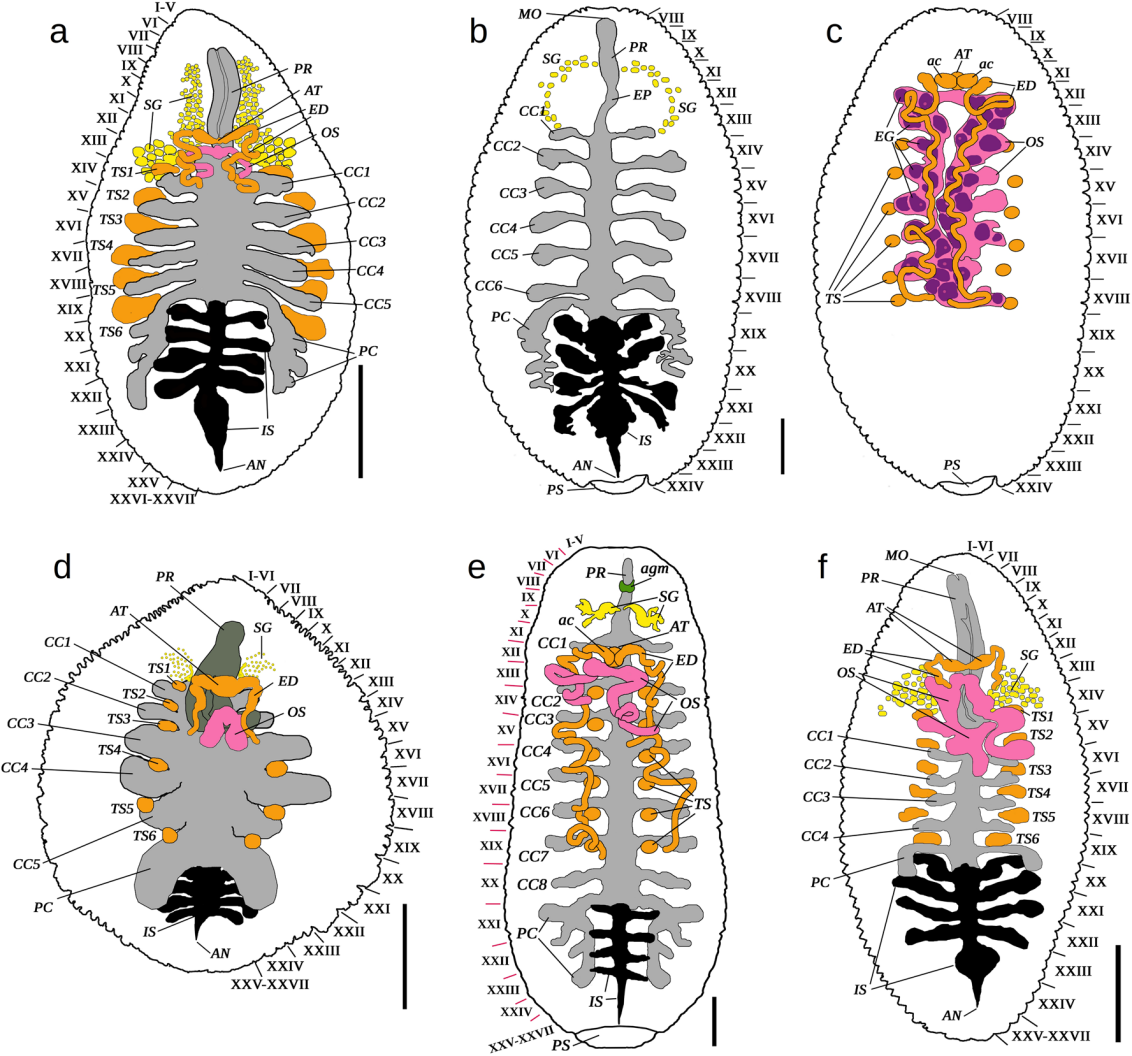
General scheme of the digestive and generative systems of the new taxa of the Glossiphoniidae (dorsal view; line contours of body and posterior sucker are shown). (**a**) *Alboglossiphonia sibirica* sp. nov. (paratype RMBH Hir_0013, Yakutia Republic). (**b**,**c**) *Glossiphonia arctica* sp. nov. (paratype RMBH Hir_0457, Polar Urals): digestive (**b**) and generative (**c**) systems. (**d**) *G. taymyrensis* sp. nov. (paratype RMBH Hir_0256_1, Taymyr Peninsula). (**e**) *Hyperboreomyzon polaris* gen. & sp. nov. (paratype RMBH Hir_0216, Kolguev Island). (**f**) *Helobdella okhotica* sp. nov. (paratype RMBH Hir_0491_1, Kamchatka Peninsula). *MO* mouth, *PR* proboscis sheath, *EP* esophagus, *SG* salivary glands, *CC* pairs of crop caeca with their numbers, *PC* the last pair of crop caeca (posterior caeca), *IS* intestine, *AN* anus, *AT* atrium, *ac* atrial cornua, *ED* ejaculatory ducts, *TS* testisacs with their numbers, *OS* ovisacs, *EG* eggs, *PS* posterior sucker, *agm* anterior ganglionic mass. Body somites are indicated by roman numerals. Scale bars = 1.0 mm (**a**–**d**,**f**) and 2.0 mm (**e**). Graphics: Ivan N. Bolotov.

**Figure 6:**
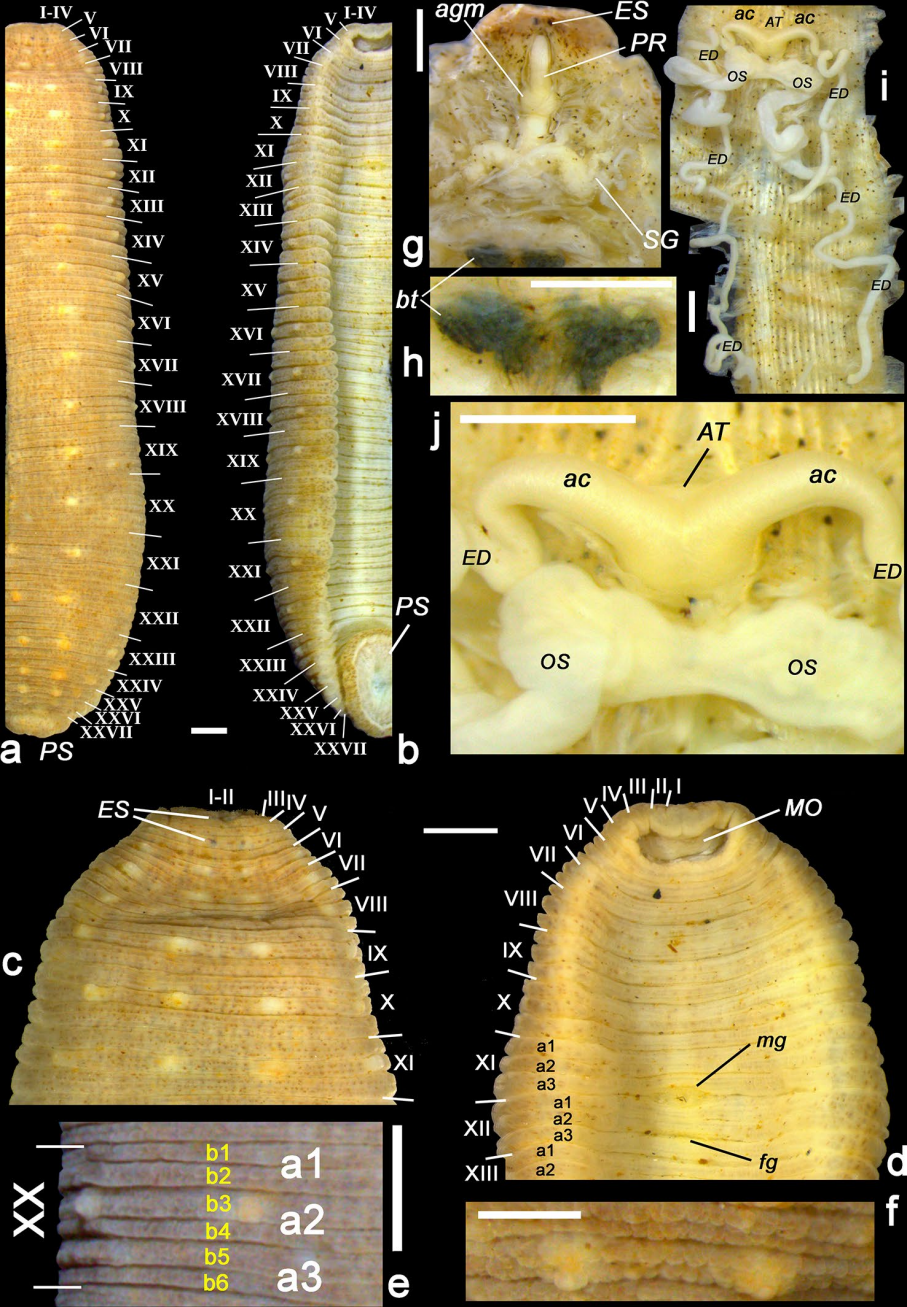
Morphological and anatomical features of *Hyperboreomyzon polaris* gen. & sp. nov. (paratype RMBH Hir_0216, Kolguev Island). (**a**,**b**) Body annulation in dorsal (**a**) and ventral (**b**) view. (**c**,**d**) Anterior region in dorsal (**c**) and ventral (**d**) view. (**e**) Example of a sexannulate mid-body somite (left half of XX) with six secondary semi-annuli (b1–b6). (**f**) Enlarged fragment of a somite, showing characteristic ‘fish scale’-like papillation (dorsal view). (**g**) Dissected anterior region (dorsal view) showing proboscis sheath, salivary glands, and the patch of black tissue of unknown function, covering the dorsal surface of the atrium. (**h**) Close up view of the black tissue patch (dorsal view). (**i**) Generative system (dorsal view). (**j**) Atrium. *ES* eyespots, *MO* mouth, *PR* proboscis sheath, *agm* anterior ganglionic mass, *bt* black tissue, *SG* salivary glands, *AT* atrium, *ac* atrial cornua, *ED* ejaculatory ducts, *OS* ovisacs, *mg* male gonopore, *fg* female gonopore, *PS* posterior sucker. Body somites are indicated by roman numerals. Scale bars = 1.0 mm (**a**–**d**,**f**–**i**) and 0.5 mm (**e**). Images of the leech body (**a**–**d**,**f**) were lightened using a Brightness/Contrast tool of Adobe Photoshop CS v. 8.0 (see Supplementary Fig. S10a for natural coloration of this specimen). Photos: Anna L. Klass; graphics: Ivan N. Bolotov.

**Figure 7:**
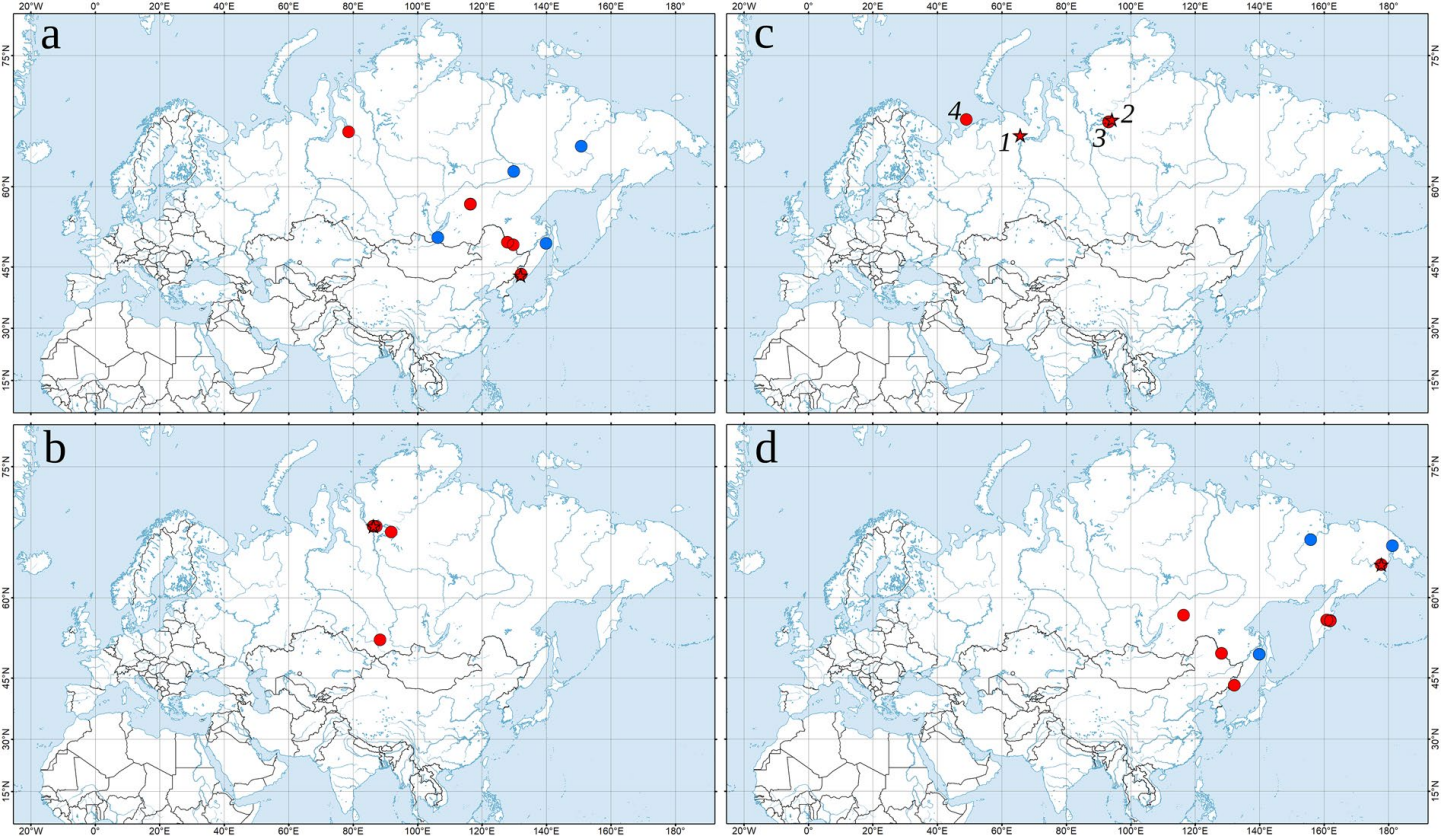
Maps of the type localities and occurrences of the new Glossiphoniidae species. (**a**) *Alboglossiphonia sibirica* sp. nov. (**b**) *Glossiphonia taymyrensis* sp. nov. (**c**) *G. arctica* sp. nov. (*1*) and *Hyperboreomyzon polaris* gen. & sp. nov. (*2–4*). (**d**) *Helobdella okhotica* sp. nov. The red stars indicate the type localities; the red circles indicate original records; the blue circles indicate published records (see Supplementary Dataset 2 for raw occurrence data and literature sources). The map was created using ESRI ArcGIS 10 software (www.esri.com/arcgis).

Here, we present the most comprehensive occurrence, morphological, and DNA-sequence datasets on the glossiphoniid leeches from the Eurasian Arctic sampled to date (Figs. 1, 2, Tables 1, 2, Supplementary Figs. S1–S13, Supplementary Table S1, and Supplementary Datasets S1–S3). The fauna of the northern margin of the continent contains 14 species belonging to five genera and three subfamilies (Figs. 3, 4, 5, 6, 7, Table 1, Supplementary Figs. S2–S13). Five species and one genus are new to science and described below. The genus *Glossiphonia* Johnson, 1816 is the most species-rich clade of freshwater leeches in the Arctic fauna. Altogether seven species in this genus are recorded north of the Arctic Circle: *G. arctica* sp. nov., *G. balcanica* Grosser & Pešić, 2016, *G. concolor* (Apáthy, 1888), *G. mollissima* Moore, 1951, *G. nebulosa* Kalbe, 1964, *G. taymyrensis* sp. nov., and *G. verrucata* (F. Müller, 1844) (Figs. 3b–f, 4b–h). In contrast, its sister genus, *Alboglossiphonia* Lukin, 1976, is poorly represented in the Arctic, with two species, *A. heteroclita* (Linnaeus, 1761) and *A. sibirica* sp. nov., occurring in the northern part of the lower Ob River basin (Fig. 4a). Two *Helobdella* species, that is, *H. stagnalis* (Linnaeus, 1758) and *H. okhotica* sp. nov., are allopatrically occur in the Eurasian Arctic, (Fig. 4k,l). Two duck leeches, *Theromyzon maculosum* (Rathke, 1862) (Figs. 3g, 4i) and *T. tessulatum* (O. F. Müller, 1773), parasitizing waterfowl, are known to occur north of the Arctic Circle. Finally, the discovery of *Hyperboreomyzon polaris* gen. & sp. nov. was completely unexpected (Fig. 4j). This taxon seems to be very rare as it was collected from only two areas, albeit rather distant from each other: Kolguev Island (Eastern Europe) and Putorana Plateau (Eastern Siberia), separated by a distance of approx. 1800 km, probably indicating a disjunctive range.

**Table 1:** Checklist of glossiphoniid leech species (Glossiphoniidae) recorded from the Eurasian Arctic (including Iceland).

Taxon	Type locality (TL)	Arctic occurrences	General range
**Subfamily Glossiphoniinae Vaillant, 1890**			
**Genus *Alboglossiphonia* Lukin, 1976**			
*A. heteroclita* (Linnaeus, 1761) = *Hirudo heteroclita* Linnaeus (1761): 506^119^	”Lacu Leufstadiensi” (Sweden: a lake near the former iron factory at Leufsta Bruk (Lövstabruk), 60.4059°N, 17.8826°E, Uppsala County)^119^	Yamal Peninsula	Europe, Western Siberia east to the Ob River basin; Kyrgyzstan^94^; Kazakhstan^15^; North Africa (Morocco and Tunisia)^96,120,121^
*A. sibirica* sp. nov.	Russia: Lake Torfyanka, 43.0761° N, 131.9620° E, Vladivostok, Primorye	Yamal Peninsula	Siberia and Russian Far East; Mongolia^39^
**Genus *Glossiphonia* Johnson, 1816**			
**The *complanata*-group**			
*G. balcanica* Grosser & Pešić, 2016 = *G. balcanica* Grosser & Pešić in Grosser et al. (2016): 18^61^	Kosovo: spring Toplla, 42.5719° N, 20.2906° E, KS40 Dečani/Decan, Lebush^61^	Kola and Kanin peninsulas, Kolguev Island, Polar Urals, Yamal and Taymyr peninsulas; Iceland	Arctic Region from Northern Fennoscandia to Taymyr; Iceland^16^; European Russia, and Balkans^61^
*G. taymyrensis* sp. nov.	Russia: Dudinka, small lake, 69.4008° N, 86.3384° E, Taymyr Peninsula	Taymyr Peninsula and Putorana Plateau	Siberia from the Arctic Ocean coast (Taymyr) to Kemerovo Region
**The *concolor*-group**			
*G. concolor* (Apáthy, 1888) = *Clepsine concolor* Apáthy (1888): 154^122^	Bei Neapel in dem Sebeto und in dem Sarno; bei Haraszti in einem Donau-arm^122^ (Italy: Sebéto and Sarno rivers near Naples, 40.8797° N, 14.3252° E and 40.7653° N, 14.5512° E, respectively; Hungary: a branch of Danube River near Dunaharaszti town, 47.3608° N, 19.0809° E)	Kolguev Island, Polar Urals, and Taymyr Peninsula	Arctic Region from Kolguev Island and Polar Urals to Taymyr; Siberia (including Lake Baikal); Kazakhstan; Iran^78^; European Russia; Sweden; Germany; Lithuania^58^, France^89^, Hungary and Italy (the type series; no topotype DNA sequences)
*G. nebulosa* Kalbe, 1964 = *G. complanata nebulosa* Kalbe (1964): 141^123^	Germany: Nieplitz River near Treuenbrietzen, 52.0911° N, 12.8666° E^123^	Polar Urals and Taymyr Peninsula	Arctic Region from Polar Urals to Taymyr; North Caucasus; Europe (Germany, France); Turkey: Antalya^10,89^
**The *verrucata*-group**			
*G. arctica* sp. nov.	Russia: a lake near Sob' railway station, 67.0480° N, 65.6316° E, Polar Urals	Polar Urals	Polar Urals (unknown beyond the type locality)
*G. mollissima* Moore, 1951 = *G. mollissima* Moore (1898): 547^74^ (identification error; reference to *Clepsine mollissima* Grube, 1871); = *G. mollissima* Livanow (1902): 353^42^ (assumption on a separate status of this taxon: ‘Moore's *Gl. mollissima* aber betrachte ich als eine vielleicht neue Art von *Glossosiphonia*’); = *G. complanata mollissima* Moore in Moore & Meyer (1951): 59^72^ (new combination with reference to *G. mollissima* Moore, 1898); = *G. camplanata mullissimi* Keith (1955): 104^73^ (erroneous spelling); = *Boreobdella verrucata* Klemm (1982): 110^124^ (identification error)	Russia: Bering Island, 55.22° N, 166.05° E, Commander Islands^42,72,74^; USA: shallow water of Thumb Lake on Kodiak Island, 57.3507° N, 153.9795° W, Alaska^72^	Taymyr Peninsula, Putorana Plateau, Arctic Yakutia, and Chukotka Peninsula	Arctic Region from Taymyr to Chukotka Peninsula; Russian Far East from Kamchatka and Commander Islands to Primorye; USA: SE Alaska and Kodiak Island^72,73^
*G. verrucata* (F. Müller, 1844) = *Clepsine verrucata* Müller (1844): 23^125^	Germany: Lake Tegel in Berlin, 52.5790° N, 13.2590° E^66^	Yamal Peninsula and Putorana Plateau	Yamal and Khanty-Mansi Region^70^ to Eastern Siberia (up to the Lena River basin)^69^; Europe: Scandinavia, the British Isles^10^, Poland, Lithuania^58^, Northern European Russia^15,67^, Germany^66^, and the Netherlands^68^ (DNA sequences of European populations are not available)
**Genus *Hyperboreomyzon* gen. nov.**			
*H. polaris* gen. & sp. nov.	Russia: small lake, 68.9008° N, 94.1599° E, Putorana Plateau	Kolguev Island and Putorana Plateau	Arctic Region from Eastern Europe to Eastern Siberia (known from three localities only)
**Subfamily Theromyzinae Sawyer, 1986**			
**Genus *Theromyzon* Philippi, 1867**			
*T. maculosum* (Rathke, 1862) = *Clepsine maculosa* Rathke (1862): 73^126^	“Bei Königsberg < … > nur in den Gräben gefunden, welche in der Nähe des Bahnhofes liegen”^126^ (Russia: Kaliningrad, ditches near the former East Railway Station, approx. 54.7030° N, 20.5018° E)	Yamal^24^, Taymyr, Iceland^16,17^	Europe to Eastern Siberia (including Lake Baikal); Iceland^16,17^; North Kazakhstan^84^; Tajikistan^86^
*T. tessulatum* (O. F. Müller, 1773) = *Hirudo tessulata* Müller (1773): 45^127^	Denmark^127^	Iceland^16^; Bolshezemelskaya Tundra^22^	Iceland^16^; Europe; North Asia^15^; North Kazakhstan^84^; Canada (Ontario)
**Subfamily Haementeriinae Autrum, 1939**			
**Genus *Helobdella* Blanchard 1896**			
*H. okhotica* sp. nov.	Russia: Khodeevskoye Lake, 64.7501° N, 177.7771° E, Chukotka Peninsula	Arctic Yakutia and Chukotka Peninsula	Lena River basin in Eastern Siberia to the Russian Far East
*H. stagnalis* (Linnaeus, 1758) = *Hirudo stagnalis* Linnaeus (1758): 649^128^	Sweden: north side of Lake Trehörningen, 59.8433° N, 17.8828° E, Uppsala, Uppsala County, Uppland Province (neotype)^129^	Arctic Norway, Iceland, Kanin Peninsula, Kolguev Island, Bolshezemelskaya Tundra, Polar Urals, and Taymyr Peninsula	Iceland, Europe, Siberia east to the Yenisey and Khatanga basins; Tajikistan; Uzbekistan; Azerbaijan; Iran^78^; Turkey^82^; North Africa (Tunisia, Morocco, Egypt)^79,96,120,121^; doubtful records from South Africa^81^

**Table 2:** Distribution of glossiphoniid leeches throughout subregions of the Arctic: (1) DNA-based records; (2) published and original records based on morphological criteria; and (–) the absence of a given species in samples.

Species	European Arctic and Iceland					Asian Arctic					Nearctic
	ICE	FEN	KAN	KOL	URA	YAM	TAY	PUT	YAK	CHU	GRE
*Alboglossiphonia heteroclita*	–	–	–	–	–	2	–	–	–	–	–
A. sibirica sp. nov.	–	–	–	–	–	2	–	–	–	–	–
Glossiphonia arctica sp. nov.	–	–	–	–	1	–	–	–	–	–	–
G. balcanica	2	1	1	1	1	1	1	–	–	–	–
G. concolor	–	–	1	1	1	–	1	–	–	–	–
G. mollissima	–	–	–	–	–	–	1	1	1	1	–
G. nebulosa	–	–	–	–	1	–	1	–	–	–	–
G. taymyrensis sp. nov.	–	–	–	–	–	–	1	1	–	–	–
G. verrucata	–	–	–	–	–	1	–	1	–	–	–
Helobdella okhotica sp. nov.	–	–	–	–	–	–	–	–	2	1	
H. stagnalis	1	1	1	1	1	2	1	–	–	–	–
Hyperboreomyzon polaris gen. & sp. nov.	–	–	–	1	–	–	–	1	–	–	–
Theromyzon maculosum	2	2	–	–	2	2	1	–	–	–	–
T. tessulatum	2	2	–	–	2	–	–	–	–	–	–
T. garjaewi groenlandicum	–	–	–	–	–	–	–	–	–	–	2
Total species richness	4	4	3	4	7	6	7	4	2	2	1

The subregion’s codes are as follows: Iceland (ICE); Fennoscandia: Arctic Scandinavia and Kola Peninsula (FEN); Kanin Peninsula and Malozemelskaya Tundra (KAN); Kolguev Island (KOL); Bolshezemelskaya Tundra (eastern part) and Polar Urals (URA); Yamal Peninsula (YAM); Taymyr Peninsula (TAY); Putorana Plateau (PUT); Arctic Yakutia (YAK); Chukotka Peninsula (CHU); and West Greenland (GRE). Additional sources of information: Iceland^16,17,130^; Fennoscandia^13,14,19,129^; Bolshezemelskaya Tundra (eastern part)^20,22^; Yamal^23,24^; Arctic Yakutia^25^; Chukotka Peninsula^25^; and West Greenland^10,46,47^.

### Subregional faunas and a preliminary biogeographic division of the Eurasian Arctic

The European Arctic (including Iceland) is characterized by a rather low species richness of the Glossiphoniidae, with only 3–4 species per subregion (Fig. 2, Table 2, and Supplementary Dataset S3). In particular, Iceland and Arctic Fennoscandia house four species: *Glossiphonia balcanica*, *Helobdella stagnalis*, *Theromyzon maculosum*, and *T. tessulatum*. In Eastern European Arctic, we also recorded *Glossiphonia concolor* and *Hyperboreomyzon polaris* gen. & sp. nov. but did not find *Theromyzon* spp., probably due to incomplete sampling. The eastern part of the Bolshezemelskaya Tundra and the Polar Ural Mountains harbor a richer fauna with seven species, including *Glossiphonia arctica* sp. nov., a possible endemic to the subregion. The Yamal Peninsula is inhabited by six species and it represents the only subregion, in which two *Alboglossiphonia* species, i.e., *A. heteroclita* and *A. sibirica* sp. nov., cross the Arctic Circle. The Taymyr Peninsula and the Putorana Plateau house the most species-rich fauna of glossiphoniid leeches in the Arctic, which contains as many as nine species. The most species-poor Arctic faunas of the Glossiphoniidae are recorded from northeastern Asia and Greenland. In particular, Arctic Yakutia east of the Lena River basin and the Chukotka Peninsula harbor only two species: *Glossiphonia mollissima* and *Helobdella okhotica* sp. nov. Based on published data, only one *Theromyzon* species was recorded from West Greenland (Tables 1, 2).

Our preliminary biogeographic analysis based on the presence-absence data on glossiphoniid leeches indicates that the Eurasian Arctic is the area where the marginal parts of two large divisions, i.e., the Western and Eastern Palearctic subregions, meet (Fig. 2b). The boundary between these parts is located between the Lena River basin and the Kolyma Highland (Arctic Yakutia). The Western Palearctic Subregion embraces the continuous Euro-Siberian Province (from Iceland and Scandinavia to the Yenisey River) (Fig. 2c,d) and the Taymyr Province (Taymyr Peninsula and Putorana Plateau) (Fig. 2e,f). The Eastern Palearctic Subregion covers the Kolyma Highland and Chukotka Peninsula (Fig. 2g).

### Distribution ranges and biogeographic affinities of Arctic glossiphoniid leeches

The majority of glossiphoniid leech species recorded north of the Arctic Circle are characterized by broad ranges, crossing a number of climatic zones (Figs. 3a, 7a–d, and Supplementary Figs. S12, S13). Conversely, two species, *Glossiphonia arctica* sp. nov. and *Hyperboreomyzon polaris* gen. & sp. nov. are known to exclusively occur in the Arctic areas (Fig. 7c).

From a biogeographical point of view, most of the Eurasian Arctic glossiphoniid leeches share clear European affinities: *Alboglossiphonia heteroclita*, *Glossiphonia balcanica*, *G. concolor*, *G. nebulosa*, *G. verrucata*, *Helobdella stagnalis*, and *Theromyzon maculosum* (Tables 1, 2 and Supplementary Fig. S12). In contrast, the ranges of *Alboglossiphonia sibirica* sp. nov., *Helobdella okhotica* sp. nov., and *Glossiphonia taymyrensis* sp. nov. are likely confined to North Asia. The first species is widespread from the Ob Basin to the Russian Far East (including the Amur River) (Fig. 7a). *Helobdella okhotica* sp. nov. has a more eastern range, expanding from the Lena River basin to the Far East (Fig. 7d). *Glossiphonia taymyrensis* sp. nov. appears to have a Siberian range, as it was recorded from Western and Eastern Siberia only (Fig. 7b). Two species, *Theromyzon tessulatum* and *Glossiphonia mollissima*, have Holarctic distribution (Table 1 and Supplementary Fig. S13). The first taxon shares a broad but discontinuous range in Eurasia and North America, while *G. mollissima* can be considered a Beringian species because it was found in northeastern Asia, on the Commander Archipelago and Kodiak Island, and in Alaska. *Glossiphonia arctica* sp. nov. is unknown beyond its type locality situated at the northern edge of the Ural Mountain Range, while *Hyperboreomyzon polaris* gen. & sp. nov. most likely has a North Asian (Siberian) affinities. In both cases, available occurrence data are very limited (Fig. 7c) that precludes any final biogeographic solution on the ranges of these taxa.

### Discovery of melanic forms in Arctic Glossiphoniidae

**Figure 8:**
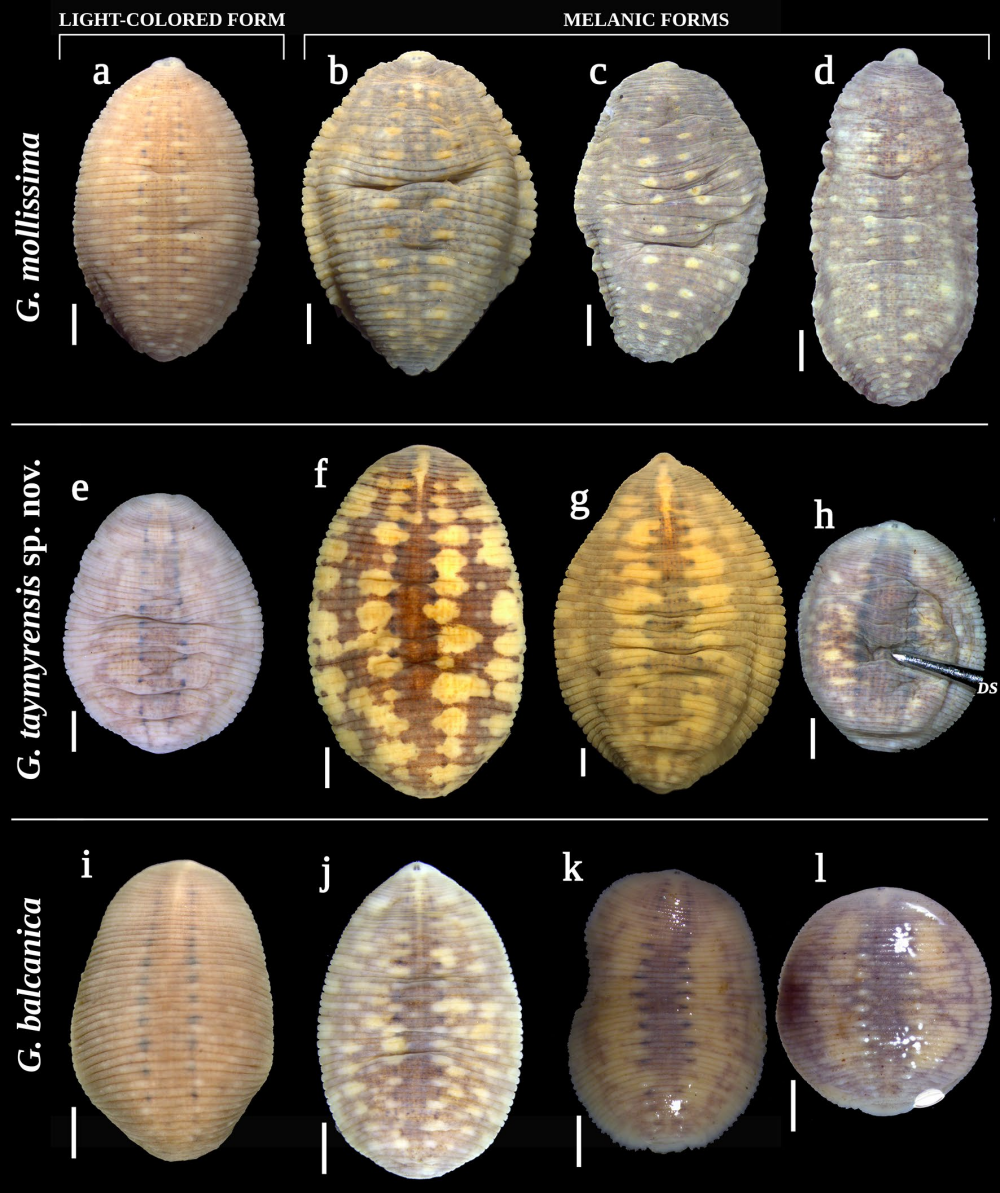
Examples of light-colored and melanic color forms of *Glossiphonia* spp. from the Eurasian Arctic (dorsal view). (**a**–**d**) *G. mollissima*, Chokurdakh, Arctic Yakutia: (**a**) RMBH Hir_0480_2 (light-colored form); (**b**) RMBH Hir_0478, (**c**) RMBH Hir_0483_2 and (**d**) RMBH Hir_0482_2 (‘darker’ melanic forms with reduced markings). (**e**–**h**) *G. taymyrensis* sp. nov.: (**e**) RMBH Hir_0264_3 (light-colored form); (**f**) RMBH Hir_0256_1, Dudinka, Taymyr Peninsula and (**g**) RMBH Hir_0488, Putorana Plateau (melanic forms: f. ‘maculosa’); and (**h**) RMBH Hir_0256_1, Dudinka, Taymyr Peninsula (‘darker’ melanic form with highly reduced markings). (**i–l**) *G. balcanica*: (**i**) RMBH Hir_0250 (light-colored form), Teriberka, Kola Peninsula; (**j**) RMBH Hir_0 457_4 (melanic form: f. ‘maculosa’), Sob’ railway station, Polar Urals; (**k**) RMBH Hir_0081_1 and (**l**) RMBH Hir_0083, Kharp, Polar Urals (‘darker’ melanic forms with reduced markings and darkened yellow areas). *DS* dissecting needle. Scale bars = 1.0 mm. Photos: Anna L. Klass and Tatyana A. Eliseeva; graphics: Ivan N. Bolotov.

Here, we present the record of a melanic form of *T. maculosum* from the Taymyr Peninsula (Figs. 3g, 4i). The characteristic light markings pattern is highly reduced or lacking in such dark individuals. All available specimens of *Hyperboreomyzon polaris* gen. & sp. nov. have a dark melanic coloration, which, possibly, is typical for this taxon, with the darkest form collected from the Putorana Plateau (Fig. 4j). High-latitude melanic forms were frequently recorded in two members of the *Glossiphonia complanata*-group: *G. balcanica* (22.7%; 5 melanic and 17 light-colored specimens) and *G. taymyrensis* sp. nov. (77.8%; 7 melanic and 2 light-colored specimens) (Fig. 8). Additionally, two representatives of the *verrucata*-group also have melanic forms in the Arctic: *G. mollissima* (56.5%; 26 melanic and 20 light-colored specimens) and *G. verrucata* (100%; 2 melanic specimens) (Figs. 4c, 8). Conversely, high-latitude melanic forms were not recorded in the examined Arctic samples of *Glossiphonia arctica* sp. nov. (*N* = 19)*, G. concolor* (*N* = 45), and *G. nebulosa* (*N* = 31). Finally, melanism is not expressed in *Alboglossiphonia* and *Helobdella* species.

## Taxonomic account

### Suborder Glossiphoniiformes Tessler & de Carle, 2018

#### Family Glossiphoniidae Vaillant, 1890

*Comments*. Our two-locus phylogeny reveals the presence of two large clades, corresponding to the subfamilies Glossiphoniinae and Haementeriinae (Fig. 1). The subfamily Theromyzinae Sawyer, 1986, delineated by some authors^2,10,22^, was not supported as a distant phylogenetic clade and their representatives are clustered within the monophyletic Glossiphoniinae. The same pattern was recovered by earlier phylogenetic reconstructions^3,30,33,34^. These data indicate that Theromyzinae may represent a synonym of the latter subfamily. However, a subfamily-level revision of the Glossiphoniidae is beyond the framework of the present study.

### Subfamily Glossiphoniinae Vaillant, 1890

#### Genus *Alboglossiphonia* Lukin, 1976

Type species: *Alboglossiphonia heteroclita* (Linnaeus, 1761) (= *Hirudo heteroclita* Linnaeus, 1761; by original designation).

*Arctic occurrences*. Our results reveal that members of this genus are not common inhabitants of the Arctic but two species, *A. heteroclita* (Linnaeus, 1761) and *A. sibirica* sp. nov., cross the Arctic Circle on the Yamal Peninsula through the Ob and Taz rivers (Table 1). Previously, it was shown that *A. heteroclita* occurs in the lower Ob Basin, northern edge of Western Siberia^23^.

*Comments*. This genus contains inconspicuous minute leeches and is characterized by a nearly global distribution^1^. It definitely requires an integrative taxonomic revision. Available genetic evidence (Fig. 1 and Supplementary Fig. S1) reveals that the North American populations of what was traditionally assigned to *A. heteroclita* should be considered a separate species, *A. pallida* (Verrill, 1872) (type locality: West River near New Haven, Connecticut, USA)^35,36^. Other species, which occurs in Siberia and the Far East, was tentatively assigned to *Alboglossiphonia* cf. *papillosa* (Braun, 1805) based on a darker pigmentation of its dorsum^37,38^ but it represents a separate North Asian species, which is described here.

### *Alboglossiphonia sibirica* Bolotov, Eliseeva, Klass & Kondakov sp. nov

 = *Alboglossiphonia heteroclita* Lukin (1957): 273^39^ (identification error).

 = *Alboglossiphonia heteroclita papillosa* Kaygorodova et al. (2014): 3^37^; Kaygorodova (2015): 41^40^ (identification error).

 = *Alboglossiphonia* cf. *papillosa* Klass et al. (2018): 26^38^ (identification error).

Figures 4a, 5a, 7a, Supplementary Figs. S2a, S3a, S4, Supplementary Table S2.

LSID: https://zoobank.org/urn:lsid:zoobank.org:act:19B581C3-E912-487C-B9EC-8E50DDEFD380.

*Holotype*. RMBH Hir_0542_2-H (non-sequenced), RUSSIA: Lake Torfyanka, 43.0761° N, 131.9620° E, Vladivostok, Primorye, August 12, 2021, Y. E. Chapurina leg.

*Paratypes* (*N* = 13). RUSSIA: 1 specimen RMBH Hir_0542_2 (sequenced: *COI* sequence acc. No. ON873332), the type locality, the same date, and collector; 1 specimen RMBH Hir_0396 (non-sequenced), an oxbow lake of Taz River, near Tazovsky settlement, 67.5063° N, 78.6751° E, Yamal-Nenets Region, August 22, 2019, E. S. Babushkin leg.; 1 specimen RMBH Hir_0394 (DNA voucher; sequenced: *COI* sequence acc. No. ON548508), Vitim River, 57.2010° N, 116.4300° E, Lena River basin, Vitimsky Nature Reserve, Irkutsk Region, July 12, 2019, E. S. Babushkin leg.; 4 specimens RMBH Hir_0013 (3 sequenced with DNA vouchers and one placed on 36 permanent slides as a series of slices; *COI* sequence acc. No. MH286267, MH286268, and MH286269; *18S rRNA* sequence acc. No. MH286273), between zooids of a bryozoan colony, small floodplain lake of the Lena River near Yakutsk, 62.3076° N, 129.8999° E, Yakutia Republic, August 20, 2017, I. N. Bolotov leg.; 1 specimen RMBH Hir_0417_2 (DNA voucher; sequenced: *COI* sequence acc. No. ON548511), Oron Lake, Gnilaya Kurya Bay, 57.1750° N, 116.4031° E, Lena River basin, Vitimsky Nature Reserve, Irkutsk Region, July 1, 2019, E. S. Babushkin leg.; 1 specimen RMBH Hir_0409_1 (sequenced: *COI* sequence acc. No. ON548509), a roadside ditch in Knevichi settlement, 43.3886° N, 132.1880° E, Primorye, September 10, 2020, O. V. Aksenova et al. leg.; 1 specimen RMBH Hir_0413 (sequenced: *COI* sequence acc. No. ON548510), a puddle near railway at Artem city, 43.3794° N, 132.2188° E, Primorye, September 10, 2020, O. V. Aksenova et al. leg.; 1 specimen RMBH Hir_0003_3 (DNA voucher; sequenced: *COI* sequence acc. No. MN393256), Tumnin River, 49.9451° N, 139.9181° E, Khabarovsk Region, July 14, 2014, I. N. Bolotov & I. V. Vikhrev leg.; 1 specimen RMBH Hir_0509_1 (sequenced: *COI* sequence acc. No. ON548516), a reservoir on the Bolshoy Alim River, near Tolstovka settlement, 50.1981° N, 127.9431° E, Amur Region, July 3, 2021, O. V. Aksenova et al. leg.; 1 specimen RMBH Hir_0510_1 (DNA voucher; sequenced: *COI* sequence acc. No. ON548517), an oxbow lake of Bureya River, near Novospassk, 49.6756° N, 129.7343° E, Amur Region, July 3, 2021, O. V. Aksenova et al. leg.

*Etymology*. The name of this species reflects its broad distribution in Siberia.

*Differential diagnosis*. Small leech, which could be distinguished from other congeners by a combination of the following characters: dorsum covered by numerous small, shallow, and indistinct papillae, light yellow, with multiple dark spots and short dashes arranged to 18–24 longitudinal rows; these spots and dashes merged into longitudinal lines in the anterior half of the body (the dark markings pattern often lost in ethanol-preserved animals). Externally, the new species is similar to *A. heteroclita*, *A. hyalina* (O. F. Müller, 1773), and *A. striata* (Apáthy, 1888). However, all these species do not have numerous dark spots and short dashes arranged to multiple longitudinal rows. Additionally, *A. heteroclita* differs from the new species by having a median row of segmentally arranged dark spots and a smooth dorsum without papillae. *A. hyalina* differs from *A. sibirica* sp. nov. by the general lack of dark pigmentation. *A. striata* differs from the new species by having a median row of segmentally arranged dark transverse stripes and a smooth dorsum without papillae.

*Molecular diagnosis*. The new species represents a separate genetic lineage but is more closely related to *A. heteroclita* (mean pairwise *COI* p-distance = 5.1%; range 4.9–5.4%). The intraspecific pairwise *COI* p-distance ranges from 0.0 to 2.1% (mean ± s.e.m. = 1.31 ± 0.10%; *N* = 14 sequences and 91 pairwise distance values). The GenBank acc. numbers of reference DNA sequences (*COI* and *18S rRNA*) are given in Supplementary Table S2 and Supplementary Datasets S1–S2.

*Description*. Small leech (body length up to 11.9 mm). Measurements of the type series are given in Supplementary Table S2. Body broad, leaf-like, ovate. Dorsum with numerous small, shallow, and indistinct papillae. Posterior sucker small, circular (maximum diameter of 2.25 mm), ventrally directed. Proboscis pore in the center of anterior sucker. Coloration of living animals: body dirty yellow with multiple brown spots and dashes arranged to longitudinal rows; in the anterior half of the body, these spots and dashes merged into longitudinal lines. Coloration of ethanol-preserved animals: body light yellow; dorsum with multiple dark spots and short dashes arranged to 18–24 longitudinal rows; these spots and dashes merged into longitudinal lines in the anterior half of the body but the dark markings pattern often lost due to preservation. Three pairs of eyespots; the eyespots of the distal pair joined into a single spot; the eyespots of the next two pairs are spaced apart and fused together. Venter light yellow or whitish. Total number of annuli: 70. Somites I–IV joined to form a head region, somites V–XXIV triannulate, somites XXV–XXVII uniannulate. Gonopores joined and open in the furrow XIIa1/a2. Reproductive system: 6 pairs of large, bag-like testisacs inter-segmentally from XIII/XIV to XIX/XX; atrium small, spherical, the atrial cornua twisted anteriorly; paired ejaculatory ducts twisted, short; paired ovisacs narrow, very short. Digestive system: proboscis sheath massive, long, thick; salivary glands diffuse; crop with 6 pairs of crop caeca: 1st-5th uniform, bag-like, 6th pair (posterior caeca) with 3 blind processes; intestine with 4 pairs of rather short processes and an ovate extention after the last pair of processes.

*Distribution*. North Asia: Western Siberia, Eastern Siberia, the Russian Far East, and Mongolia^39^.

*Habitats and ecology*. This species is known to occur in a broad range of freshwater environments such as rivers, oxbow lakes, large to small natural lakes, reservoirs, road ditches, and even puddles (Supplementary Dataset S2). An unusual example of its association with a bryozoan species was described from Eastern Siberia^38^. Probably, the record of an *Alboglossiphonia* leech in the mantle cavity of an unidentified lymnaeid snail from the Altai Mountains, Russia^41^ could also be attributed to this species. The life cycle of the new species is unknown.

#### Genus *Glossiphonia* Johnson, 1816

Type species: *Glossiphonia complanata* (Linnaeus, 1758) (= *Hirudo complanata* Linnaeus, 1758; by subsequent designation).

*Arctic occurrences*. Representatives of this genus are the most remarkable component of the Arctic Glossiphoniidae fauna. Altogether seven species were recorded north of the Arctic Circle, two of which are new to science and described here (Table 1).

*Comments*. In general, sequenced representatives of the genus *Glossiphonia* could phylogenetically be delineated to three species groups (or subgenera): (1) the *complanata*-group (= subgenus *Glossiphonia* s. str.); (2) the *verrucata*-group (= subgenus *Boreobdella* Johansson, 1929; type species: *Clepsine verrucata* Müller, 1844); and (3) the *concolor*-group (= subgenus *Paratorix* Lukin & Epstein, 1960; type species: *Torix baicalensis* Stschegolew, 1922) (Table 1, Fig. 1 and Supplementary Fig. S1).

### *Glossiphonia arctica* Bolotov, Eliseeva, Klass & Kondakov sp. nov

Figures 4B, 5b,c, 7c, Supplementary Figs. S2b, S3b, S5, Supplementary Table S2.

LSID: https://zoobank.org/urn:lsid:zoobank.org:act:FADF0993-A946-413A-9680-25BA0F9BE90D.

*Holotype*. RMBH Hir_0457_2_1-H (sequenced: *COI* sequence acc. No. ON810735; *18S rRNA* sequence acc. No. ON819028), RUSSIA: a large lake near Sob' railway station, 67.0480°N, 65.6316°E, Polar Urals, June 23, 2021, A. V. Kondakov et al. leg.

*Paratypes* (*N* = 18). 18 specimens RMBH Hir_0457 (two specimens sequenced: *COI* sequence acc. No. ON810736 and ON810737; *18S rRNA* sequence acc. No. ON819029; one specimen placed on 18 permanent slides as a series of slices), the type locality, the same date, and collectors.

*Etymology*. The name of the new species indicates that its type locality is situated in the Arctic Region.

*Differential diagnosis*. Medium-sized leech, which could be distinguished from other congeners by a combination of the following characters: dorsum with four rows of ovate, broad but very shallow and indistinct papillae on annulus a2 (outer paramedian and inner paramarginal series); each papilla bears ovate light yellow or white spot; dorsal black markings pattern absent. Externally, the new species is similar to *G. mollissima*. However, the latter species differs from *G. arctica* sp. nov. by having larger papillae and a well-developed black markings pattern dorsally.

*Molecular diagnosis*. The new species represents a separate genetic lineage belonging to the *verrucata*-group (Fig. 1). The pairwise *COI* p-distance of the new species from other congeners varies from 7.0 to 12.4%. The intraspecific pairwise *COI* p-distance ranges from 0.0 to 0.2% (mean ± s.e.m. = 0.10 ± 0.05%; *N* = 3 sequences and 3 pairwise distance values). The GenBank acc. numbers of reference DNA sequences (*COI* and *18S rRNA*) are given in Supplementary Table S2 and Supplementary Datasets S1–S2.

*Description*. Medium-sized leech (body length up to 13.3 mm). Measurements of the type series are given in Supplementary Table S2. Body broad, leaf-like, ovate. Dorsum with four rows of ovate, broad but very shallow and indistinct papillae on annulus a2 (outer paramedian and inner paramarginal series). Posterior sucker small, circular (maximum diameter of 1.9 mm), ventrally directed. Proboscis pore in the center of anterior sucker. Coloration of living animals: body almost transparent, light brown, with multiple yellowish pigment cells. Coloration of ethanol-preserved animals: dorsum beige to light brown, with darker broad inner paramedian lines and light yellowish areas laterally and anteriorly; ovate light yellow or white spots at each papillae on annulus a2 arranged into four longitudinal rows (outer paramedian and inner paramarginal), sometimes with a few white spots between them. Three pairs of ovate eyespots arranged to two parallel rows; in some specimens eyes on each side are joined to a single large spot. Venter whitish to light brown, sometimes with irregular brownish shading. Total number of annuli: 70. Somites I–III uniannulate, IV biannulate, V–XXIV triannulate, XXV biannulate, XXVII uniannulate. The male and female genital pores are separated by two annuli and are located in the furrows XIa3/XIIa1 and XIIa2/a3, respectively. Reproductive system: 6 pairs of spherical testisacs inter-segmentally from XIII/XIV to XVIII/XIX; atrium spherical, the atrial cornua large, twisted anteriorly; paired ejaculatory ducts very long, extending to XVIII; paired ovisacs massive, long, with multiple lobes, arranged as loops, extending to XVIII (pregnant specimen with eggs). Digestive system: proboscis sheath massive, thick, elongated; esophagus narrow; salivary glands diffuse; crop with 7 pairs of crop caeca: 1st-6th uniform, bag-like, 7th pair (posterior caeca) with 4 blind processes and several smaller lobes; intestine enlarged, with 4 pairs of large, long, bag-like processes, expanding distally, each with several short lobes; a large circular extension after the last pair of processes.

*Distribution*. Polar Urals (not known beyond the type locality).

*Habitats and ecology*. The type series of this species was collected from a natural mountain lake with stony bottom. The leeches were recorded beneath flat stones (Fig. 3b); their feeding behavior and life cycle remain unknown.

### *Glossiphonia taymyrensis* Bolotov, Eliseeva, Klass & Kondakov sp. nov

Figures 4E, 5d, 7b, Supplementary Figs. S2h, S3c, S6, Supplementary Table S2.

LSID: https://zoobank.org/urn:lsid:zoobank.org:act:40269BF4-FE1C-4269-A7CC-41020789DC44.

*Holotype*. RMBH Hir_0258_1-H (sequenced: *COI* sequence acc. No. ON810695), RUSSIA: small lake near Dudinka on Taymyr Peninsula, 69.4008°N, 86.3384°E, July 16, 2018, O. V. Aksenova et al. leg.

*Paratypes* (*N* = 8). RUSSIA: 2 specimens RMBH Hir_0263_1 and RMBH Hir_0264_3 (sequenced: *COI* sequence acc. No. ON810701 and ON810705; *18S rRNA* sequence acc. No. ON819017), the type locality, the same date, and collectors; 2 specimens RMBH Hir_0256_1 (one sequenced and one placed on 20 permanent slides as a series of slices; *COI* sequence acc. No. ON810693), small lake near Dudinka on Taymyr Peninsula, 69.3987° N, 86.3505° E, July 16, 2018, O. V. Aksenova et al. leg.; 1 specimen RMBH Hir_0261_2 (sequenced: *COI* sequence acc. No. ON810699; *18S rRNA* sequence acc. No. ON819016), small lake near Dudinka on Taymyr Peninsula, 69.4014° N, 86.3250° E, July 16, 2018, O. V. Aksenova et al. leg.; 1 specimen RMBH Hir_0265_2 (sequenced: *COI* sequence acc. No. ON810706; *18S rRNA* sequence acc. No. ON819021), Bolgokhtokh River near Dudinka, Taymyr Peninsula, 69.3780° N, 87.2215° E, July 21, 2018, O. V. Aksenova et al. leg.; 1 specimen RMBH Hir_0488 (sequenced: *COI* sequence acc. No. ON810755), a lake on Putorana Plateau, 68.7607° N, 91.9014° E, July, 2021, E. S. Chertoprud leg.; 1 specimen RMBH Hir_0449 (sequenced: *COI* sequence acc. No. ON810731), Pyzas River near Ust-Kabyrza settlement, 52.8277° N, 88.3973° E, Tashtagolsky District, Kemerovo Region, July 23, 2020, E. S. Babushkin & M. V. Vinarski leg.

*Etymology*. The new species is named after the Taymyr Peninsula, where the majority of the type specimens were collected.

*Differential diagnosis*. Small leech with broad, leaf-like, ovate body; three pairs of eyespots (distal pair joined; next two pairs separate); dorsal papillae absent; dorsal coloration with two inner paramedian rows of black spots, sometimes joining into unclear dashed lines; two annuli between the male (XIa3/XIIa1) and female (XIIa2/a3) genital pores. The new species largely resembles *G. complanata* but could be distinguished from it by having a smooth dorsum, without clear papillae. These taxa seem to have non-overlapping, allopatric ranges and, hence, could be separated on the basis of geographic criteria. However, the DNA approach seems to be the most appropriate way to distinguish these two species.

*Molecular diagnosis*. The new species represents a separate genetic lineage belonging to the *complanata*-group (Fig. 1). The pairwise *COI* p-distance of the new species from other congeners varies from 6.0 to 12.2%. The intraspecific pairwise *COI* p-distance ranges from 0.0 to 1.1% (mean ± s.e.m. = 0.52 ± 0.07%; *N* = 8 sequences and 28 pairwise distance values). The GenBank acc. numbers of reference DNA sequences (*COI* and *18S rRNA*) are given in Supplementary Table S2 and Supplementary Datasets S1–S2.

*Description*. Small leech (body length up to 11.3 mm). Measurements of the type series are given in Supplementary Table S2. Body broad, leaf-like, ovate. Dorsum smooth, without clear papillae. Posterior sucker ovate (maximum diameter of 3.0 mm), ventrally directed. Proboscis pore in the center of anterior sucker. Coloration of living animals: not examined. Coloration of ethanol-preserved animals: (1) typical form having beige to light brown ground color without light spots but with darker brown coloration between inner paramedian lines; (2) melanic forms having dark brown ground color with four rows of large yellow spots (outer paramedian and marginal series) and yellow median stripe anteriorly (f. ‘maculosa’) or with strongly reduced yellow markings pattern. In all forms, there are two inner paramedian rows of black spots, sometimes joining into unclear dashed lines. Three pairs of ovate eyespots; the eyespots of the distal pair joined into a single spot; the eyespots of the next two pairs separate and are spaced apart. In the typical form, venter light yellow, with paired brown median and outer paramedian lines, which may be reduced to series of narrow brown longitudinal stripes. In melanic forms, ventral markings is more developed, with a series of brown longitudinal lines from median to inner paramarginal position and outer paramarginal brown spots. Posterior sucker with dense brown spots in melanic forms and with scarce brown spots in typical form. Total number of annuli: 68. Somites I–IV uniannulate, V–XXIV triannulate, XXV biannulate, XXVI–XXVII uniannulate. The male and female genital pores are separated by two annuli and are located in the furrows XIa3/XIIa1 and XIIa2/a3, respectively. Reproductive system: 6 pairs of spherical testisacs inter-segmentally from XII/XIII to XVIII/XIX; atrium ovate, the atrial cornua directed laterally; paired ejaculatory ducts twisted, short; paired ovisacs short, thick (undeveloped). Digestive system: salivary glands diffuse; proboscis sheath moderately thick; esophagus ovate; crop with 6 pairs of massive, bag-like, uniform crop caeca; intestine with 4 pairs of processes.

*Distribution*. Western and Eastern Siberia.

*Habitats and ecology*. The new species was recorded from natural lakes and rivers (Supplementary Dataset S2); its feeding behavior and life cycle are unknown.

#### Genus *Hyperboreomyzon* Bolotov, Eliseeva, Klass & Kondakov gen. nov

LSID: https://zoobank.org/urn:lsid:zoobank.org:act:298FF41E-AF0D-4442-9F82-3022B8094A67.

Type species: *Hyperboreomyzon polaris* gen. & sp. nov.

*Etymology*. This name is compiled using two Greek words: ‘*Hyperborea*’ (meaning a mythical far northern land) and ‘*myzon*’ (meaning sucking).

*Diagnosis*. Medium-sized, elongate, sub-fusiform glossiphoniid leeches; body and posterior sucker densely covered by shallow, 'fish-scale'-like papillae; somite V biannulate; somites XII–XXIII secondarily sexannulate dorsally and ventrally due to the presence of very deep, prominent furrows separating each annulus to two semi-annuli; six rows of prominent dorsal tubercle-like papillae at a2 (inner paramedian, inner paramarginal, and marginal series) from V to XXVI; two pairs of circular eyespots on II and Va1 at inner paramedian position; gonopores at the furrows XIa3/XIIa1 (male) and XIIa2/a3 (female) and separated by two annuli; male atrium spherical; proboscis pore opens in a thick velar fold in the anterior half of oral sucker; one pair of compact, massive, elongated, incurved salivary glands, each gland with a bunch of a few short processes apically; 9 crop caeca pairs. Comparison of the new genus with other genera in the family based on morphological and anatomical features is presented in Supplementary Table S3. Sexannulate condition was also recorded in the genus *Actinobdella* Moore, 1901 from North America^36^, but it differs from *Hyperboreomyzon* gen. nov. by having one pair of eyespots, diffuse salivary glands, and an apical position of proboscis pore (Supplementary Table S3).

*Comments*. This genus is established for a single species, which is described below.

### *Hyperboreomyzon polaris* Bolotov, Eliseeva, Klass & Kondakov gen. & sp. nov.

Figures 4J, 5e, 6a-j, 7c, Supplementary Figs. S21, S8, S9, S10, S11, Supplementary Table S2.

LSID: https://zoobank.org/urn:lsid:zoobank.org:act:503A9A26-CEDE-4747-952D-8416AE4EF4EB.

*Holotype*. RMBH Hir_0486-H (sequenced: *COI* sequence acc. No. ON810753; *18S rRNA* sequence acc. No. ON819030), RUSSIA: small alpine lake on Putorana Plateau, 68.9008°N, 94.1599°E, July, 2021, E. S. Chertoprud leg.

*Paratypes* (*N* = 2). RUSSIA: 1 specimen RMBH Hir_0689 (dissected and placed on 60 permanent slides as a series of slices), small alpine lake on Putorana Plateau, 68.6659° N, 93.1365° E, August 11, 2021, E. S. Chertoprud leg.; 1 specimen RMBH Hir_0216 (sequenced and dissected; *COI* sequence acc. No. ON810677; *18S rRNA* sequence acc. No. ON819005), water puddle on Kolguev Island, 68.9300° N, 49.0303° E, August 12, 2018, O. V. Travina & V. M. Spitsyn leg.

*Etymology*. The name of the new species reflects its occurrences in polar (Arctic) areas of Eurasia.

*Differential diagnosis*. As for the genus.

*Molecular diagnosis*. None of congeneric species is known. Based on uncorrected pairwise *COI p*-distances between a haplotype of the new taxon and selected species-level haplotypes in each genus (Supplementary Table S1), *Hyperboreomyzon* seems to be more closely related to members of *Hemiclepsis* (mean distance ± s.e.m. = 11.62 ± 0.15%, range = 9.75–14.08%, *N* = 9) and *Theromyzon* (mean distance ± s.e.m. = 11.37 ± 0.07%, range = 10.47–12.64%, *N* = 9) without significant differences between distances from these two genera (Mann–Whitney test: *p* = 0.72). Other Glossiphoniidae genera are more distantly related, with a mean pairwise uncorrected *COI p*-distance of > 13.0% (Mann–Whitney test: *p* < 0.001) (Figure S14). In our two-locus phylogeny, it clusters with the *Theromyzon* and *Placobdelloides* clades but with moderate support (BS = 63) (Fig. 1). The intraspecific pairwise *COI* p-distance between two available sequences of this species is 0.3%. The GenBank acc. numbers of reference DNA sequences (*COI* and *18S rRNA*) are given in Supplementary Table S2 and Supplementary Datasets S1–S2.

*Description*. Medium-sized leech (body length up to 20.6 mm). Measurements of the type series are given in Supplementary Table S2. Body massive, elongated, sub-fusiform, with rounded anterior and posterior ends, dorsum convex, venter slightly concave, lateral margins of the body slightly arched ventrally. The entire body densely covered by shallow, 'fish-scale'-like papillae; dorsum with six rows of prominent tubercle-like papillae at a2 (inner paramedian) extending from V to XXVI. Lip small, rounded, with a deep median fold; proboscis pore opens in a massive velar fold in oral sucker close to its anterior margin; posterior sucker circular, small (maximum diameter of 3.4 mm), ventrally directed, dorsally covered by shallow, 'fish-scale'-like papillae, similar to those on the body. Coloration of living animals: not examined. Coloration of ethanol-preserved animals: dorsum brown to dark brown, with a spotted coloration consisted of dense brown pigment cells; six rows of circular yellow to dark orange spots on each dorsal tubercle-like papilla (inner paramedian, inner paramarginal, and marginal; located on a2); two pairs of small, circular eyespots in inner paramedian position: the distal pair on II, the proximal pair on Va1. Venter light brown to brown, with a spotted coloration like that on the dorsum and with a light yellow median line. Posterior sucker light brown with dense dark brown spots, forming a reticulate pattern. Total number of annuli: 68. Somite I merged with prostomium; II–IV uniannulate, V biannulate and forms posterior margin of anterior sucker, VI–XXIV triannulate (XII–XXIII secondarily sexannulate dorsally and ventrally due to the presence of deep, prominent furrows separating each annulus to two semi-annuli), XXV–XXVI biannulate, XXVII uniannulate. The male and female genital pores are separated by two annuli and are located in the furrows XIa3/XIIa1 and XIIa2/a3, respectively. Reproductive system: 6 pairs of spherical testisacs inter-segmentally from XIII to XIX; male atrium spherical, the atrial cornua distinctly narrowed, elongated, slightly tapering distally, laterally directed; a prominent dumbbell-shaped patch of black tissue of unknown functionality at the dorsal surface of atrium in XII (between XIIa1 and a3); paired ejaculatory ducts narrow, long, extending to XIX; paired ovisacs massive, elongated, convoluted thin-walled structures, arranged as loops, extending from XII to XIV. Digestive system: proboscis sheath narrow, long; esophagus narrow; salivary cells aggregated to a pair of compact, massive, elongated, incurved glands in X, each gland bears a bunch of 2–3 short processes apically; crop with 9 pairs of crop caeca: 1st-8th uniform, 9th pair forms posterior caeca with 4 blind processes; intestine with 4 pairs of simple processes.

*Distribution*. Known from Kolguev Island and Putorana Plateau only.

*Habitats and ecology*. Three available specimens were collected from alpine lakes on the Putorana Plateau (altitude 426 and 524 m a.s.l.) and from a water puddle in plain tundra on the Kolguev Island (altitude 30 m a.s.l.). Feeding behavior, hosts, and life cycle are unknown.

### Subfamily Theromyzinae Sawyer, 1986

#### Genus *Theromyzon* Philippi, 1867

Type species: *Theromyzon pallens* Philippi, 1867 (by monotypy).

*Arctic occurrences*. Two species in this genus are commonly occur in the Arctic, that is, *Theromyzon maculosum* (Rathke, 1862) and *T. tessulatum* (O. F. Müller, 1773).

*Comments*. Traditionally, nominal taxa in this genus were classified based on the number of annuli between gonopores^15,42,43^. This morphology-based concept is largely supported by our new DNA data. In particular, there are two phylogenetic species groups: (1) the *maculosum*-group with *T. maculosum* and a few related species from North America and the Caucasus Mountains (two annuli between gonopores); and (2) the *tessulatum*-group with *T. tessulatum* (four annuli) and *T. mollissimum* Grube, 1871 (five annuli). Foote et al.^44^ suggested that the number of annuli separating the gonopores can hardly be used as a species-level distinguishing feature for Nearctic members of the genus *Theromyzon* but, according to our combined two-locus phylogeny (*COI* + *18S rRNA*), it seems to be a reliable character to separate taxa between the two species groups, mentioned above (Fig. 1).

The nominal taxon *Placobdella raboti* Blanchard, 1893 was described from the Scandinavian Arctic (type locality: ‘l'Ivalojoki, rivière tributaire du lac Enara, par 25° de longitude est et entre 68 et 69° de latitude nord (Laponie finlandaise)’ = Finland: Ivalo River, a tributary of Lake Inari, approx. 68.442° N, 25.000° E, Northern Lapland)^13^. Lukin^15^ considered it as a synonym of *Placobdella costata* (F. Müller, 1846). However, this issue is not so straightforward both morphologically and geographically. This species does not have a median row of papillae, which is a characteristic feature of *Placobdella costata*. Moreover, records of the latter (pond turtle-feeding) species north of the Arctic Circle are hardly expected. Based on genetically confirmed records, the northern portion of its range expands throughout northern Germany, Latvia, Ukraine, and Southern European Russia^45^. Briefly, the holotype (fixed by monotypy) of *Placobdella raboti* is a medium-sized ethanol-preserved specimen (body length 17 mm, body width 9.5 mm) of ovate shape, with small but deep posterior sucker (width 3 mm); proboscis pore opens on the rim of the anterior sucker (anterior lip); body thin, dorsal side convex, ventral side deeply concave; six rows of large dorsal papillae from somite XII to the last annuli, with marginal rows being smaller and less prominent; the edge of each a2 slightly rises apically, leading to a very particular scalloped aspect; body color uniformly grey-brown but with lighter ventral side and lighter dorsal papillae; one pair of eyespots; gonopores between two annuli; total number of annuli 67^13^. These features correspond well to a melanic form of *Theromyzon maculosum* (see Figs. 3g and 4i for such a form from Taymyr), except for the number of eyespots and annuli. If we assume that the eyespots and annulation were poorly recognizable in the heavily contracted holotype, *Placobdella raboti* may be considered a synonym of the latter species, because none of the other leech species in the Scandinavian Arctic shares a more or less similar combination of characters. Here, we would consider *Placobdella raboti* as a *taxon inquirendum* until more convincing evidence of its validity and taxonomic position is presented.

The nominal taxon *Protoclepsis garjaewi* Livanow, 1902 from Lake Baikal^42^ also corresponds morphologically to a melanic form of *Theromyzon maculosum* and may represent its synonym, as it was suggested previously^15^. Conversely, *Theromyzon garjaewi groenlandicum* Bennike, 1939 from West Greenland (type locality: Lake Ferguson, Søndre Strømfjord, 66.9667° N, 50.6333° W)^10,46,47^ may represent a separate Nearctic species of the *maculosum*-group, because the presence of the Palearctic *T. maculosum* in North America was not confirmed^43^. However, the status of this nominal taxon is still uncertain due to the lack of DNA sequence data. There are three more Nearctic species with two annuli between the gonopores, i.e. *T. rude* (Baird, 1869), *T. bifarium* Oosthuizen & Davies, 1993^43^, and *T. tigris* Foote et al., 2022^44^.

The nominal taxon *Protoclepsis meyeri* Livanow, 1902 was described based on syntypes from European Russia (Kazan city, Republic of Tatarstan)^42^. Livanow^42^ stated that ‘‘Unter den *Protoclepsis*-Arten wird *Protoclepsis maculosa* in die Nähe von *Protoclepsis meyeri* zu stellen sein, mit welcher sie in ihrer Organisation durchaus übereinstimmt, so dass beide vielleicht bloss Varietäten ein und derselben Art bilden’’ (p. 358). Based on the original description^42^, *Protoclepsis meyeri* was established on the basis of typical yellow-spotted specimens of *Theromyzon maculosum* and, according to Lukin^15^ and Nesemann and Neubert^10^, should be considered its synonym.

### Subfamily Haementeriinae Autrum, 1939

#### Genus *Helobdella* Blanchard, 1896

Type species: *Helobdella stagnalis* (Linnaeus, 1758) (= *Hirudo stagnalis* Linnaeus, 1758; by original designation).

*Arctic occurrences*. Two allopatric species in this genus were recorded north of the Arctic Circle: *Helobdella stagnalis* and one species new to science, which is described below (Table 1).

*Comments*. The nominal species *Placobdella guernei* Blanchard, 1893 was described from Arctic Norway (type locality: ‘Gadde Luobal, dans le Pasvig, environ par 69° 20′ latitude nord et 27° 30′ longitude est’ = Norway: Lake Gåddeluobbal, Paatsjoki/Pasvik River basin, 69.2583° N, 29.0804° E) on the basis of a small specimen (7 mm long, 3 mm wide) with poorly developed external features^13^. Previously, it was linked to *Placobdella costata*^15^ or to *Helobdella stagnalis*^48^. Based on the original description^13^, we agree with the latter point of view. In particular, Blanchard^13^ noted that the holotype (fixed by monotypy) is a bioculate specimen with smooth (non-papillated) body, having two very wide preocular annuli, a wide annulus with eyespots, and narrower annuli afterwards. This patterns clearly corresponds to the arrangement of annuli and eyespots on the head region of *Helobdella stagnalis* (Supplementary Fig. S2k). Blanchard^13^ did not mention the presence of the dorsal nuchal scute but some aberrant individuals of *Helobdella stagnalis* may lack this feature^15^. Here, we would consider *Placobdella guernei* as a *taxon inquirendum* until a more convincing proof of its validity and taxonomic placement is presented.

### *Helobdella okhotica* Bolotov, Eliseeva, Klass & Kondakov sp. nov

 = *Helobdella stagnalis* Lukin (1976): 102^25^ (identification error).

Figures 4l, 5f, 7d, Supplementary Figs. S2j, S3d, S7, Supplementary Table S2.

LSID: https://zoobank.org/urn:lsid:zoobank.org:act:EBA26356-E4C4-4D51-888E-EAB7516457A8.

*Holotype*. RMBH Hir_0251_1-H (non-sequenced), RUSSIA: Khodeevskoye Lake, 64.7501° N, 177.7771° E, Chukotka Peninsula, July 27, 2019, O. V. Aksenova, A. V. Kondakov & I. V. Vikhrev leg.

*Paratypes* (*N* = 11). RUSSIA: 3 specimens Hir_0251_1 (one sequenced: *COI* sequence acc. No. ON810688; *18S rRNA* sequence acc. No. ON819009), the type locality, the same date, and collectors; 3 specimens RMBH Hir_0003_2 (one sequenced: *COI* sequence acc. No. MN393255), Tumnin River, 49.9451° N, 139.9181° E, Khabarovsk Region, July 14, 2014, I. N. Bolotov & I. V. Vikhrev leg.; 1 specimen RMBH Hir_0294 (sequenced: *COI* sequence acc. No. ON810719), Azabache Lake, 56.1521° N, 161.8561° E, Kamchatka River basin, Kamchatka Peninsula, August 6, 2019, O. V. Aksenova, S. E. Sokolova & A. R. Shevchenko leg.; 3 specimens RMBH Hir_0295 (one sequenced: *COI* sequence acc. No. ON810720), Krasikovskoye Lake, 56.2411° N, 162.0250° E, Kamchatka River basin, Kamchatka Peninsula, August 8, 2019, O. V. Aksenova, S. E. Sokolova & A. R. Shevchenko leg.; 1 specimen RMBH Hir_0491_1 (placed on 34 permanent slides as a series of slices), an unnamed small lake near Kurazhechnoe Lake, 56.3380° N, 160.8479° E, Kamchatka River basin, Kamchatka Peninsula, September 9, 2021, A. V. Kondakov leg.

*Etymology*. The new species is named after the Sea of Okhotsk, because it is widely distributed in freshwater basins, emptying into this sea.

*Differential diagnosis*. Small leech with elongated body; one pair of eyespots; dorsal papillae, tubercles, and dark markings absent; ovate dorsal nuchal scute at VIII a2/a3; one annulus between the male (XIIa1/a2) and female (XIIa2/a3) genital pores. The new species is morphologically similar to *H. stagnalis* and can reliably be distinguished from it by means of the DNA approach only. However, the new species seems to have a smaller dorsal nuchal scute compared with that of *H. stagnalis*. Furthermore, the two species have allopatric ranges and could also be delineated geographically. *Helobdella nuda* (Moore, 1924), which rarely occurs in the Russian Far East and Eastern Siberia^15,49^, differs from the new species by having two pairs of eyespots and by the total lack of dorsal nuchal scute.

*Molecular diagnosis*. Genetically, the new species is most closely related to *H. stagnalis* and *H.* sp. ‘Korea’ (mean uncorrected pairwise *COI p*-distance = 6.9 and 3.8%, respectively). The intraspecific pairwise *COI* p-distance ranges from 0.0 to 2.3% (mean ± s.e.m. = 1.24 ± 0.09%; *N* = 11 sequences and 55 pairwise distance values). The GenBank acc. numbers of reference DNA sequences (*COI* and *18S rRNA*) are given in Supplementary Table S2 and Supplementary Datasets S1-S2.

*Description*. Small leech (body length up to 7.7 mm). Measurements of the type series are given in Supplementary Table S2. Body elongated, with triangular anterior end and ovate dorsal nuchal scute at VIII a2/a3 (furrow between annuli 12 and 13). Dorsum without papillae and tubercles. Posterior sucker circular (maximum diameter of 1.6 mm), ventrally directed, without pigmentation. Proboscis pore in the center of anterior sucker. Coloration of living animals: not examined. Coloration of ethanol-preserved animals: dorsum and venter white, without pigmentation. One pair of circular eyespots. Total number of annuli: 67. Somites I–III uniannulate, IV–V biannulate, VI–XXIII triannulate, XXIV–XXV biannulate, XXVI–XXVII uniannulate. The male and female genital pores are separated by one annulus and are located in the furrows XIIa1/a2 and XIIa2/a3, respectively. Reproductive system: 6 pairs of large, elliptical testisacs arranged intra-segmentally from XIV to XIX; atrium small, spherical, the atrial cornua ovate, elongated, laterally directed; paired ejaculatory ducts short; paired ovisacs massive, with several blind lobes, extending from XII to XVI. Digestive system: proboscis sheath rather narrow, very long, J-shaped distally; esophagus narrow, S-shaped; one pair of diffuse salivary glands; crop with 5 pairs of crop caeca: 1st-4th uniform, 5th pair forms uniform posterior caeca; intestine with 4 pairs of long, simple processes and a bag-like extention after the last pair of processes.

*Distribution*. Eastern Siberia (Lena River basin) and Russian Far East (Chukotka, Kamchatka, Kolyma Highland, Khabarovsk Region, Primorye, and Amur Region).

*Habitats and ecology*. This species was recorded from a wide array of water bodies such as rivers, lakes, reservoirs, and even a water puddle (Supplementary Dataset S2); its feeding behavior and life cycle are unknown.

## Discussion

### Geographic distribution of the glossiphoniid leeches in the Arctic

Our results reveal that members of this family commonly occur in Eurasia north of the Arctic Circle. The most northern samples of leeches in our dataset were collected at 72° N around Khatanga settlement on the Taymyr Peninsula (see Fig. 3a and Supplementary Datasets S2–S3). Four species reach this northern latitude: *Glossiphonia balcanica*, *G. concolor*, *G. mollissima*, and *Helobdella stagnalis*. To the best of our knowledge, these records represent the most northern localities of the glossiphoniid leeches ever discovered. There are records of unspecified Hirudinea in benthic samples from Lake Taymyr at 74–75° N^50^ but they may belong to Piscicolidae and/or Acanthobdellidae, which are characterized by a more high-latitude distribution compared with the Glossiphoniidae^15^. Two species of Piscicolidae were collected on fishes from this lake^51^. Furthermore, a few Erpobdellidae species may also be found in the Arctic areas^15^. The northernmost sample of freshwater molluscs in Eurasia was also collected on the Taymyr Peninsula at 73.5° N^32^.

Our study shows that the Arctic fauna contains 14 species of the Glossiphoniidae. Based on their distribution, we reveal that the Eurasian Arctic embraces the northern margins of the Western and Eastern Palearctic Subregions. The first subregion covers much of the northern edge of the continent from Arctic Scandinavia to the Lena River, while the second subregion extends throughout the Kolyma Highland and Chukotka Peninsula. Earlier, the same biogeographic pattern was discovered using freshwater mussels (Unionidae), pond snails (Lymnaeidae), and planktonic crustaceans (Cladocera) as model groups^52–55^.

The discovery of a species-rich assemblage of the glossiphoniid leeches in the Eurasian Arctic was unexpected. Earlier scholars noted that records of freshwater leeches from the Arctic areas are sporadic^13,14^. Lukin^15^ delineated the Subarctic-Kamchatka Zone, which extends from Arctic Scandinavia to the northeastern edge of Asia (Chukotka and Kamchatka peninsulas). The presence of Acanthobdellidae taxa and *Cystobranchus mamillatus* was noted as a characteristic feature of this biogeographic region. Among the Glossiphoniidae, *Glossiphonia complanata*, *Helobdella stagnalis*, and *Theromyzon tessulatum* were mentioned as occurring there, while records of *G. verrucata* and *T. maculosum* were expected. Sawyer^2^ stated that the leech fauna of the entire Palearctic Region is largely homogeneous, with two minor biogeographic subregions, i.e. boreal subregion (specific taxa: *Acanthobdella peledina*, *Cystobranchus mamillatus*, and *Theromyzon maculosum*) and Ponto-Mediterranean subregion. Our results, however, indicate that the diversity of the Glossiphoniidae in the Arctic was largely underestimated.

None of the leeches we have examined is characterized by a continuous trans-Palearctic distribution, although *Theromyzon tessulatum* may be a possible candidate for such a species, because it is thought to occur in Europe, Siberia, Mongolia, and Kamchatka^15^. However, available records of this species from North Asia were established on the basis of morphological features alone and need to be confirmed using the DNA-based approach. Other widespread trans-Palearctic glossiphoniids such as *Glossiphonia complanata* and *Helobdella stagnalis* are found to be composite taxa, each of which contains two or more biological species having more restricted ranges. Ironically, the Arctic *Glossiphonia* ‘*complanata*’ sensu earlier authors^13–16,22,25^ includes several other species but does not contain *G. complanata* (Linnaeus, 1758) as such (see below). The taxonomic concept of several other taxa such as *Hemiclepsis marginata* (O. F. Müller, 1773) has also been shifted recently from a species having a nearly pan-continental distribution^2,10,15^ to a composite taxon, which contains several species with more restricted ranges^56,57^.

### Cryptic taxonomic diversity of *Glossiphonia* leeches in the Arctic

Our study indicates that *Glossiphonia* is the most species-rich genus of freshwater leeches in the Eurasian Arctic demonstrating considerable cryptic diversity. In the Arctic, the *complanata*-group is represented by two species, i.e., *Glossiphonia balcanica* and *G. taymyrensis* sp. nov. Traditionally, *G. complanata* was thought to represent one of the most common freshwater leech species north of the Arctic Circle, the range of which extends from Iceland and Arctic Scandinavia to the northeastern corner of Asia (Chukotka Peninsula)^13–16,22,25,58^. The identity of this taxon was recently clarified on the basis of DNA sequencing of newly collected topotypes^59^ Based on available sequences of this species (see Supplementary Dataset S1), it occurs in more southern regions of Europe, including Austria, Bosnia and Herzegovina, Croatia, France, Germany, Italy, Montenegro, Slovenia, and the British Isles. The southernmost record comes from Morocco (33.4255° N, 5.2729° W), while the northernmost locality is situated in the Moscow Region of Russia (55.0298° N, 37.9449° E). Multiple occurrences of this species from the Eurasian Arctic were based on misidentified specimens of other species (Ref.^59^ and this study). Furthermore, records of *G. complanata* from the Nearctic Region belong to a separate endemic species, *G. elegans* (Verrill, 1872)^3,60^.

Surprisingly, *G. balcanica*, which was described as endemic to the Balkan Peninsula (Montenegro and Kosovo)^61^, was found to be the most widespread and common glossiphoniid leech in the high-latitude part of Eurasia, being ranged from northern Scandinavia to the Taymyr Peninsula (see Tables 1, 2). The modern concept of this taxon is based on the DNA sequences of topotypes^59^. Published occurrences of *G. complanata* from the Arctic Norway^13^, Finnish Lapland^62^, the Kola Peninsula^13^, and Iceland^16^ most likely belong to this species. Moreover, *G. balcanica* is also recorded from boreal areas of Northern European Russia (Arkhangelsk Region) and from the upper part of the Volga River basin (Moscow Region) based on DNA sequences (Supplementary Dataset S2). These results indicate that the range of *G. balcanica* is much broader than it was thought previously^61^ and that its Balkan population may represent a local southern isolate, which survived in a cryptic glacial refugium. Furthermore, it may belong to a wide group of cold-tolerant Arctic taxa, which were originated in the Mediterranean Region^63^. In Balkans, it inhabits small to medium-sized mountain fast running waters^64^ such as the Toplla Spring, its type locality^61^, representing a mountain cold-water limnocrene with an annual mean water temperature of 13.3 ± 1.4 °C^65^. However, it is also known to occur in Lake Skadar^61^.

*Glossiphonia verrucata*, *G. mollissima*, and *G. arctica* sp. nov. are the Arctic members of the *verrucata*-group. *G. verrucata* is a rather enigmatic species, which was described from a natural lake in Berlin^66^. Later, this species was recorded from the British Isles, Denmark, the Netherlands, Northern European Russia, Norway, Sweden, Poland, and Western and Eastern Siberia, and was considered a ‘boreal relict’^10,15,58,67–70^. Occurrences from France, Italy, and Switzerland are doubtful and need to be checked, as they may belong to *G. nebulosa*^68^. Moreover, the identity of Siberian and European specimens needs to be confirmed in the future, because none of the DNA sequences was generated using samples from Europe^69^.

Based on morphological features, the nominal taxon *Glossiphonia octoserialis* Stschegolew, 1922 (type locality: Russia: an oxbow lake in Guselsk Zaymistche, 51.5943° N, 46.1494° E; Lake Peschanoye on Zelenyi Island, 51.5403° N, 46.1048° E; a lake on Kotluban Island near Chapovka river channel, 51.5918° N, 46.2411° E, outskirts of Saratov City, Volga River basin) most likely belongs to the *verrucata*-group. Earlier, it was considered a synonym of *G. verrucata*^71^ or *G. complanata*^15,67^. However, it may also represent a morphological variety of *G. nebulosa*. The placement of this taxon can be established on the basis of sequenced topotypes, which are currently not available.

*Glossiphonia mollissima* appears to be the single species in this genus which is distributed in the Arctic and Subarctic areas of North Asia east of the Lena River basin from the Kolyma Highlands to Chukotka and Kamchatka peninsulas. Hence, a few published occurrences of *G. complanata* from the Arctic Yakutia and the Chukotka Peninsula^25^ should be linked to this species. Moreover, it is known to occur in northwestern North America on Alaska and Kodiak Island^72,73^. The most southern localitites of this species are situated in the Amur Basin and Primorye. The taxonomic history of *G. mollissima* is remarkable. It was initially discovered by Moore^74^ in freshwater leech samples from the Bering Island (Commander Islands) deposited in the collection of National Museum of Natural History (USNM), Washington, D.C., USA. Moore^74^ identified it as belonging to a species *Clepsine mollissima* Grube, 1871, described from Lake Baikal^75^, and transferred it to the genus *Glossiphonia*. Livanow^42^ stated that Grube’s taxon represents a *Theromyzon* species, *T. mollissimum* (Grube, 1871), and that Moore’s sample from the Bering Island may belong to a new species of *Glossiphonia*. Fifty years later, Moore and Meyer^72^ agreed with Livanow and established an updated concept of this taxon as a separate Beringian subspecies of *G. complanata* (see Table 1), although the nomenclatural issues related to Moore’s species-group name *mollissima* are still unclear and will be discussed elsewhere. Lukin^15,67^ suggested that *G. mollissima* may represent a synonym of *G. verrucata*. In a recent identification guide for the Nearctic Hirudinea, this species is also mentioned as *G. verrucata*^76^. We found, however, that *G. mollissima* is distant from *G. verrucata* both morphologically and phylogenetically.

The *concolor*-group contains two species, occurring in the Arctic: *Glossiphonia concolor* and *G. nebulosa*. The first species is widely distributed in the Arctic Eurasia from the Kanin Peninsula and Malozemelskaya Tundra to the Taymyr Peninsula (see Tables 1, 2). Occurrences of *G. concolor* were already reported from several Arctic regions of Eurasia (Bolshezemelskaya Tundra and Yamal Peninsula)^22,23^ based solely on morphological criteria. Here, we follow the traditional morphology-based concept of this taxon^10^, because the DNA sequences of its topotypes are not available. A series of DNA-based occurrences of an unidentified *Glossiphonia* sp. from Eastern Siberia (Lake Baikal Region)^69^ belong to this species (Supplementary Dataset S2). Previously, *G. concolor* was identified from Eastern Siberia and Mongolia on the basis of morphological information^15,39,77^. The southernmost DNA-based records come from mountain streams and ponds of Iran^78^.

The identity of *Glossiphonia nebulosa* was recently assessed based on DNA sequences of newly collected topotypes^59^. Here, we largely expand knowledge of its range based on new records from the Arctic (Polar Urals to Taymyr), Northern European Russia (Arkhangelsk Region), and the North Caucasus (North Ossetia–Alania). Earlier occurrences come from southern and central Europe and Turkey^10,59^. The range of this species appears to be disjunctive, as it was not recorded in Scandinavia and more southern areas of Siberia^69^ and European Russia. In Serbia and Montenegro, it occurs in cold karstic springs, as *G. balcanica* does^64^.

Unfortunately, the vast majority of published checklists and other faunal papers on Arctic freshwater leeches does not contain morphological descriptions and images of specimens^22,24,31^ and, hence, cannot be used as reliable sources of distribution data for *Glossiphonia* spp.

### Taxonomic diversity and distribution of other glossiphoniid genera in the Arctic

Here, we show that a few *Alboglossiphonia*, *Helobdella*, and *Theromyzon* species could be considered members of the Arctic fauna. Two species in each genus were recorded north of the Arctic Circle. It was proposed that records of *Alboglossiphonia* taxa north of the Arctic Circle may hardly be expected^15^ but Zaloznyj^23^ discovered that *A. heteroclita* commonly occurs in the lower section of the Ob River, Yamal, Western Siberia. These observations may partly correspond to *A. sibirica* sp. nov., owing the presence of its sample from the lower Taz River in our collection (see Supplementary Dataset S2). The high-latitude (subarctic) occurrence from Verkhnekolymsk, Arctic Yakutia^41^ should be linked to the latter species. In summary, *Alboglossiphonia* leeches successfully cross the Arctic Circle only on Yamal Peninsula via the Ob and Taz rivers and, hence, cannot be considered common members of the Arctic fauna.

In contrast, the vicariate species *Helobdella stagnalis* and *H. okhotica* sp. nov. are widespread inhabitants of high-latitude environments of Eurasia. The presence of the first species in the Arctic was established in a series of pioneering works by Blanchard^13^, Wiedemann^14^, and Bruun^16^. In Iceland, it was collected from cold water bodies as well as from hot springs of 28–32 °C^16^. We consider it together with *Glossiphonia balcanica* as the most widespread glossiphoniid species in the Eurasian Arctic, the longitudinal range of which extends from Iceland and Arctic Norway to the Taymyr Peninsula. *H. stagnalis* is also characterized by the broadest latitudinal range among the Arctic glossiphoniids, with the southernmost reliable occurrences from Egypt^79,80^. The records from South Africa^81^ should, however, be considered doubtful as this region is too far from the general (Palearctic) range of *H. stagnalis*. These findings may refer to a separate cryptic species. Earlier records of *H. stagnalis* from the Kolyma Highland and Chukotka Peninsula^25^ should be linked to *H. okhotica* sp. nov., a cryptic scute-bearing species that belongs to the *H. stagnalis* species complex. Previously, several cryptic species from this complex were discovered in North America^82^.

Both *Theromyzon* species recorded from the Arctic are parasites of waterfowl and may disperse with their hosts^15^. *Theromyzon maculosum* is considered a boreal relict species^83^. It was found to be common in a few localities in Arctic Norway^19^, Yamal^24^, and Taymyr. In contrast, its records from Iceland and the Bolshezemelskaya Tundra are sporadic^15–17,20^, while none of samples was collected from the Kanin Peninsula, Kolguev Island, and Malozemelskaya Tundra. The more southern part of its range also seems to be disjunctive. More or less stable populations were discovered in Poland^83^, Sweden^19^, North Kazakhstan^84^, and the upper section of the Volga River basin^15^ and Lake Baikal^42,85^ in Russia. The most southern occurrence was reported from Tajikistan^86^.

*Theromyzon tessulatum* appears to be much more widespread and common species compared with *T. maculosum*, although its records from some regions could be based on misidentified specimens of separate cryptic taxa such as *T. mollissimum*. In the Arctic, it was recorded from Iceland, northern Fennoscandia, and the Bolshezemelskaya Tundra, while its reliable occurrences in the Asian part of the Arctic are lacking (see Table 2 and Supplementary Dataset S2). It is also common in several regions such as Poland^87^, Ireland, England, and Scotland^88^, France^89^, European Russia^15^, and Kazakhstan^84,90^, while its morphology-based occurrences in Canada^43,91,92^ are confirmed on the basis of DNA sequences (see Supplementary Dataset S1). Additional sequences are available from several places in European Russia and Montenegro (see Supplementary Dataset S1). Morphology-based records from Mongolia^15,39^, Lake Baikal^40,42,93^, Kamchatka^15^, Kyrgyzstan^94^, Middle East^95^, and North Africa^96^ may belong to other species and need to be confirmed by means of DNA sequencing. Foote et al.^44^ linked two *Theromyzon* specimens from Alberta to *T. tessulatum* based on morphological features. Based on the *COI* sequence data, the Alberta’s specimens does not correspond to available samples of this species from Europe, including our samples from European Russia and Montenegro and BOLD IDS sequences from Sweden (sample ID: CE36154, CE35352, CE18381, and CE18531), Norway (sample ID: CE28079, CE28081, NIVA_TER_28, and CE32113), and Germany (sample ID: GBOL-07515 and GBOL-08789) (see Dataset S4 for detail). Phylogenetically, European samples of *T. tessulatum* belong to the unnamed clade of Foote et al., which contains sequences identified as “*T. bifarium*” (GenBank acc. No. AY047330), “*T. pallens*” (GenBank acc. No. AF003279), “*T. rude*” (GenBank acc. No. AF003262), and “*T. tessulatum*” (GenBank acc. No. AY047318)^44^. In our opinion, this clade represents Müller’s *T. tessulatum*, which was described from Europe (Denmark). The identity of the two specimens from Alberta is unclear but they may belong to an undescribed cryptic species, morphologically resembling *T. tessulatum*. Indeed, this genus needs a global taxonomic revision based on expanded sequence dataset of the Palearctic and Nearctic species that is well beyond the framework of the present study.

Finally, the discovery of *Hyperboreomyzon polaris* gen. & sp. nov. is of exceptional interest. It is unclear why such a remarkable and morphologically peculiar taxon was not observed by earlier scholars, although its rarity and high-latitude range could be a possible explanation. Both the Kolguev Island and the Putorana Plateau are hard-to-reach areas, and none of freshwater leeches was collected from there before our sampling efforts. At first glance, this taxon may represent a high-latitude relict, as do two Acanthobdellidae species^27,30,31,97^. From an evolutionary point of view, the new genus may be a sister lineage to the Nearctic *Actinobdella*, having a similar sexannulate condition^36^, although external traits such as a specific annulation can arise independently in different clades of the glossiphoniid leeches^3,33^ (see Supplementary Table S3 for detail). Currently, this hypothesis cannot be examined in more detail due to the lack of DNA sequences of *Actinobdella* species. Furthermore, we know almost nothing about the life history and ecology of *Hyperboreomyzon*, and these issues should be considered a high-priority research topic in the future.

### Melanism in Arctic Glossiphoniidae

We found that melanic forms of several species are commonly occur in the Arctic, including those of *Theromyzon maculosum*, *Glossiphonia balcanica*, *G. taymyrensis* sp. nov., *G. mollissima*, and *G. verrucata*. To the best of our knowledge, this discovery is the first evidence of the high-latitude melanism in the subclass Hirudinea. Globally, melanism of leeches is a poorly known phenomenon. Light-colored and darker forms were discovered in several glossiphoniid species such as *Placobdella ali* Huges & Siddall, 2007^98^, *P. parasitica* (Say, 1824)^99^, and *P. rugosa* (Verrill 1874) ^100^. Sawyer^101^ showed that *Placobdella* sp. exhibits the progressive darkening with age, which could be explained by accumulation of metabolic products from digestion of blood. Another example of a melanic coloration was described for a semi-aquatic/aquatic population of the terrestrial leech *Haemopis septagon* Sawyer & Shelley, 1976 (Haemopidae) from North Carolina^102^. In that case, the shift to melanic phenotype was probably driven by adaptation to an aquatic habitat, which is unusual and to some extent extremal for a terrestrial leech^102^.

Some of *Glossiphonia* color forms from the Arctic areas (see Fig. 8f,g,j) clearly resemble *G. complanata maculosa* Sket, 1968, a darker, yellow-spotted subspecies from the Ohrid and Prespa lakes, having a shallow level of genetic divergence from the nominate subspecies of *G. complanata*^10,59^. Our results indicate that such a ‘maculosa’ color form is presented in two more species of the *complanata*-group (*G. balcanica* and *G. taymyrensis* sp. nov.) and that it represents an intermediate melanic phenotype, which frequently occurs in the Arctic. In general, the degree of melanism increases from f. ‘maculosa’ (dark ground color with multiple yellow spots) to ‘darker’ forms (dark ground color with highly reduced markings pattern) (see Fig. 8 for detail).

It was shown that the frequency of melanic individuals in terrestrial and aquatic arthropods increases with latitude and altitude, indicating that selective advantages of melanism are driven by absorption of solar radiation to increase the body temperature and by protection from UV-B radiation^103–105^. The patterns and causes of melanism in Hirudinea are poorly known, although, at first glance, the frequent occurrence of melanic forms in Arctic glossiphoniid leeches discovered by us could be linked to UV-B stress or even to cryptic coloration, because the thermoregulatory function of melanism is less obvious for aquatic environments. It is unclear whether darker leech forms from the Arctic could be linked to the age-dependent progressive darkening (developmental melanism)^101^ or not, because our samples mostly contain adult specimens.

## Methods

### Data sampling

The samples of freshwater leeches were collected by hands and by a hydrobiological net from various water bodies of the Eurasian Arctic and several other regions during the period of 2011–2021. The samples were fixed in 96% ethanol and are deposited in the Russian Museum of Biodiversity Hotspots (RMBH), N. Laverov Federal Center for Integrated Arctic Research of the Ural Branch of the Russian Academy of Sciences (Arkhangelsk, Russia). Information on samples of the glossiphoniid leeches belonging to the Arctic fauna is presented in Supplementary Dataset S2. This dataset contains data on the *COI* and *18S rRNA* gene sequences, specimen voucher, taxonomic status of specimen (type vs non-type materials), geographic position (Arctic vs non-Arctic), region, locality, habitat, geographic coordinates (decimal degrees), collecting date, collectors, and references. Some morphology-based occurrences from published sources were also added to Supplementary Dataset S2, including those from the Arctic localities as well as the most southern records of taxa.

### Morphological and anatomical research

External morphology of the glossiphoniid leeches under discussion was examined based on the body size and shape, annulation, papillation, position of genital pores, ground color, markings pattern, and the number and position of eyespots^2,10,15,81^. The images of specimens and their morphological and anatomical details were taken with stereoscopes Leica M165C (Leica Microsystems GmbH, Germany) and Zeiss Axio Zoom.V16 (Carl Zeiss AG, Germany) and were processed using Adobe Photoshop CS v. 8.0. Body and suckers for the new species were measured using a stereomicroscope Leica M165C (Leica Microsystems GmbH, Germany) equipped with an ocular-micrometer. We obtained four measurements: body length (BL), body width (BW), width of anterior sucker (AW), and width of posterior sucker (PW)^56^. To study the reproductive and digestive systems of larger leeches, specimens were dissected using a standard approach^15^. Furthermore, we prepared a series of longitudinal slices of the body of several target (new to science) taxa. In particular, the leeches were processed using hematoxylin and eosin (H&E) stain^57^ as described below. After fixation, the tissues were dehydrated through a graded alcohol series and embedded in paraffin. Histological sections with a thickness of 6 µm were made using a rotary microtome (HM 325; Thermo Scientific, Waltham, MA, USA). The sections were de-paffinized using the following sequence of solutions: xylene I (15 min) =  > xylene II (10 min) =  > absolute ethanol I (5 min) =  > 96% ethanol (5 min) =  > deionized water rinsing. Then, the slices were placed into Harris hematoxylin staining solution for 2 min, rinsed with deionized water, differentiated in tap water, rinsed with deionized water. After that, the sections were placed into eosin staining solution for 1 min, rinsed with deionized water. Finally, the slices were processed sequentially through the following solutions: absolute ethanol I (1 min) =  > absolute ethanol II (1 min) =  > xylene I (5 min) =  > xylene II (5 min). The permanent slides were prepared using a mounting medium (Vitrogel, Biovitrum, Russia). Histological sections were examined using a stereomicroscope Leica M165C (Leica Microsystems GmbH, Germany). Photos of histological preparations were obtained using the stereomicroscope with a digital camera (FLEXACAM C1, Leica Microsystems, Wetzlar, Switzerland). Photos were processed with Adobe Photoshop CS v. 8.0.

### DNA sequences, species delimitation, and phylogenetic analyses

New sequences of the mitochondrial *cytochrome c oxidase subunit I* (*COI*) and the nuclear *small subunit of 18S ribosomal RNA* (*18S rRNA*) gene sequences were generated using the standard primer pairs and laboratory protocols as described in our earlier work^56^. Forward and reverse sequence reactions were performed directly on purified PCR products using the ABI PRISM^®^ BigDye™ Terminator v. 3.1 reagents kit and run on an ABI PRISM^®^ 3730 DNA analyzer (Thermo Fisher Scientific Inc., Waltham, MA, USA). The new DNA sequences were checked visually using BioEdit v. 7.2.5^106^.

Our *COI* alignment for species delimitation analyses contains 934 in-group sequences of the Glossiphoniidae (209 new and 725 published) and one published outgroup sequence of *Alexandrobdella makhrovi* Bolotov et al., 2020 (Piscicolidae) (Supplementary Dataset S1). Altogether 89 *COI* sequences (76 new and 13 published) in this alignment were collected for samples of the Glossiphoniidae from the Eurasian Arctic (Supplementary Dataset S2). Furthermore, the alignment also includes 182 *COI* sequences (66 new and 116 published), representing non-Arctic samples of the species, belonging to the Arctic fauna (Supplementary Dataset S2). The sequences were aligned directly using MUSCLE algorithm of MEGA7^107^. The 934 in-group *COI* sequences were collapsed to 477 unique haplotypes with FaBox v. 1.61 (https://birc.au.dk/~palle/php/fabox/)^108^. The maximum likelihood phylogeny was generated using the *COI* haplotype alignment through a web-server for IQ-TREE v. 1.6.12 (http://iqtree.cibiv.univie.ac.at) with an automatic identification of the most appropriate evolutionary model based on Bayesian information criterion scores (one partition: GTR + F + R5) and ultra-fast bootstrapping algorithm (1000 replications)^109–111^. This phylogeny was used as an input tree to delimit Molecular Operational Taxonomic Units (MOTUs) using the Poisson Tree Process (PTP) modeling through an online PTP service (http://mptp.h-its.org)^112^. Earlier, the PTP was found to be the most appropriate model to delimit species in the family Glossiphoniidae, because the resulting MOTUs largely agree with the modern taxonomy^56^. Each target MOTU was examined based on morphological and geographical criteria and was compared with the protologues of available nominal taxa to link each phylogenetic species-level clade to a certain taxon^56^. Uncorrected pairwise p-distances between *COI* haplotypes and lineages at the interspecific and intraspecific levels were calculated in MEGA7^107^.

To reconstruct a multi-locus phylogeny of the Hirudinea, we used a combined alignment of the *COI* and *18S rRNA* sequences. One or two haplotypes per species were selected. In total, our dataset contains 141 taxa (Supplementary Table S1). The in-group contains the DNA sequences of the Glossiphoniidae (*N* = 102). The outgroup consists of Erpobdellidae (*N* = 8), Gastrostomobdellidae (*N* = 2), Haemadipsidae (*N* = 2), Haemopidae (*N* = 2), Hirudinidae (*N* = 8), Orobdellidae (*N* = 2), Ozobranchidae (*N* = 2), Piscicolidae (*N* = 6), Salifidae (*N* = 6), and Acanthobdellidae (*N* = 1). *Acanthobdella peledina* (Acanthobdellidae) was used to root the phylogeny. Each gene sequence dataset was separately aligned using the MUSCLE algorithm of MEGA7^107^. The *18S rRNA* gene alignment was additionally processed with GBlocks v. 0.91b through an online server^113,114^ to exclude large gaps and hypervariable positions (final length of 1697 bp; 83% of the original 2031 bp). The single gene alignments were joined to a combined alignment using FaBox v. 1.61 (https://birc.au.dk/~palle/php/fabox/)^108^. The maximum likelihood phylogeny (four partitions: 3 codons of *COI* and *18S rRNA*) was generated using a web-server for IQ-TREE v. 1.6.12 (http://iqtree.cibiv.univie.ac.at)^109–111^. The most appropriate models were automatically selected for each partition based on Bayesian information criterion scores^111^ as follows: 1st codon of *COI*: GTR + F + I + G4; 2nd codon of *COI*: GTR + F + G4; 3rd codon of *COI*: TN + F + ASC + G4; and *18S rRNA*: SYM + G4. Node support values were identified using an ultra-fast bootstrap with 5000 replications^110^.

### Statistical analyses

To delineate biogeographic units, we applied a cluster analysis in PAST v. 3.04^115^ using a matrix of Hamming distances and unweighted pair-group average (UPGMA) method of clustering based on the presence-absence dataset of glossiphoniid leeches from subregions of the Eurasian Arctic (Table 2 and Supplementary Dataset S3). We choose the Hamming distance because it is one of the most reliable binary feature vector similarity measures, having a high effectiveness for the species presence-absence data^116–118^. The Mann–Whitney test was calculated using PAST v. 3.04^115^. The map of species richness was created using ESRI ArcGIS 10 software (www.esri.com/arcgis).

### Nomenclatural acts

The electronic edition of this article conforms to the requirements of the amended International Code of Zoological Nomenclature (ICZN), and hence the new names contained herein are available under that Code from the electronic edition of this article. This published work and the nomenclatural acts it contains have been registered in ZooBank (https://zoobank.org/), the online registration system for the ICZN. The LSID for this publication is: https://zoobank.org/urn:lsid:zoobank.org:pub:65B634DA-10A5-4CE9-82AC-074D07100B21. The electronic edition of this paper was published in a journal with an ISSN, and has been archived and is available from PubMed Central.

## Supplementary Information

Supplementary Information 1.

Supplementary Information 2.

Supplementary Information 3.

Supplementary Information 4.

Supplementary Information 5.

Supplementary Information 6.

## Data Availability

The type series of the five new species and non-type materials are available in the Russian Museum of Biodiversity Hotspots (RMBH), N. Laverov Federal Center for Integrated Arctic Research of the Ural Branch of the Russian Academy of Sciences (Arkhangelsk, Russia). The new *COI* and *18S rRNA* gene sequences generated in this study are deposited in GenBank. The GenBank accession numbers of new DNA sequences generated and used in this study are presented in Supplementary Datasets S1–S2 and Supplementary Tables S1–S2. The sequence alignment, partition txt file, and output IQ-TREE tree file for our two-locus phylogeny (*COI* + *18S rRNA*) are submitted as Supplementary Dataset S5 (ZIP archive file). Other raw data (e.g., primary images, additional alignments, etc.) are available upon reasonable request to the corresponding author.
