# Supplementary material for: Cryptic taxonomic diversity and high-latitude melanism in the glossiphoniid leech assemblage from the Eurasian Arctic
Source: Sci Rep. 2022 Nov 30;12:20630. doi: 10.1038/s41598-022-24989-7 (PMC9712395; doi:10.1038/s41598-022-24989-7)
Supplement: Supplementary file 2 — Supplementary Information 2. [file 41598_2022_24989_MOESM2_ESM.pdf]

SUPPLEMENTARY INFORMATION

Cryptic taxonomic diversity and high-latitude melanism in the glossiphoniid leech assemblage from the Eurasian Arctic

Ivan N. Bolotov et al.

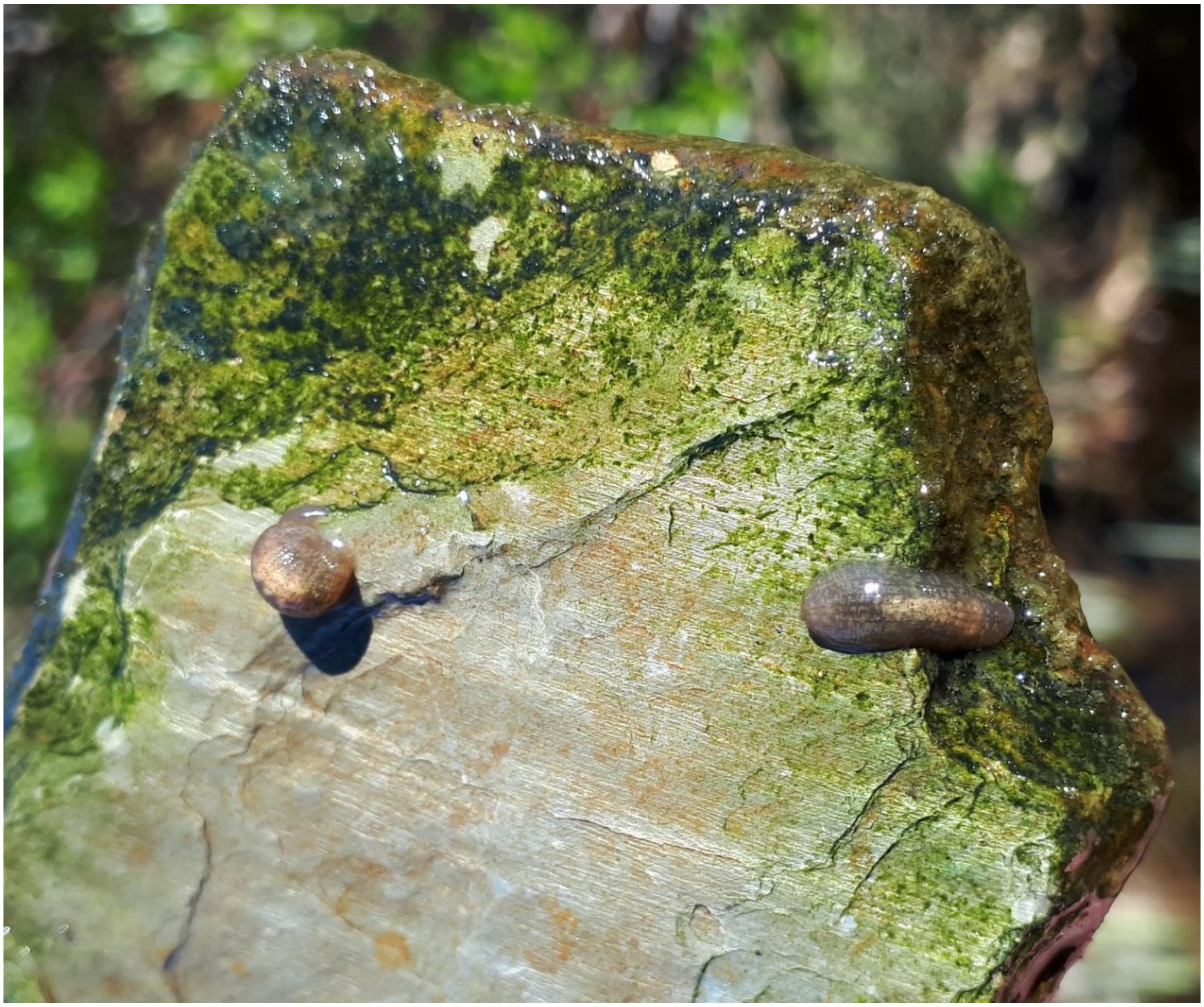

Two adult individuals of *Glossiphonia arctica* **sp. nov.** (part of the type series RMBH Hir_0457) on a flat stone, alpine lake, Polar Urals, Russia, June 23, 2021. Photo: Alexander V. Kondakov.

Contents

Supplementary Figures

Figure S1. Results of the Poisson Tree Process (PTP) species delimitation analysis based on the *COI* haplotype phylogeny of the Glossiphoniidae (*N* = 477 in-group haplotypes) inferred from IQ-TREE v. 1.6.12.

Figure S2. Anterior region with eyespots (dorsal view) of Arctic Glossiphoniidae species. (**a**) *Alboglossiphonia sibirica* **sp. nov.** (holotype RMBH Hir_0542_2-H, Primorye). (**b**) *Glossiphonia arctica* **sp. nov.** (holotype RMBH Hir_0457_2_1-H, Polar Urals). (**c**) *G. balcanica* (light-colored form; specimen RMBH Hir_0249, Kolguev Island). (**d**) *G. concolor* (specimen RMBH Hir_0255, Taymyr). (**e**) *G. mollissima* (melanic form; specimen RMBH Hir_0252, Chukotka Peninsula). (**f**) *G. nebulosa* (specimen RMBH Hir_0081_2, Polar Urals). (**g**) *G. verrucata* (melanic form; specimen RMBH Hir_0605_1, Putorana Plateau). (**h**) *G. taymyrensis* **sp. nov.** (melanic form; holotype RMBH Hir_0258_1-H, Taymyr). (**i**) *Hyperboreomyzon polaris* **gen. & sp. nov.** (paratype RMBH Hir_0216, Kolguev Island). (**j**) *Helobdella okhotica* **sp. nov.** (paratype RMBH Hir_0294, Kamchatka). (**k**) *H. stagnalis* (specimen RMBH Hir_0269, Taymyr). (**l**) *Theromyzon maculosum* (melanic form; specimen RMBH Hir_0263_3, Taymyr). Abbreviations: *ES*, eyespots. Scale bars = 0.5 mm. Photos: Anna L. Klass and Tatyana A. Eliseeva; graphics: Ivan N. Bolotov.

Figure S3. Gonopores (ventral view) of the new Glossiphoniidae species. (**a**) *Alboglossiphonia sibirica* **sp. nov.** (holotype RMBH Hir_0542_2-H, Primorye). (**b**) *Glossiphonia arctica* **sp. nov.** (holotype RMBH Hir_0457_2_1-H, Polar Urals). (**c**) *G. taymyrensis* **sp. nov.** (paratype RMBH Hir_0256_1, Taymyr Peninsula). (**d**) *Helobdella okhotica* **sp. nov.** (holotype Hir_251_1-H, Chukotka Peninsula). Abbreviations: *mg*, male gonopore; and *fg*, female gonopore. Body somites are indicated by roman numerals. Scale bars = 0.5 mm. Photos: Tatyana A. Eliseeva; graphics: Ivan N. Bolotov.

Figure S4. H&E stained longitudinal slices showing the digestive and generative systems of *Alboglossiphonia sibirica* **sp. nov.** (paratype RMBH Hir_0013, Yakutia Republic): (**a**) closer to the ventral side (slide 1-Hir_0013); and (**b**) closer to the dorsal side (slide 5-Hir_0013). Abbreviations: *MO*, mouth; *PR*, proboscis sheath; *EP*, esophagus; *SG*, salivary glands; *CC1-CC5*, 1st–5th pairs of crop caeca; *PC*, 6th pair of crop caeca (posterior caeca); *IS*, intestine; *AN*, anus; *AT*, atrium; *ac*, atrial cornua; *ED*, ejaculatory ducts; *TS1-TS6*, 1st–6th pairs of testisacs; and *OS*, ovisacs. Body somites are indicated by roman numerals. Scale bar = 1.0 mm. Photo: Alexander V. Kropotin; graphics: Ivan N. Bolotov.

Figure S5. H&E stained longitudinal slices showing the digestive and generative systems of *Glossiphonia arctica* **sp. nov.** (pregnant specimen with numerous eggs; paratype RMBH Hir_0457, Polar Urals): (**a**) closer to the ventral side (slide 5-Hir_0457); and (**b**) closer to the dorsal side (slide 12-Hir_0457). Abbreviations: *MO*, mouth; *PR*, proboscis sheath; *EP*, esophagus; *SG*, salivary glands; *CC1-CC6*, 1st–6th pairs of crop caeca; *PC*, 7th pair of crop caeca (posterior caeca); *IS*, intestine; *AN*, anus; *AT*, atrium; *ac*, atrial cornua; *ED*, ejaculatory ducts; *TS*, testisacs; *OS*, ovisacs; *EG*, eggs; and *PS*, posterior sucker. Body somites are indicated by roman numerals. Scale bar = 1.0 mm. Photo: Alexander V. Kropotin; graphics: Ivan N. Bolotov.

Figure S6. H&E stained longitudinal slices showing the digestive and generative systems of *Glossiphonia taymyrensis* **sp. nov.** (strongly contracted specimen; paratype RMBH Hir_0256_1, Taymyr Peninsula) : (**a**) closer to the ventral side (slide 1-Hir_0256_1); and (**b**) closer to the dorsal side (slide 6-Hir_0256_1). Abbreviations: *MO*, mouth; *PR*, proboscis sheath; *EP*, esophagus; *SG*, salivary glands; *CC1-CC5*, 1st–5th pairs of crop caeca (5th pair contracted and folded under the crop); *PC*, 6th pair of crop caeca (posterior caeca; contracted and folded under the crop); *IS*, intestine; *AT*, atrium with atrial cornua; *ED*, ejaculatory ducts; *TS1-TS6*, 1st–6th pairs of testisacs; and *OS*, ovisacs. Body somites are indicated by roman numerals. Scale bar = 1.0 mm. Photo: Alexander V. Kropotin; graphics: Ivan N. Bolotov.

Figure S7. H&E stained longitudinal slices showing the digestive and generative systems of *Helobdella okhotica* **sp. nov.** (paratype RMBH Hir_0491_1, Kamchatka Peninsula): (**a**) closer to the ventral side (slide 1-Hir_0491_1); and (**b**) closer to the dorsal side (slide 2-Hir_0491_1). Abbreviations: *MO*, mouth; *PR*, proboscis sheath; *EP*, esophagus; *SG*, salivary glands; *CC1-CC4*, 1st–4th pairs of crop caeca; *PC*, 5th pair of crop caeca (posterior caeca); *IS*, intestine; *AT*, atrium with atrial cornua; *ED*, ejaculatory ducts; *TS1-TS6*, 1st–6th pairs of testisacs; and *OS*, ovisacs. Body somites are indicated by roman numerals. Scale bar = 1.0 mm. Photo: Alexander V. Kropotin; graphics: Ivan N. Bolotov.

Figure S8. Holotype of *Hyperboreomyzon polaris* **gen.** & **sp. nov.** (RMBH Hir_0486-H, Putorana Plateau): external features and annulation in dorsal (*D*) and ventral (*V*) view. Abbreviations: *ES*, eyespots; *MO*, mouth; *mg*, male gonopore; *fg*, female gonopore; and *PS*, posterior sucker. Body somites are indicated by roman numerals. Scale bar = 1.0 mm. Photos: Tatyana A. Eliseeva; graphics: Ivan N. Bolotov.

Figure S9. Anterior sucker of *Hyperboreomyzon polaris* **gen.** & **sp. nov.** (paratype RMBH Hir_0216, Kolguev Island) (ventral view). Abbreviations: *PP*, proboscis pore, *TF*, thickened fold inside anterior sucker. Body somites are indicated by roman numerals. Scale bar = 0.5 mm. Photo: Tatyana A. Eliseeva; graphics: Ivan N. Bolotov.

Figure S10. Dorsal (D) and ventral (V) views of two paratypes of *Hyperboreomyzon polaris* **gen.** & **sp. nov.** (**a**) paratype RMBH Hir_0216, Kolguev Island; and (**b**) paratype RMBH Hir_0689, Putorana Plateau. Scale bar = 1.0 mm. Photos: Anna L. Klass (a) and Tatyana A. Eliseeva (b).

Figure S11. H&E stained longitudinal slices showing the digestive and generative systems of *Hyperboreomyzon polaris* **gen.** & **sp. nov.** (paratype RMBH Hir_0689, Putorana Plateau): (**a**) closer to the ventral side (slide 24-Hir_0689); and (**b**) closer to the dorsal side (slide 30-Hir_0689). Abbreviations: *MO*, mouth; *PR*, proboscis sheath; *SG*, salivary glands; *CC1-CC8*, 1st–8th pairs of crop caeca; *PC*, 9th pair of crop caeca (posterior caeca); *IS*, intestine; *bt*, black tissue covering atrium; *ED*, ejaculatory ducts; *TS1-TS6*, 1st–6th pairs of testisacs; and *PS*, posterior sucker. Body somites are indicated by roman numerals. Scale bar = 2.0 mm. Photo: Alexander V. Kropotin; graphics: Ivan N. Bolotov.

Figure S12. Maps of occurrences of the Glossiphoniidae species recorded from the Arctic (species with Palearctic ranges). (**a**) *Glossiphonia balcanica*; (**b**) *G. concolor*; (**c**) *G. nebulosa*; (**d**) *G. verrucata*; (**e**) *Helobdella stagnalis*; and (**f**) *Theromyzon maculosum*. The red circles indicate original records; the blue circles indicate published records; the blue star indicate the type locality of *G. verrucata* (see Dataset

2 for raw occurrence data and literature sources). The map was created using ESRI ArcGIS 10 software (www.esri.com/arcgis).

Figure S13. Maps of occurrences of the Glossiphoniidae species recorded from the Arctic (species with Holarctic ranges). (**a**) *Glossiphonia mollissima*; and (**b**) *Theromyzon tessulatum*. The red circles indicate original records; the blue circles indicate published records (see Dataset 2 for raw occurrence data and literature sources). The map was created using ESRI ArcGIS 10 software (www.esri.com/arcgis).

Figure S14. Box plot of uncorrected *COI* p-distances (%) between *Hyperboreomyzon polaris* **gen. & sp. nov.** and other genera of the Glossiphoniidae. The distances were calculated between a haplotype of *H. polaris* and selected species-level haplotypes in each genus (Table S1).

Supplementary Tables

Table S1. Information on the *COI* and *18S rRNA* gene sequences used in a two-locus phylogenetic reconstruction of the Hirudinea (accession numbers of the new sequences generated in this study are bold; other sequences were obtained from GenBank).

Table S2. Voucher numbers, accession numbers of reference DNA sequences (the numbers of newly generated sequences are bold), and measurements for the type series of the new taxa.

Table S3. Morphological and anatomical characters of Glossiphoniidae genera.

Supplementary Datasets (separate files)

Dataset S1. Information on *COI* sequences of Hirudinida taxa used in the species delimitation modeling. The GanBank accession numbers of the new sequences generated in this study are bold (XLSX file).

Dataset S2. Occurrences and information on DNA sequences of freshwater leeches (Glossiphoniidae), belonging to the Arctic fauna (XLSX file).

Dataset S3. Presence-absence distribution data on Arctic freshwater leeches (Glossiphoniidae). The violet filling (1) indicates DNA-based data; and the yellow filling (2) indicates published and original records based on morphology (XLSX file).

Dataset S4. The *COI* sequence-based identification of *Theromyzon tessulatum* (O. F. Müller, 1773) using the Barcoding of Life Database (BOLD IDS) (PDF file).

Dataset S5. The combined *COI + 18S rRNA* fasta alignment, IQ-TREE tree file, and partition txt file (taxonomic names abbreviations correspond to those in Table S1) (ZIP archive file).

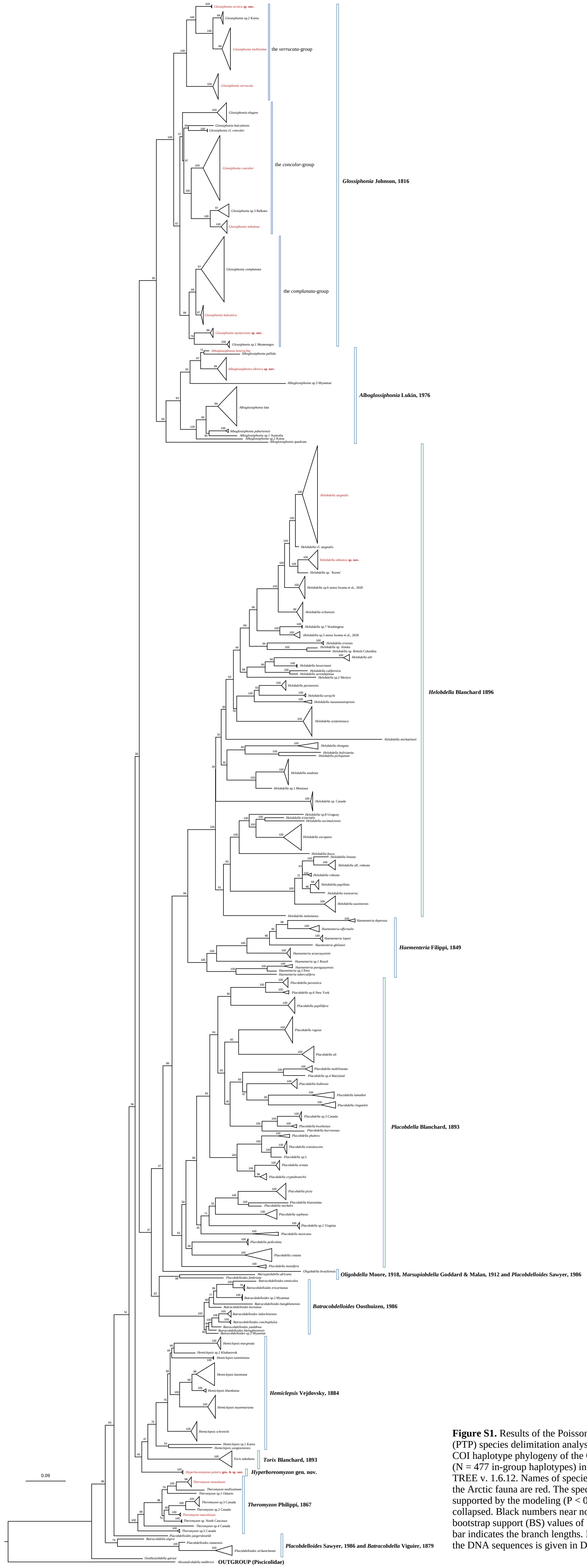

Figure S1. Results of the Poisson Tree Process (PTP) species delimitation analysis based on the COI haplotype phylogeny of the Glossiphoniidae (N = 477 in-group haplotypes) inferred from IQ-TREE v. 1.6.12. Names of species presented in the Arctic fauna are red. The species-level clades supported by the modeling ($P < 0.001$) are collapsed. Black numbers near nodes are bootstrap support (BS) values of IQ-TREE. Scale bar indicates the branch lengths. Information on the DNA sequences is given in Dataset S1.

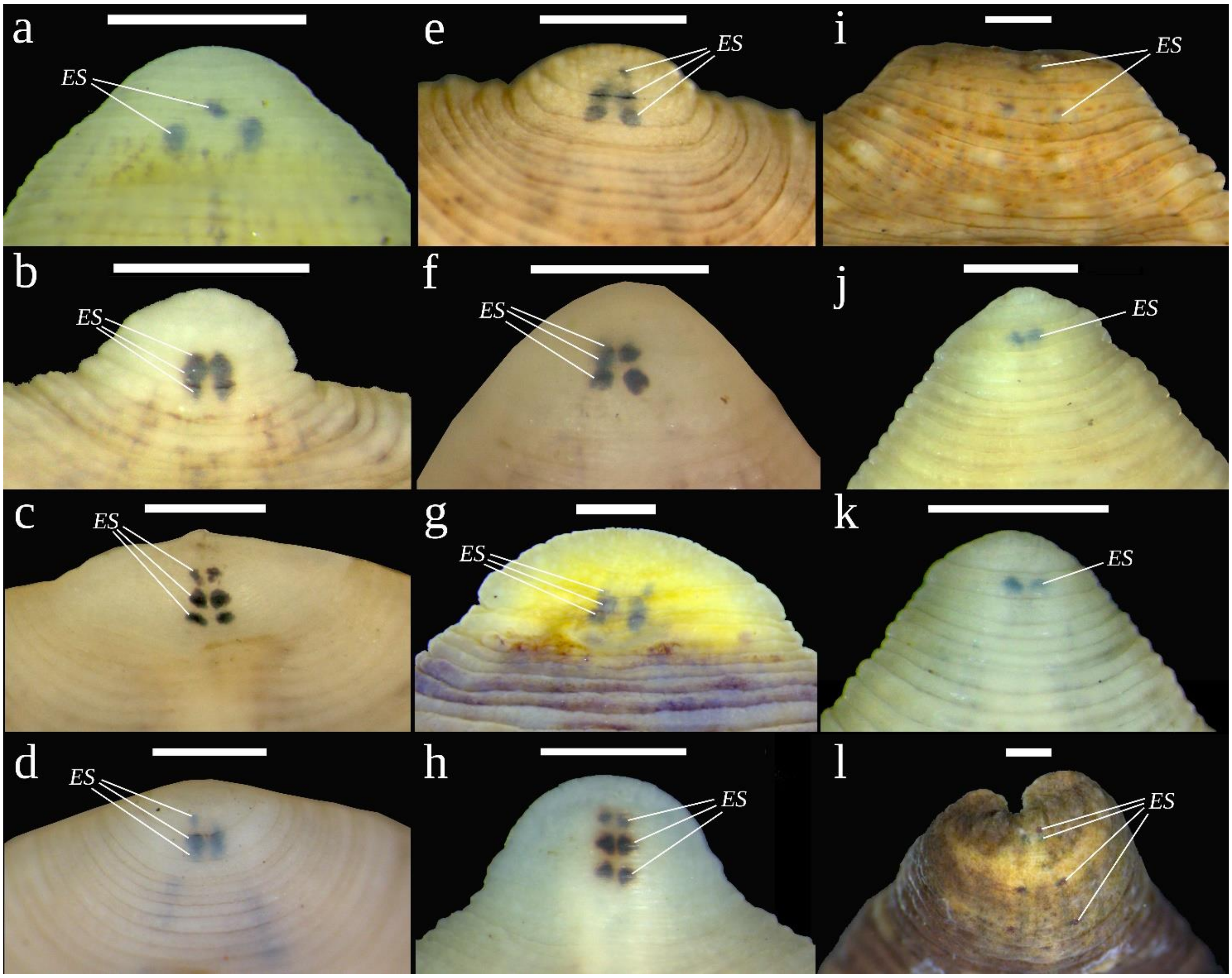

Figure S2. Anterior region with eyespots (dorsal view) of Arctic Glossiphoniidae species. (**a**) *Alboglossiphonia sibirica* **sp. nov.** (holotype RMBH Hir_0542_2-H, Primorye). (**b**) *Glossiphonia arctica* **sp. nov.** (holotype RMBH Hir_0457_2_1-H, Polar Urals). (**c**) *G. balcanica* (light-colored form; specimen RMBH Hir_0249, Kolguev Island). (**d**) *G. concolor* (specimen RMBH Hir_0255, Taymyr). (**e**) *G. mollissima* (melanic form; specimen RMBH Hir_0252, Chukotka Peninsula). (**f**) *G. nebulosa* (specimen RMBH Hir_0081_2, Polar Urals). (**g**) *G. verrucata* (melanic form; specimen RMBH Hir_0605_1, Putorana Plateau). (**h**) *G. taymyrensis* **sp. nov.** (melanic form; holotype RMBH Hir_0258_1-H, Taymyr). (**i**) *Hyperboreomyzon polaris* **gen. & sp. nov.** (paratype RMBH Hir_0216, Kolguev Island). (**j**) *Helobdella okhotica* **sp. nov.** (paratype RMBH Hir_0294, Kamchatka). (**k**) *H. stagnalis* (specimen RMBH Hir_0269, Taymyr). (**l**) *Theromyzon maculosum* (melanic form; specimen RMBH Hir_0263_3, Taymyr). Abbreviations: *ES*, eyespots. Scale bars = 0.5 mm. Photos: Anna L. Klass and Tatyana A. Eliseeva; graphics: Ivan N. Bolotov.

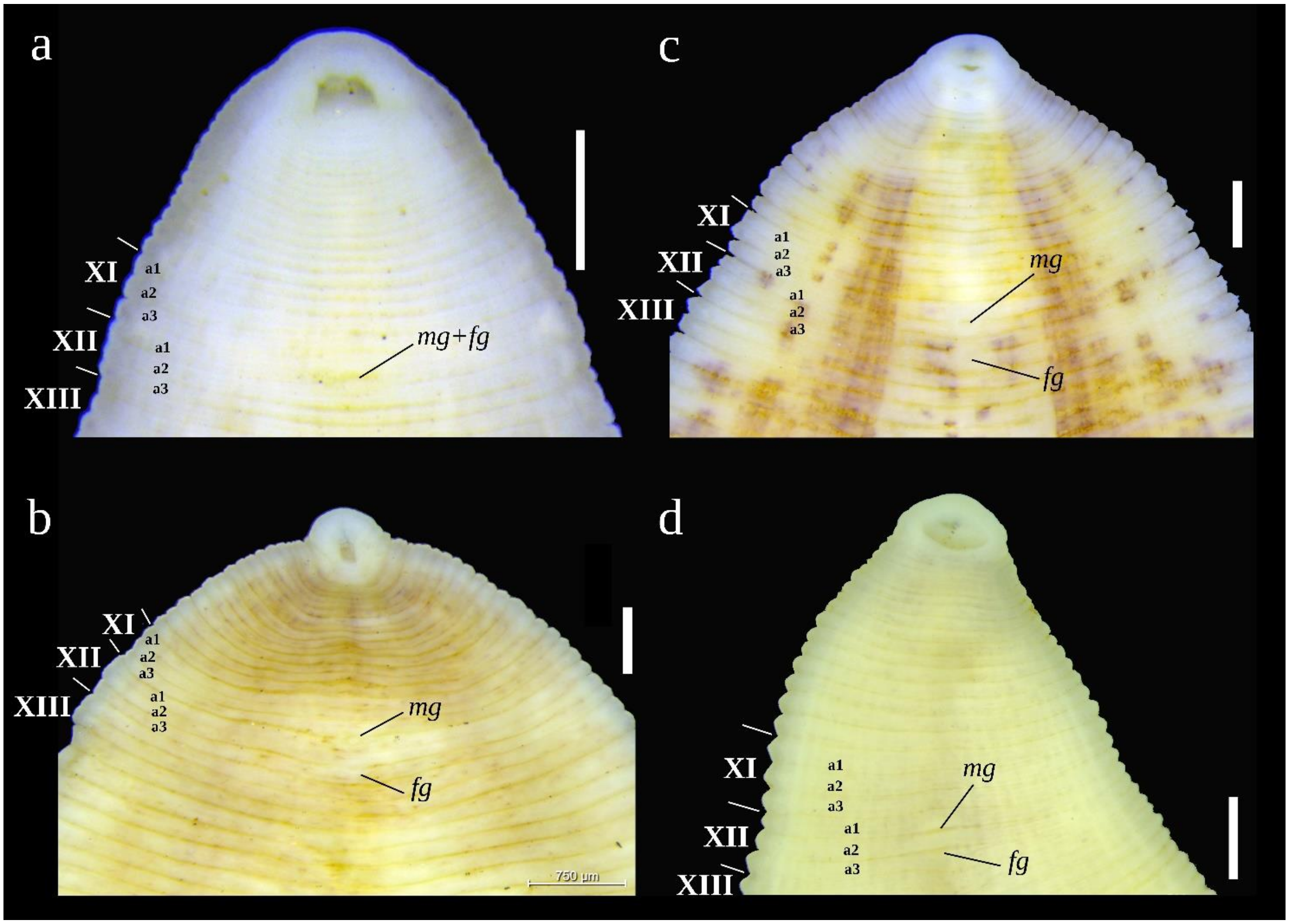

Figure S3. Gonopores (ventral view) of the new Glossiphoniidae species. (**a**) *Alboglossiphonia sibirica* **sp. nov.** (holotype RMBH Hir_0542_2-H, Primorye). (**b**) *Glossiphonia arctica* **sp. nov.** (holotype RMBH Hir_0457_2_1-H, Polar Urals). (**c**) *G. taymyrensis* **sp. nov.** (paratype RMBH Hir_0256_1, Taymyr Peninsula). (**d**) *Helobdella okhotica* **sp. nov.** (holotype Hir_251_1-H, Chukotka Peninsula). Abbreviations: *mg*, male gonopore; and *fg*, female gonopore. Body somites are indicated by roman numerals. Scale bars = 0.5 mm. Photos: Tatyana A. Eliseeva; graphics: Ivan N. Bolotov.

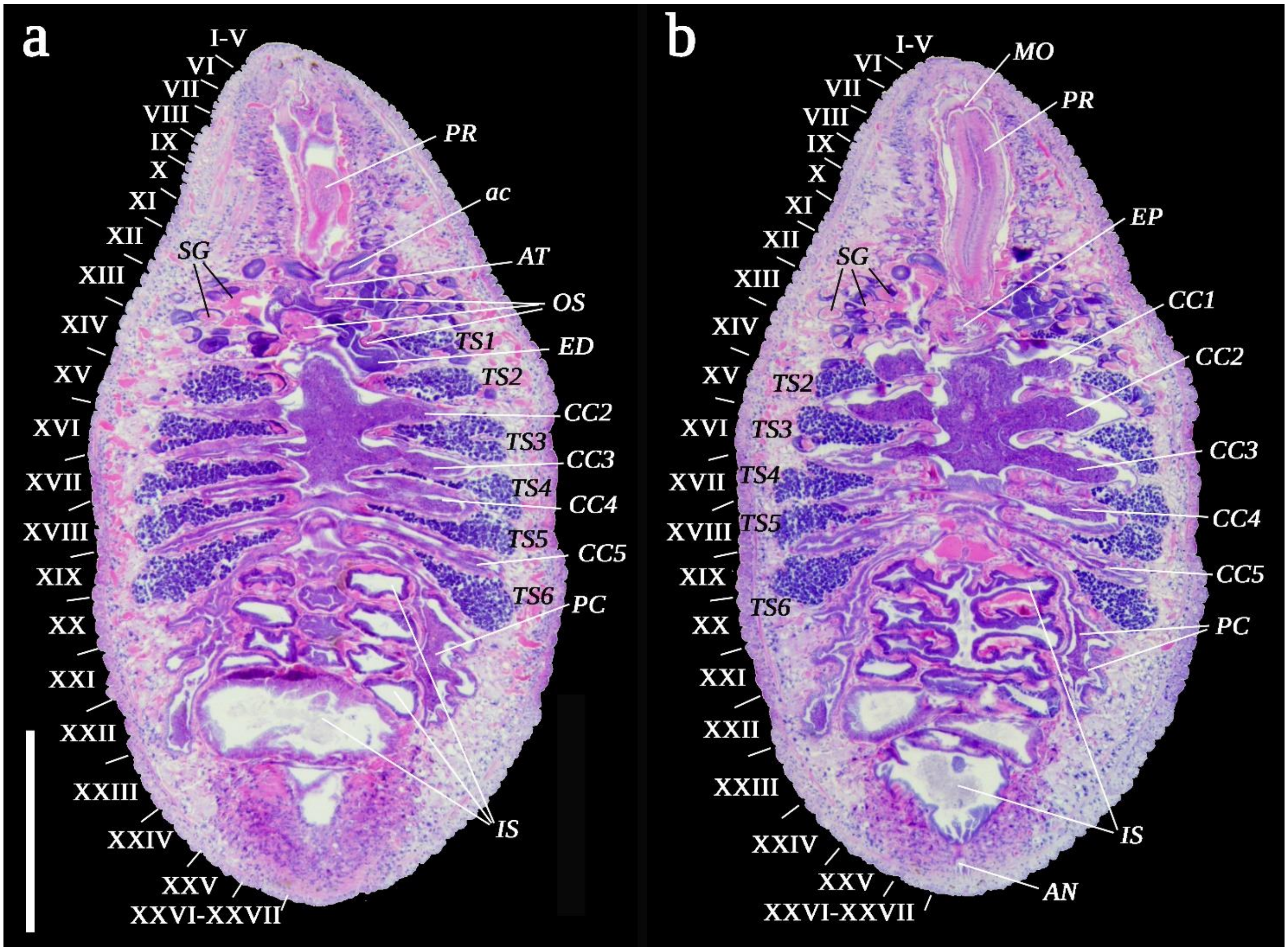

Figure S4. H&E stained longitudinal slices showing the digestive and generative systems of *Alboglossiphonia sibirica* **sp. nov.** (paratype RMBH Hir_0013, Yakutia Republic): (**a**) closer to the ventral side (slide 1-Hir_0013); and (**b**) closer to the dorsal side (slide 5-Hir_0013). Abbreviations: *MO*, mouth; *PR*, proboscis sheath; *EP*, esophagus; *SG*, salivary glands; *CC1-CC5*, 1st–5th pairs of crop caeca; *PC*, 6th pair of crop caeca (posterior caeca); *IS*, intestine; *AN*, anus; *AT*, atrium; *ac*, atrial cornua; *ED*, ejaculatory ducts; *TS1-TS6*, 1st–6th pairs of testisacs; and *OS*, ovisacs. Body somites are indicated by roman numerals. Scale bar = 1.0 mm. Photo: Alexander V. Kropotin; graphics: Ivan N. Bolotov.

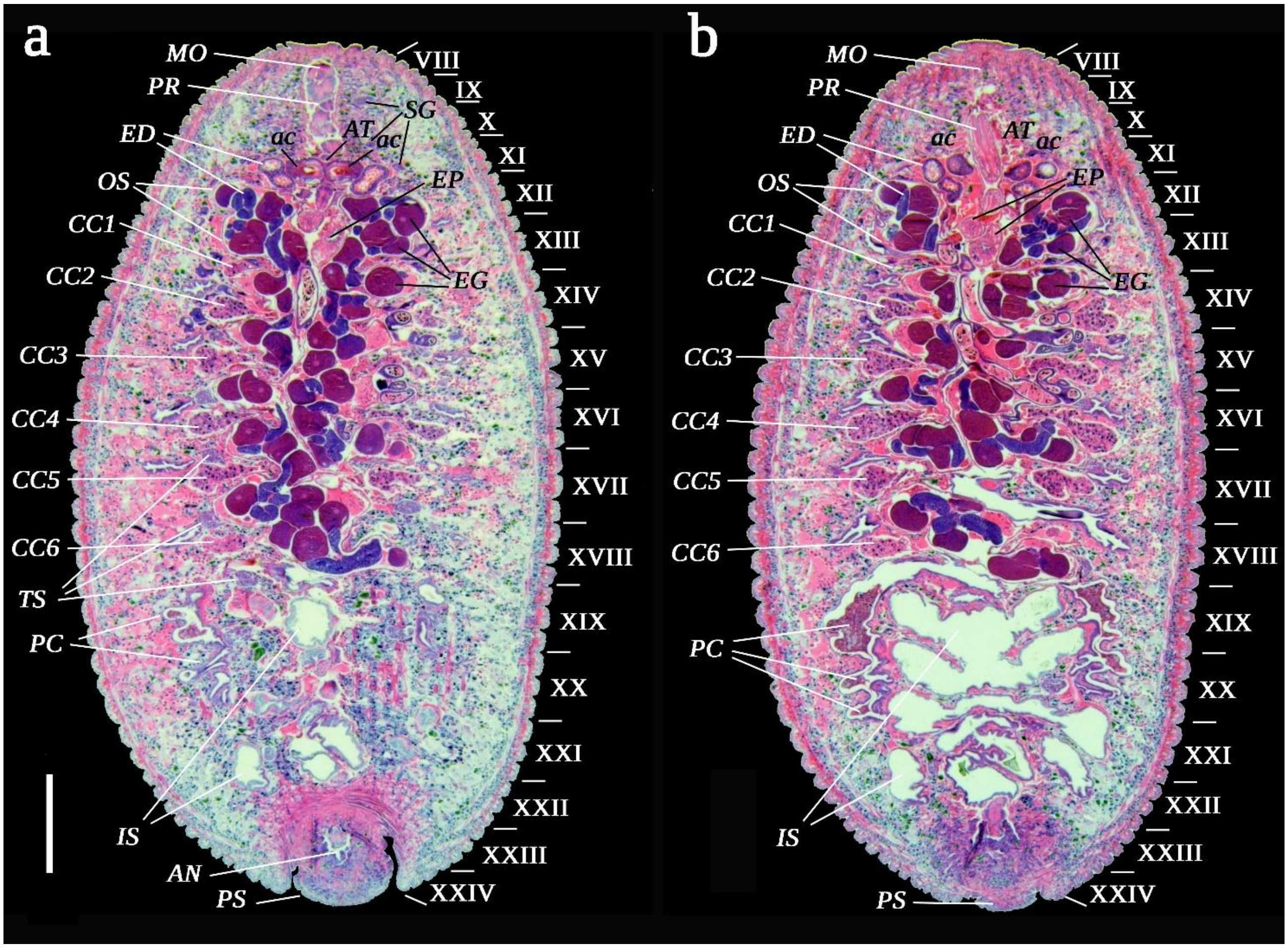

Figure S5. H&E stained longitudinal slices showing the digestive and generative systems of *Glossiphonia arctica* **sp. nov.** (pregnant specimen with numerous eggs; paratype RMBH Hir_0457, Polar Urals): (**a**) closer to the ventral side (slide 5-Hir_0457); and (**b**) closer to the dorsal side (slide 12-Hir_0457). Abbreviations: *MO*, mouth; *PR*, proboscis sheath; *EP*, esophagus; *SG*, salivary glands; *CC1-CC6*, 1st–6th pairs of crop caeca; *PC*, 7th pair of crop caeca (posterior caeca); *IS*, intestine; *AN*, anus; *AT*, atrium; *ac*, atrial cornua; *ED*, ejaculatory ducts; *TS*, testisacs; *OS*, ovisacs; *EG*, eggs; and *PS*, posterior sucker. Body somites are indicated by roman numerals. Scale bar = 1.0 mm. Photo: Alexander V. Kropotin; graphics: Ivan N. Bolotov.

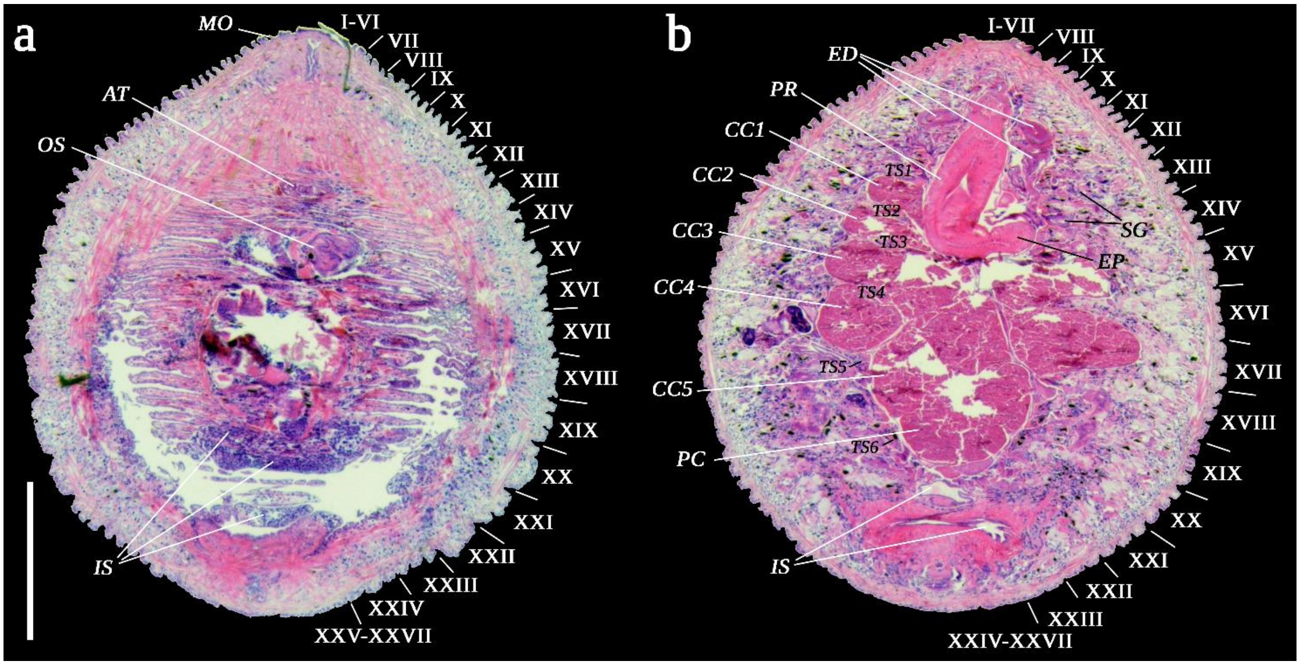

Figure S6. H&E stained longitudinal slices showing the digestive and generative systems of *Glossiphonia taymyrensis* **sp. nov.** (strongly contracted specimen; paratype RMBH Hir_0256_1, Taymyr Peninsula) : (**a**) closer to the ventral side (slide 1-Hir_0256_1); and (**b**) closer to the dorsal side (slide 6-Hir_0256_1). Abbreviations: *MO*, mouth; *PR*, proboscis sheath; *EP*, esophagus; *SG*, salivary glands; *CC1-CC5,* 1st–5th pairs of crop caeca (5th pair contracted and folded under the crop); *PC*, 6th pair of crop caeca (posterior caeca; contracted and folded under the crop); *IS*, intestine; *AT*, atrium with atrial cornua; *ED*, ejaculatory ducts; *TS1-TS6*, 1st–6th pairs of testisacs; and *OS*, ovisacs. Body somites are indicated by roman numerals. Scale bar = 1.0 mm. Photo: Alexander V. Kropotin; graphics: Ivan N. Bolotov.

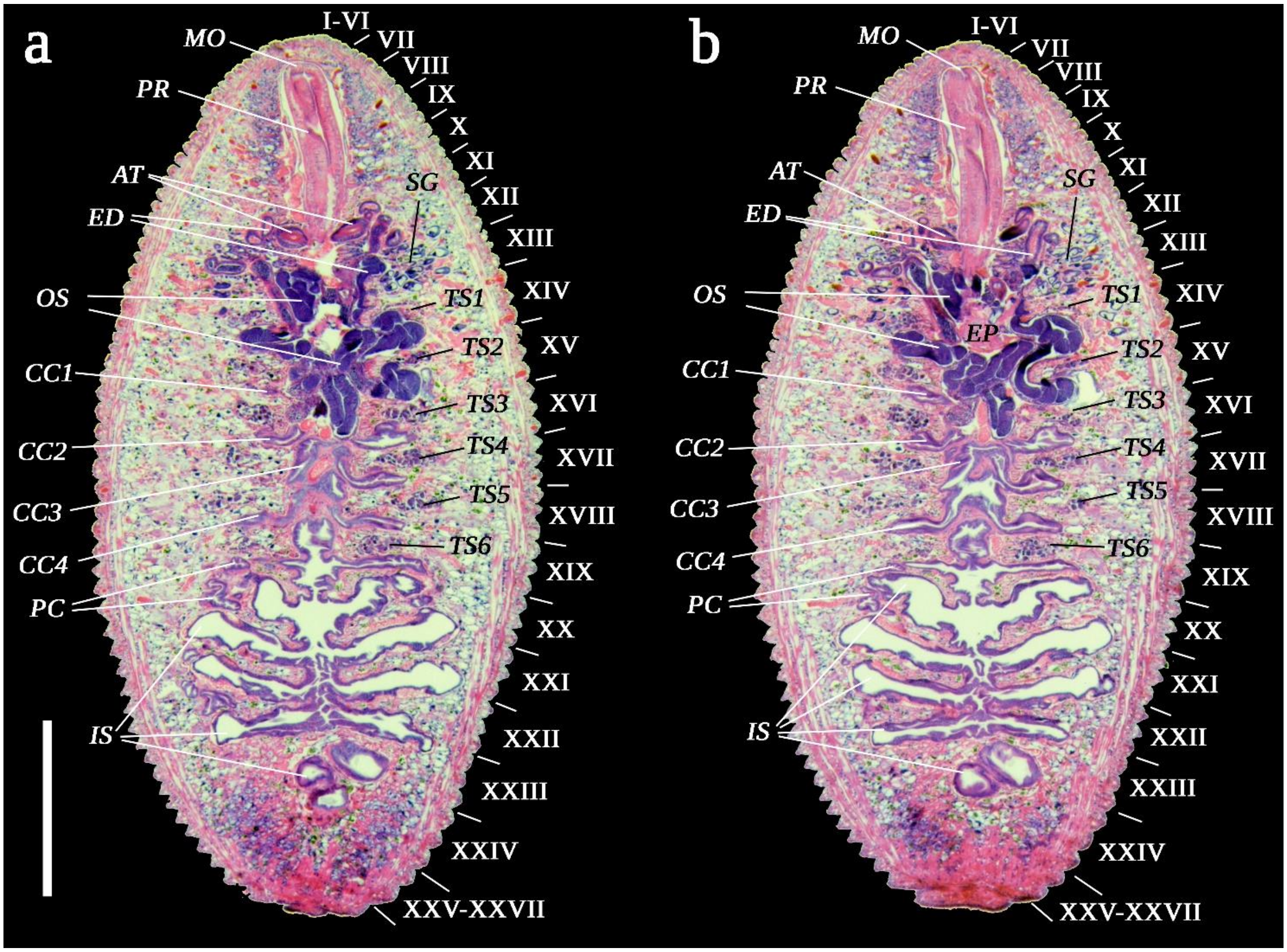

Figure S7. H&E stained longitudinal slices showing the digestive and generative systems of *Helobdella okhotica* **sp. nov.** (paratype RMBH Hir_0491_1, Kamchatka Peninsula): (**a**) closer to the ventral side (slide 1-Hir_0491_1); and (**b**) closer to the dorsal side (slide 2-Hir_0491_1). Abbreviations: *MO*, mouth; *PR*, proboscis sheath; *EP*, esophagus; *SG*, salivary glands; *CC1-CC4*, 1st–4th pairs of crop caeca; *PC*, 5th pair of crop caeca (posterior caeca); *IS*, intestine; *AT*, atrium with atrial cornua; *ED*, ejaculatory ducts; *TS1-TS6*, 1st–6th pairs of testisacs; and *OS*, ovisacs. Body somites are indicated by roman numerals. Scale bar = 1.0 mm. Photo: Alexander V. Kropotin; graphics: Ivan N. Bolotov.

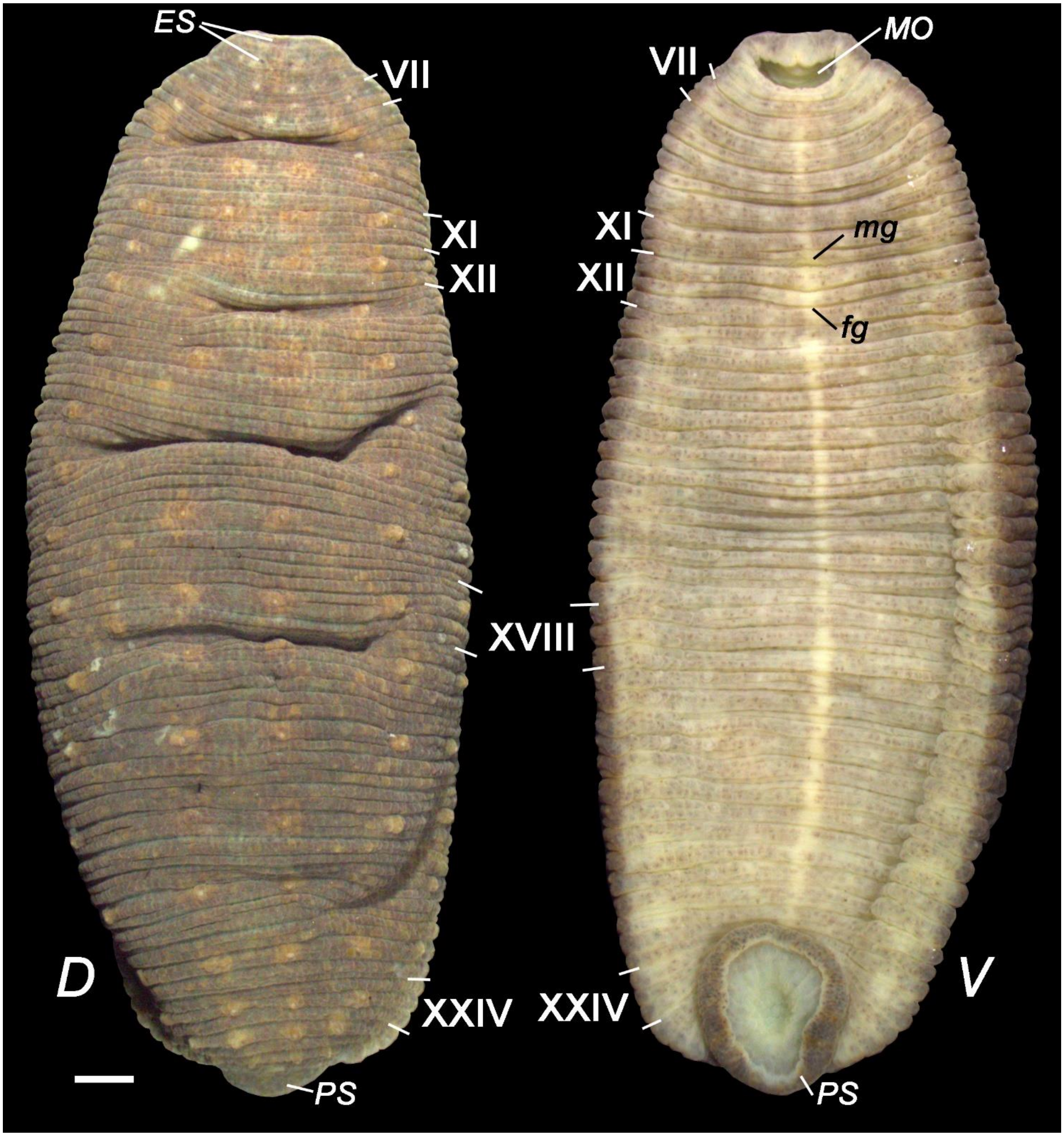

Figure S8. Holotype of *Hyperboreomyzon polaris* **gen. & sp. nov.** (RMBH Hir_0486-H, Putorana Plateau): external features and annulation in dorsal (*D*) and ventral (*V*) view. Abbreviations: *ES*, eyespots; *MO*, mouth; *mg*, male gonopore; *fg*, female gonopore; *PS*, posterior sucker. Body somites are indicated by roman numerals. Scale bar = 1.0 mm. Photos: Tatyana A. Eliseeva; graphics: Ivan N. Bolotov.

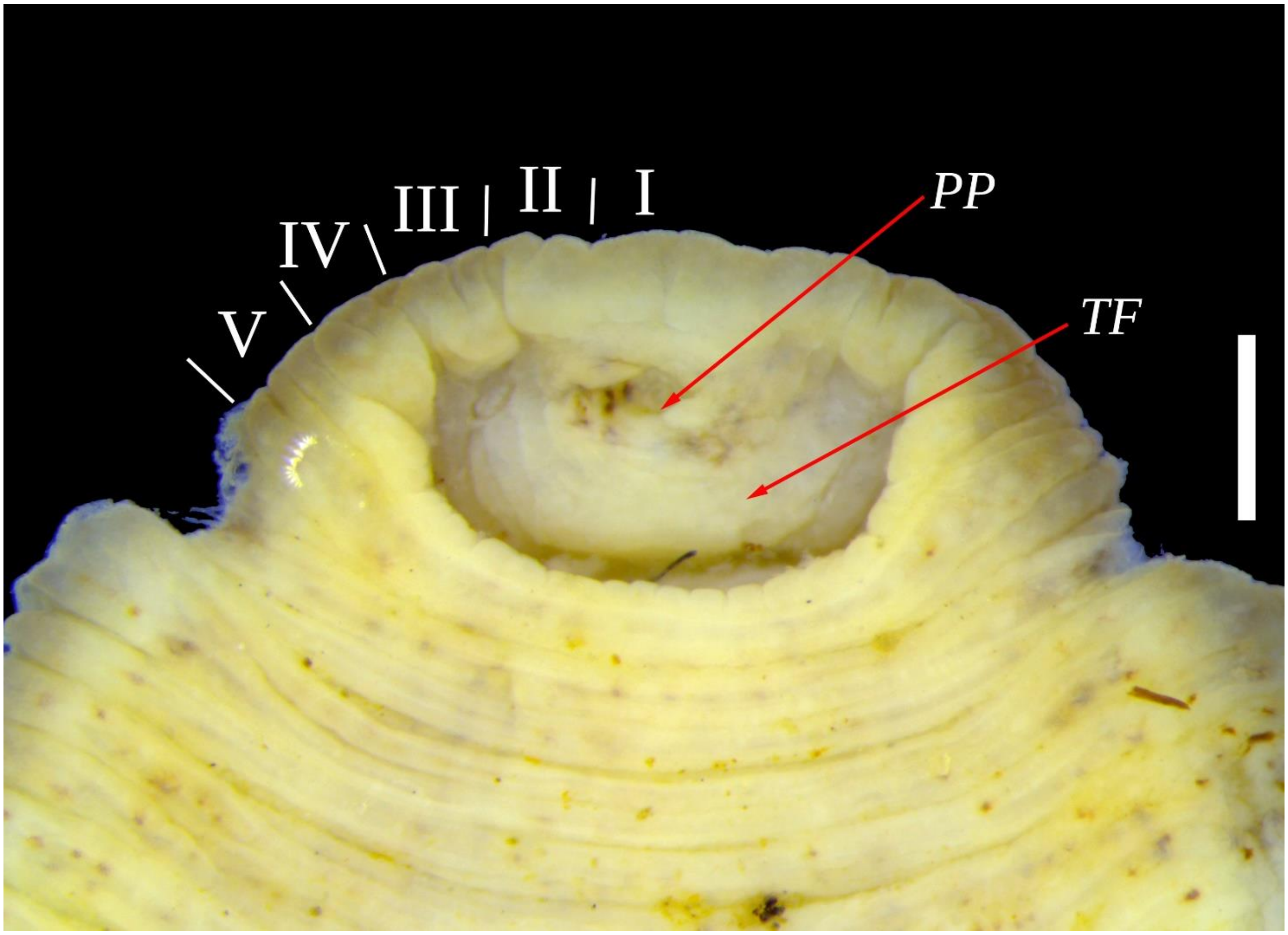

Figure S9. Anterior sucker of *Hyperboreomyzon polaris* **gen.** & **sp. nov.** (paratype RMBH Hir_0216, Kolguev Island) (ventral view). Abbreviations: *PP*, proboscis pore, *TF*, thickened fold inside anterior sucker. Body somites are indicated by roman numerals. Scale bar = 0.5 mm. Photo: Tatyana A. Eliseeva; graphics: Ivan N. Bolotov.

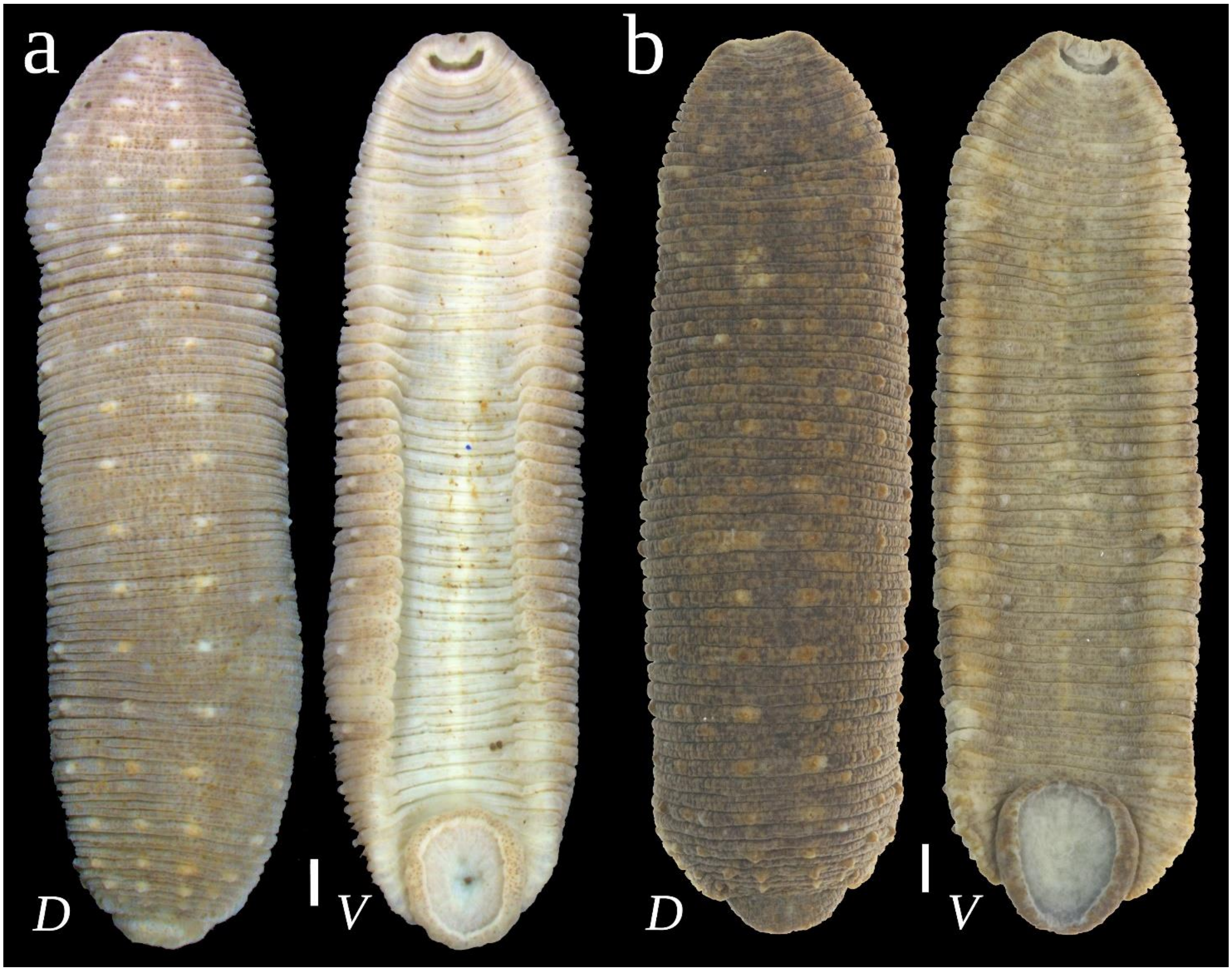

Figure S10. Dorsal (D) and ventral (V) views of two paratypes of *Hyperboreomyzon polaris* **gen.** & **sp. nov.** (**a**) paratype RMBH Hir_0216, Kolguev Island; and (**b**) paratype RMBH Hir_0689, Putorana Plateau. Scale bar = 1.0 mm. Photos: Anna L. Klass (a) and Tatyana A. Eliseeva (b).

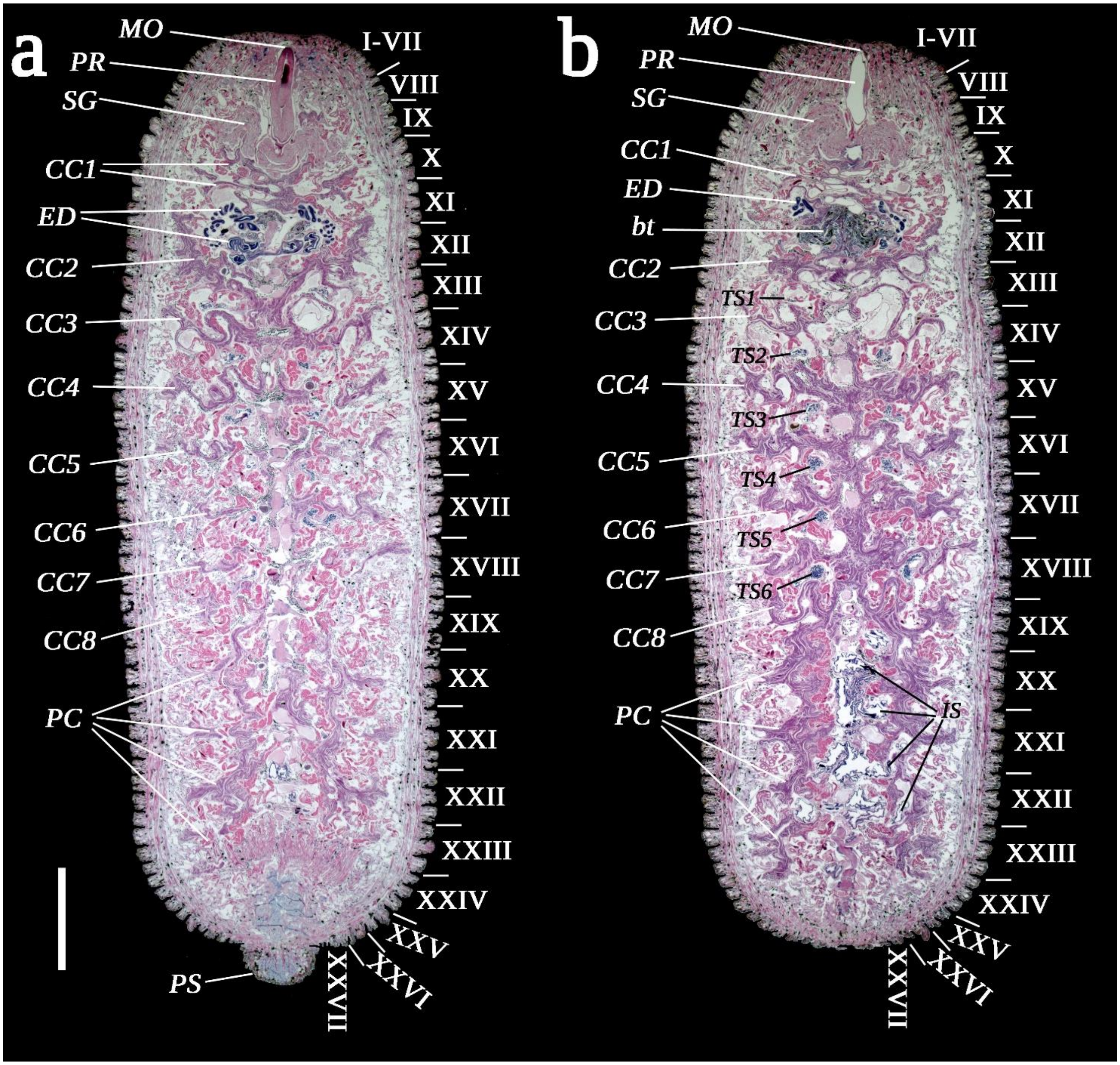

Figure S11. H&E stained longitudinal slices showing the digestive and generative systems of *Hyperboreomyzon polaris* **gen. & sp. nov.** (paratype RMBH Hir_0689, Putorana Plateau): (**a**) closer to the ventral side (slide 24-Hir_0689); and (**b**) closer to the dorsal side (slide 30-Hir_0689). Abbreviations: *MO*, mouth; *PR*, proboscis sheath; *SG*, salivary glands; *CC1-CC8*, 1st–8th pairs of crop caeca; *PC*, 9th pair of crop caeca (posterior caeca); *IS*, intestine; *bt*, black tissue covering atrium; *ED*, ejaculatory ducts; *TS1-TS6*, 1st–6th pairs of testisacs; and *PS*, posterior sucker. Body somites are indicated by roman numerals. Scale bar = 2.0 mm. Photo: Alexander V. Kropotin; graphics: Ivan N. Bolotov.

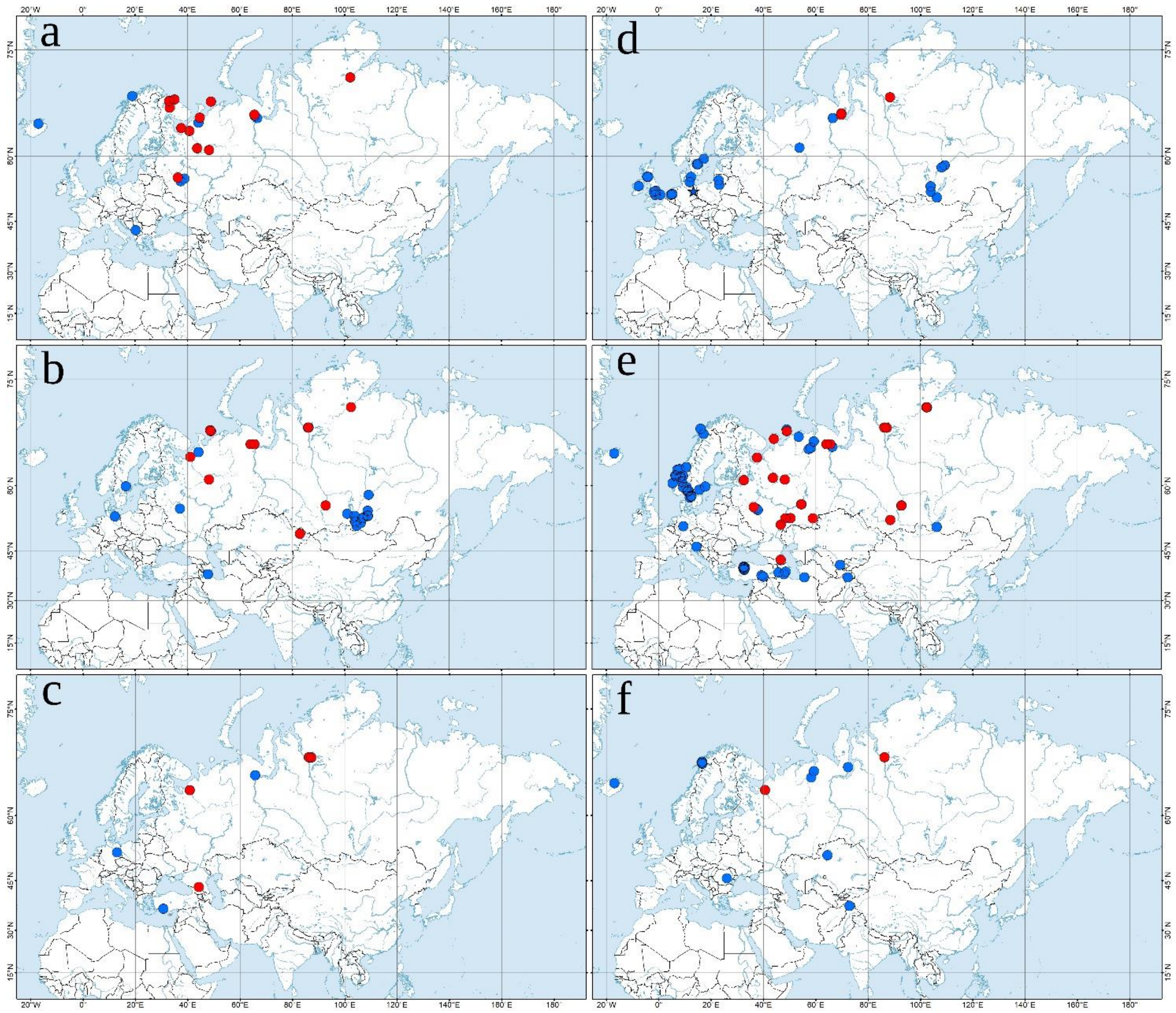

Figure S12. Maps of occurrences of the Glossiphoniidae species recorded from the Arctic (species with Palearctic ranges). (**a**) *Glossiphonia balcanica*; (**b**) *G. concolor*; (**c**) *G. nebulosa*; (**d**) *G. verrucata*; (**e**) *Helobdella stagnalis*; and (**f**) *Theromyzon maculosum*. The red circles indicate original records; the blue circles indicate published records; the blue star indicate the type locality of *G. verrucata* (see Dataset 2 for raw occurrence data and literature sources). The map was created using ESRI ArcGIS 10 software (www.esri.com/arcgis).

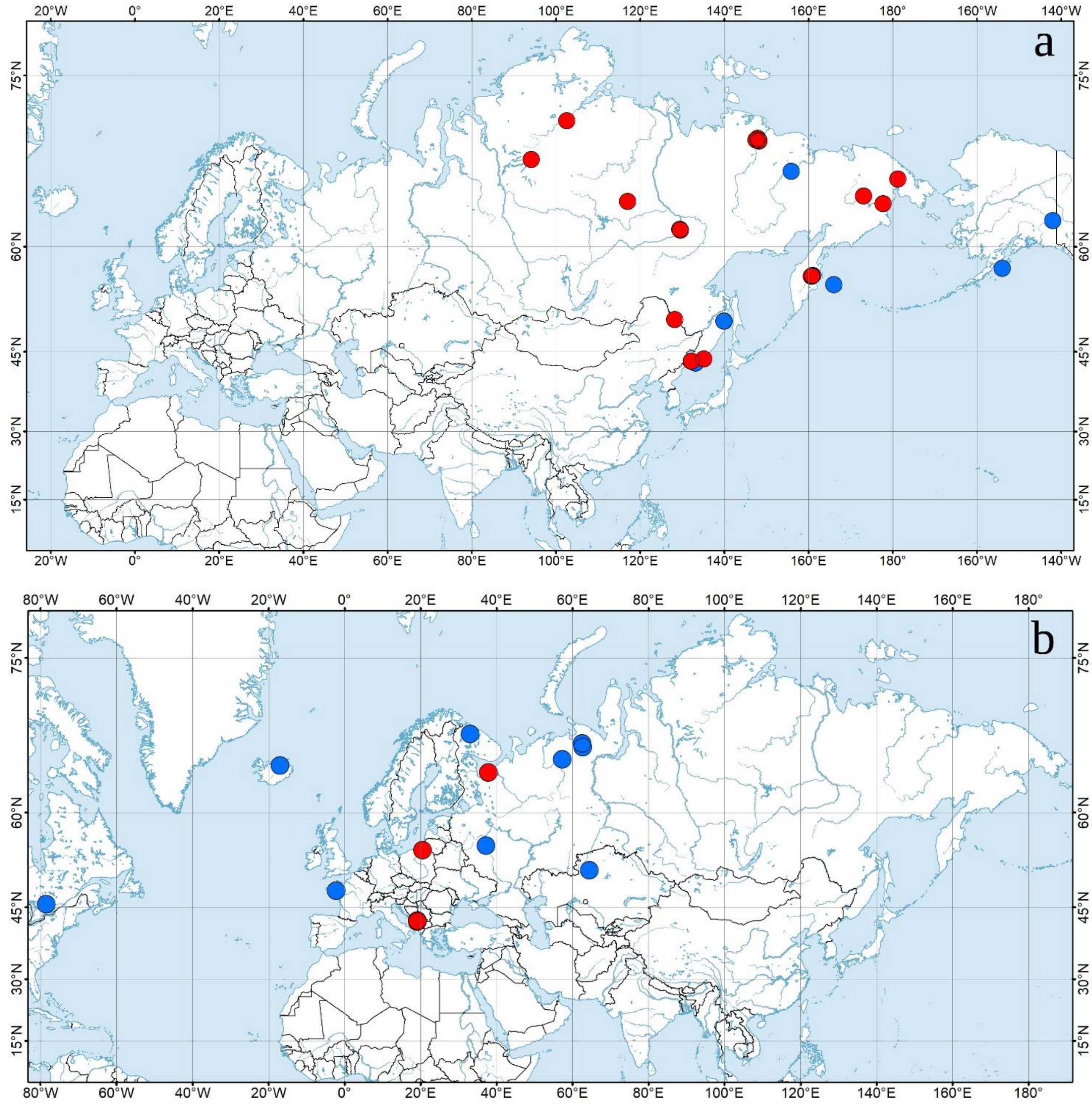

Figure S13. Maps of occurrences of the Glossiphoniidae species recorded from the Arctic (species with Holarctic ranges). (**a**) *Glossiphonia mollissima*; and (**b**) *Theromyzon tessulatum*. The red circles indicate original records; the blue circles indicate published records (see Dataset 2 for raw occurrence data and literature sources). The map was created using ESRI ArcGIS 10 software (www.esri.com/arcgis).

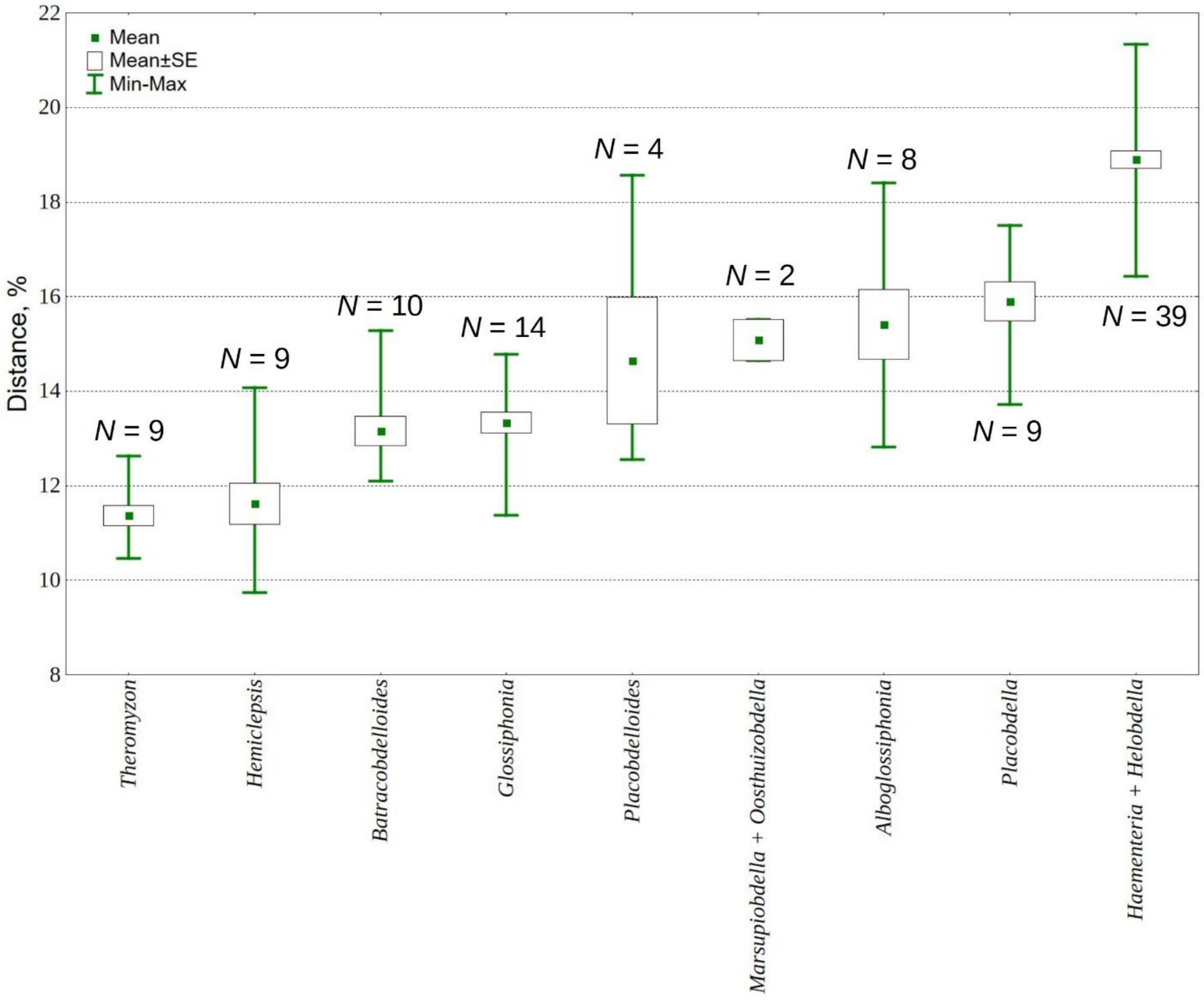

Figure S14. Box plot of uncorrected *COI* p-distances (%) between *Hyperboreomyzon polaris* **gen. & sp. nov.** and other genera of the Glossiphoniidae. The distances were calculated between a haplotype of *H. polaris* and selected species-level haplotypes in each genus (Table S1).

Table S1. Information on the *COI* and *18S rRNA* gene sequences used in a two-locus phylogenetic reconstruction of the Hirudinea (accession numbers of the new sequences generated in this study are bold; other sequences were obtained from GenBank)

Taxa	Haplotype Code	Region	*COI*	*18S rRNA*
**In-group taxa (Hirudinida)**				
**Glossiphoniidae**				
**Genus *Batracobdelloides* Oosthuizen, 1986**				
B. amnicolus (Moore, 1958**)**	BatAmn	South Africa	AY962457	AY962430
B. bangkhenensis Chiangkul, Trivalairat & Purivirojkul, 2021	BatBan	Thailand	MT292810	n/a
B. conchophylus Bolotov et al., 2019	BatCon	Myanmar	MN295408	MN312185
B. hlaingbweensis Bolotov et al., 2019	BatHla	Myanmar	MN295453	MN595225
B. indochinensis Bolotov et al., 2019	BatInd	Myanmar	MN295409	MN312186
B. koreanus Bolotov et al., 2019	BatKor	South Korea	MN295424	MN312194
B. tricarinatus (Blanchard, 1897)	BatTri	Uganda	MN295446	MN312199
B. yaukthwa Bolotov et al., 2019	BatYau	Myanmar	MN295406	MN312184
B. sp.2 'Myanmar'	BatSp2	Myanmar	**ON841607**	**ON854149**
B. sp.3 'Myanmar'	BatSp3	Myanmar	**ON841619**	n/a
**Genus *Hyperboreomyzon*** gen. nov.				
H. polaris **gen. & sp. nov.**	HypPo1	Northern European Russia: Kolguev Island	**ON810677**	**ON819005**
H. polaris **gen. & sp. nov.**	HypPo2	Russia: Putorana Plateau, Eastern Siberia	**ON810753**	**ON819030**
**Genus *Torix* Blanchard, 1893**				
T. tukubana (Oka, 1935)	TorTuk	Japan	LC538263	LC538283
**Genus *Hemiclepsis* Vejdovsky, 1884**				
H. kasmiana Oka, 1910 [Russian lineage]	HemKas	Russian Far East	MN295425	MN312195
H. khankiana Bolotov et al., 2019	HemKha	Russian Far East	MN295420	MN312192
H. marginata (O. F. Müller, 1773)	HemMar	Russia: Moscow Region	MN295388	MN312178
H. myanmariana Bolotov et al., 2019	HemMya	Myanmar	MN295398	MN312182
H. schrencki Bolotov et al., 2019	HemSch	Russian Far East	MN295415	MN312189
H. tumniniana Bolotov et al., 2019	HemTum	Russian Far East	MN295417	MN312191
H. yangtzenensis Yang & Bolotov, 2021	HemYan	China	MW699107	MW699854
H. sp. 'Khabarovsk'	HemKhb	Russian Far East	**ON841627**	**ON854150**
H. sp. 'Korea'	HemSp1	South Korea	KF966547	n/a
**Genus *Alboglossiphonia* Lukin, 1976**				
A. heteroclita (Linnaeus, 1761)	AlbHet	Russia: Western Siberia	**ON631769**	**ON704956**
A. lata (Oka, 1910)	AlbLat	Russian Far East	MN295414	MN312188
A. pahariensis Nesemann & Sharma, 2007	AlbPah	India	**ON548504**	**ON532907**
A. pallida (Verrill, 1872)	AlbPal	USA: Michigan	AF116016	AF115983
A. quadrata (Moore, 1924)	AlbQua	Namibia	AY962455	AY962412
A. sibirica **sp. nov.**	AlbPap	Russia: Eastern Siberia	MH286267	MH286273

Taxa	Haplotype Code	Region	*COI*	*18S rRNA*
A. sp.1 'Australia'	AlbMas	Australia	MG976199	n/a
A. sp.2 'Korea'	AlbKor	South Korea	KF966548	n/a
A. sp.3 'Myanmar'	AlbMya	Myanmar	**ON548507**	**ON704955**
**Genus *Glossiphonia* Johnson, 1816**				
G. arctica **sp. nov.**	GloArc	Russia: Polar Urals	**ON810735**	**ON819028**
G. baicalensis (Stschegolew, 1922)	GloBai	Russia: Lake Baikal, Eastern Siberia	AY047329	AY962425
G. balcanica Grosser & Pešić, 2016	GloBal	Russia: Moscow Region	MN295386	MN312176
G. complanata (Linnaeus, 1758)	GloCom	Montenegro	**ON841652**	n/a
G. concolor (Apathy, 1888)	GloRos	Russia: Taymyr Peninsula	**ON810692**	**ON819012**
G. cf. *concolor* (Apathy, 1888)	GloCon	Ukraine	KM095097	n/a
G. elegans (Verrill, 1872)	GloEle	Canada	**ON841597**	n/a
G. mollissima Moore, 1951	GloChe	Russia: Khabarovsk Region	MN295375	MN312170
G. nebulosa Kalbe, 1964	GloNeb	Russia: Taymyr Peninsula	**ON810698**	**ON819015**
G. taymyrensis **sp. nov.**	GloTay	Russia: Taymyr Peninsula	**ON810706**	**ON819021**
G. verrucata (F. Müller, 1844)	GloNor	Russia: Yamal Peninsula	MN295411	**ON819003**
G. sp.1 'Montenegro'	GloMon	Montenegro	**ON841644**	n/a
G. sp.2 'Korea'	GloSp2	South Korea	MN295429	n/a
G. sp.3 'Balkans'	GloVer	Italy	AY962459	AY962432
**Genus *Theromyzon* Philippi, 1867**				
T. maculosum (Rathke, 1862)	TheMac	Northern European Russia: Arkhangelsk Region	**ON810732**	**ON819026**
T. mollissimum (Grube, 1871)	TheMol	Northern European Russia: Arkhangelsk Region	**ON810733**	**ON819027**
T. tessulatum (O. F. Müller, 1773)	TheTes	Russia: Moscow Region	MN393259	**ON819002**
T. sp. 'North Caucasus'	TheCau	Russia: North Ossetia–Alania	**ON841631**	**ON854151**
T. sp.1 'Ontario'	TheOnt	Canada	**ON841595**	**ON854146**
T. sp.2 'Canada'	TheCa2	Canada	KM612217	n/a
T. sp.3 'Canada'	TheCa3	Canada	HQ961484	n/a
**Genus *Haementeria* De Filippi, 1849**				
H. depressa (Blanchard, 1849)	HaeGra	Uruguay	AF329034	AF115984
H. ghilianii De Filippi, 1849	HaeGhi	Brazil	AF116017	AF115985
H. lutzi Pinto, 1920	HaeLut	Ecuador	AF329033	AY962433
**Genus *Helobdella* Blanchard 1896**				
H. atli Oceguera-Figueroa & León-Régagnon, 2005	HelAtl	Mexico	HQ179850	n/a
H. austinensis Kutschera et al., 2013	HelAus	USA	DQ995306	n/a

Taxa	Haplotype Code	Region	*COI*	*18S rRNA*
H. bolivianita Siddall, 2001	HelBol	Bolivia	AF329053	n/a
H. bowermani Moser et al., 2013	HelBow	USA	KF683192	n/a
H. californica Kutschera, 1988	HelCal	USA	HQ686307	n/a
H. echoensis Saglam et al., 2018	HelEch	USA	MN071288	n/a
H. elongata Castle, 1900	HelElo	USA: Michigan	AF329045	AY962419
H. eriensis Saglam et al., 2018	HelEri	USA	MN071312	n/a
H. europaea Kutschera, 1987	HelEur	Taiwan [non-native]	FJ000350	n/a
H. fusca (Castle, 1900)	HelFus	USA: Michigan	AF329038	AY962414
H. lineata (Verrill, 1874)	HelLin	USA: NY	MN071348	n/a
H. melananus Lai & Chang, 2009	HelMel	Taiwan [non-native?]	FJ000353	n/a
H. modesta Verrill, 1872	HelMod	USA: Beaver Lake, NY	MN071318	n/a
H. sp. 'Korea'	HelSp5	South Korea	MN295428	n/a
H. nunununojensis Siddall, 2001	HelNun	Bolivia	AF329048	AY962426
H. octatestisaca Lai & Chang, 2009	HelOct	Panama	MN071319	n/a
H. okhotica **sp. nov.**	HelSp6	Russia: Khabarovsk Region	MN295372	MN312167
H. papillata (Moore, 1952)	HelPap	USA: Michigan	AF329043	AY962417
H. paranensis (Oka, 1930)	HelPar	Uruguay	AF116019	AF115987
H. pichipanan Siddall & Borda, 2004	HelPic	Chile	AY962456	AY962429
H. robusta Shankland, Bissen & Weisblat, 1992	HelRob	USA: California	DQ995300	n/a
H. serendipitous Saglam et al., 2018	HelSer	USA: NJ	JN692265	n/a
H. socimulcensis (Caballero, 1931)	HelSoc	Mexico	MG821616	n/a
H. sorojchi Siddall, 2001	HelSor	Bolivia	AF329050	AY962427
H. stagnalis (Linnaeus, 1758)	HelSp3	Russia: Moscow Region	MN295387	MN312177
H. transversa Sawyer, 1972	HelTra	USA: Michigan	AF329044	AY962418
H. triserialis (E. Blanchard, 1849)	HelTri	Bolivia	AF329054	AY962435
H. sp. 'Alaska'	HelAla	USA: Alaska	**ON841604**	**ON854147**
H. sp. 'British Columbia'	HelBri	Canada	**ON841605**	**ON854148**
H. sp. 'Canada'	HelCan	Canada	MN071351	n/a
H. sp.1 'Montana'	HelMon	USA: Montana	JN692264	n/a
H. sp.2 sensu Iwama et al., 2020	HelS02	USA: Nebraska	MN071321	n/a
H. sp.3 sensu Iwama et al., 2020	HelS03	Canada	MN071322	n/a
H. sp.5 sensu Iwama et al., 2020	HelS05	Canada	MN071325	n/a
H. sp.6 sensu Iwama et al., 2020	HelS06	Canada	MN071338	n/a
H. sp.7 'Washington'	HelS07	Canada	MN071323	n/a
**Genus *Placobdella* Blanchard, 1893**				
P. biannulata (Moore, 1900)	PlaBia	USA: North Carolina	AF116021	AF115989
P. costata (F. Müller, 1846)	PlaCos	Italy	AY962461	AY962436
P. montifera (Moore, 1906)	PlaMon	USA: Michigan	AY047323	AY962420
P. multilineata Moore, 1953	PlaMul	USA: Louisiana	AY962464	AY962439
P. papillifera (Verrill, 1872)	PlaPap	USA: Tennessee	AY047324	AY962421

Taxa	Haplotype Code	Region	*COI*	*18S rRNA*
P. parasitica (Say, 1824)	PlaPar	Canada: Ontario	AF003261	AF115990
P. pediculata Hemingway, 1908	PlaPed	USA: Michigan	AY047327	AY962423
P. picta (Verrill, 1872)	PlaPic	Canada: Ontario	AF116020	AF115988
P. transluscens (Sawyer & Shelley, 1976)	PlaTra	USA: Michigan	AY047328	AY962424
**Genus *Placobdelloides* Sawyer, 1986**				
P. siamensis (Oka, 1917)	PlaSia	Thailand	AY962462	AY962437
P. sirikanchanae Trivalairat, Chiangkul & Purivirojkul, 2019	PlaSir	Thailand	MK282432	n/a
**Piscicolidae**				
**Genus *Alexandrobdella* Bolotov et al., 2020**				
A. makhrovi Bolotov et al., 2020	AleMak	Russian Far East	MN295413	MN312187
**Genus *Piscicola* Blainville, 1818**				
P. geometra (Linnaeus, 1761)	PisGeo	Germany	AY336014	AF099946
**Genus *Branchellion* Savigny, 1822**				
B. torpedinis Savigny, 1822	BraTor	USA: South Carolina	AF003265	AF115993
**Genus *Calliobdella* van Beneden & Hesse, 1863**				
C. vivida (Verrill, 1872)	CalViv	USA: Virginia	AF003260	AF115992
**Genus *Myzobdella* Leidy, 1851**				
M. lugubris Leidy, 1851	MyzLug	USA: Virginia	AF003269	AF115994
**Genus *Stibarobdella* Leigh-Sharpe, 1925**				
S. macrothela (Schmarda, 1861)	StiMac	USA: Virginia	AF116022	AF115996
**Erpobdellidae**				
**Genus *Erpobdella* Lamarck, 1818**				
E. japonica (Pawlowski, 1952)	ErpJap	Korea	AF116026	AF116000
E. monostriata (Lindenfeld & Pietruszynski, 1890)	ErpMon	Northern European Russia: Arkhangelsk Region	MN295419	n/a
E. punctata (Leidy, 1870)	ErpPun	Canada: Ontario	AF003275	AF116002
E. testacea (Savigny, 1822)	ErpTes	France	AF116027	AF116003
**Genus *Dina* Blanchard, 1892**				
D. dubia Moore & Meyer, 1951	DinDub	USA: Michigan	AF116023	AF115997
**Genus *Mooreobdella* Pawlowski, 1955**				
M. melanostoma Sawyer & Shelley, 1976	MooMel	USA: Michigan	AF116025	AF115999
M. bucera Moore, 1949	MooBuc	USA: Michigan	AF116024	AF115998
**Genus *Nephelopsis* Verrill, 1872**				
N. obscura Verrill, 1872	NepObs	Canada: Ontario	AF003273	AF116004
**Gastrostomobdellidae**				
**Genus *Gastrostomobdella* Moore, 1929**				
G. extenta Nakano & Jeratthitikul, 2018	GasExt	Thailand	LC274553	LC274519
G. ampunganensis Nakano, 2018	GasAmp	Malaysia	LC274551	LC274517
**Salifidae**				

**Taxa**	**Haplotype Code**	**Region**	***COI***	***18S rRNA***
**Genus *Barbronia* Johansson, 1918**				
Barbronia gwalagwalensis Westergren & Siddall, 2004	BarGwa	Myanmar	MN295405	**ON854145**
B. weberi (Blanchard, 1897)	BarWeb	USA [non-native]	AY786456	AY786461
**Genus *Salifa* Blanchard, 1897**				
S. motokawai Nakano & Nguyen, 2015	SalMot	Vietnam	LC029431	LC029434
**Genus indet.**				
Gen. & sp. indet.1	SalGe1	Myanmar	MN295395	n/a
Gen. & sp. indet.2	SalGe2	Myanmar	MN295396	n/a
**Orobdellidae**				
**Genus *Orobdella* Oka, 1895**				
O. koikei Nakano, 2012	OroKoi	Japan: Hokkaido	AB679688	AB698883
O. esulcata Nakano, 2010	OroEsu	Japan: Kumamoto	AB675020	AB663655
**Ozobranchidae**				
**Genus *Ozobranchus* Quatrefages, 1852**				
O. branchiatus (Menzies, 1791)	OzoBra	North America	KF728213	KF728214
O. margoi (Apáthy, 1890)	OzoMar	Taiwan	KJ451407	KF728217
**Hirudinidae**				
**Genus *Hirudo* Linnaeus, 1758**				
H. orientalis Utevsky & Trontelj, 2005	HirOri	Azerbaijan	GQ368750	GQ368792
H. troctina Johnson, 1816	HirTro	Morocco	GQ368751	GQ368793
**Genus *Aliolimnatis* Richardson, 1972**				
A. africana (Blanchard, 1897)	AliAfr	Central African Republic	AY425451	AY425469
A. michaelseni (Augener, 1936)	AliMic	Congo	AF116029	AF116010
**Genus *Macrobdella* Verrill, 1872**				
M. decora (Say, 1824)	MacDec	USA: Michigan	AF003271	AF116007
**Genus *Poecilobdella* Blanchard, 1893**				
P. nanjingensis Yang, 1996	PoeNan	Taiwan	LC145739	LC145737
**Genus *Hirudinaria* Whitman, 1886**				
H. javanica (Wahlberg, 1856)	HirJav	Vietnam	GQ368745	GQ368787
**Genus *Goddardobdella* Richardson, 1969**				
G. elegans (Grube, 1867)	GodEle	Australia	GQ368743	GQ368785
**Haemadipsidae**				
**Genus *Chtonobdella* Grube, 1866**				
C. bilineata (Richardson, 1975)	ChtBil	Australia	AF003267	AF116006
**Genus *Haemadipsa* Tennent, 1859**				
H. sylvestris Blanchard, 1894	HaeSyl	Vietnam	AF003266	AF116005
**Haemopidae**				
**Genus *Haemopis* Savigny, 1822**				
H. lateromaculata Mathers 1963	HaeLat	USA: Michigan	AF116028	AF116009
H. marmorata (Say 1824)	HaeMar	USA: Michigan	AF003270	AF116008

Taxa	Haplotype Code	Region	*COI*	*18S rRNA*
**Outgroup taxon (Acanthobdellida)**				
**Acanthobdellidae**				
**Genus *Acanthobdella* Grube, 1851**				
A. peledina Grube, 1851	AcaPel	Sweden	MH351652	MH351629

n/a – not available.

Table S2. Voucher numbers, accession numbers of reference DNA sequences (the numbers of newly generated sequences are bold), and measurements for the type series of the new taxa. The type series are deposited in the RMBH – Russian Museum of Biodiversity Hotspots, N. Laverov Federal Center for Integrated Arctic Research of the Ural Branch of the Russian Academy of Sciences (Arkhangelsk, Russia)

Species and status of specimen	Voucher no.	Type of voucher	Region of Northern Eurasia	Reference *COI* sequence acc. no.	Measurements (mm)			
					Body length (BL)	Body width (BW)	Width of anterior sucker (AW)	Width of posterior sucker (PW)
Alboglossiphonia sibirica **sp. nov.**								
Holotype	RMBH Hir_0542_2-H	Ethanol-preserved specimen	Primorye	N/A	4.03	2.42	0.33	1.16
Paratype	RMBH Hir_0542_2	DNA	Primorye	**ON873332**	3.96	3.17	0.52	1.07
Paratype	RMBH Hir_0409_1	Ethanol-preserved specimen	Primorye	**ON548509**	5.50	2.50	0.45	1.00
Paratype	RMBH Hir_0413	Ethanol-preserved specimen	Primorye	**ON548510**	3.24	1.94	0.28	0.80
Paratype	RMBH Hir_0509_1	Ethanol-preserved specimen	Amur Region	**ON548516**	4.07	2.57	0.46	0.97
Paratype	RMBH Hir_0396	Ethanol-preserved specimen	Yamal-Nenets Region	N/A	1.69	2.34	0.39	0.58
Paratype	RMBH Hir_0013	Series of permanent slides	Yakutia Republic	N/A	4.34	2.36	0.27	0.46
Paratype	RMBH Hir_0013_1	DNA	Yakutia Republic	MH286267	7.50	5.30	1.00	2.25
Paratype	RMBH Hir_0013_2	DNA	Yakutia Republic	MH286268	7.90	3.10	0.67	1.55
Paratype	RMBH Hir_0013_3	DNA	Yakutia Republic	MH286269	8.50	5.21	1.00	2.05
Paratype	RMBH Hir_0003_3	DNA	Khabarovsk Region	MN393256	4.50	3.70	0.43	1.20
Paratype	RMBH Hir_0394	DNA	Irkutsk Region	**ON548508**	11.87	5.62	0.79	1.67
Paratype	RMBH Hir_0417_2	DNA	Irkutsk Region	**ON548511**	2.67	1.37	0.29	0.50
Paratype	RMBH Hir_0510_1	DNA	Amur Region	**ON548517**	3.46	2.53	0.44	1.04
				Mean	5.23	3.15	0.52	1.16
				s.e.m.	0.77	0.37	0.07	0.15
				min	1.69	1.37	0.27	0.46
				max	11.87	5.62	1.00	2.25
Glossiphonia arctica **sp. nov.**								

Species and status of specimen	Voucher no.	Type of voucher	Region of Northern Eurasia	Reference *COI* sequence acc. no.	**Measurements (mm)**			
					**Body length (BL)**	**Body width (BW)**	**Width of anterior sucker (AW)**	**Width of posterior sucker (PW)**
Holotype	RMBH Hir_0457_2_1-H	Ethanol-preserved specimen	Polar Urals	**ON810735**	8.11	5.65	0.62	1.66
Paratype	RMBH Hir_0457_2_2	Ethanol-preserved specimen	Polar Urals	**ON810736**	9.40	6.29	0.52	1.90
Paratype	RMBH Hir_0457_2_3	Ethanol-preserved specimen	Polar Urals	**ON810737**	10.26	5.86	0.61	1.76
Paratype	RMBH Hir_0457	Ethanol-preserved specimen	Polar Urals	N/A	8.29	4.36	0.49	1.48
Paratype	RMBH Hir_0457	Ethanol-preserved specimen	Polar Urals	N/A	9.93	5.92	0.57	1.88
Paratype	RMBH Hir_0457	Ethanol-preserved specimen	Polar Urals	N/A	9.74	6.05	0.58	1.81
Paratype	RMBH Hir_0457	Ethanol-preserved specimen	Polar Urals	N/A	10.35	6.55	0.58	1.38
Paratype	RMBH Hir_0457	Ethanol-preserved specimen	Polar Urals	N/A	12.20	6.93	0.61	1.64
Paratype	RMBH Hir_0457	Ethanol-preserved specimen	Polar Urals	N/A	12.30	6.73	0.64	1.68
Paratype	RMBH Hir_0457	Ethanol-preserved specimen	Polar Urals	N/A	11.33	6.32	0.67	1.69
Paratype	RMBH Hir_0457	Ethanol-preserved specimen	Polar Urals	N/A	8.64	5.10	0.50	1.34
Paratype	RMBH Hir_0457	Ethanol-preserved specimen	Polar Urals	N/A	7.49	4.52	0.43	1.26
Paratype	RMBH Hir_0457	Ethanol-preserved specimen	Polar Urals	N/A	13.31	6.48	0.64	1.61
Paratype	RMBH Hir_0457	Ethanol-preserved specimen	Polar Urals	N/A	9.81	6.43	0.68	1.51
Paratype	RMBH Hir_0457	Ethanol-preserved specimen	Polar Urals	N/A	9.01	4.87	0.58	1.34
Paratype	RMBH Hir_0457	Ethanol-preserved specimen	Polar Urals	N/A	9.23	6.13	0.60	1.42

Species and status of specimen	Voucher no.	Type of voucher	Region of Northern Eurasia	Reference *COI* sequence acc. no.	Measurements (mm)			
					Body length (BL)	Body width (BW)	Width of anterior sucker (AW)	Width of posterior sucker (PW)
Paratype	RMBH Hir_0457	Ethanol-preserved specimen	Polar Urals	N/A	8.13	5.20	0.53	1.11
Paratype	RMBH Hir_0457	Ethanol-preserved specimen	Polar Urals	N/A	6.33	4.03	0.60	1.04
Paratype	RMBH Hir_0457	Series of permanent slides	Polar Urals	N/A	8.91	5.13	0.59	0.94
				Mean	9.62	5.71	0.58	1.50
				s.e.m.	0.40	0.20	0.01	0.06
				min	6.33	4.03	0.43	0.94
				max	13.31	6.93	0.68	1.90
Glossiphonia taymyrensis **sp. nov.**								
Holotype	RMBH Hir_0258_1-H	Ethanol-preserved specimen	Taymyr Peninsula	**ON810695**	8.19	6.01	0.85	1.66
Paratype	RMBH Hir_0256_1	Ethanol-preserved specimen	Taymyr Peninsula	**ON810693**	10.80	5.44	0.69	1.65
Paratype	RMBH Hir_0256_1	Series of permanent slides	Taymyr Peninsula	N/A	5.59	4.50	0.73	1.53
Paratype	RMBH Hir_0261_2	Ethanol-preserved specimen	Taymyr Peninsula	**ON810699**	4.99	4.74	0.83	1.32
Paratype	RMBH Hir_0263_1	Ethanol-preserved specimen	Taymyr Peninsula	**ON810701**	11.30	6.19	0.95	2.08
Paratype	RMBH Hir_0264_3	Ethanol-preserved specimen	Taymyr Peninsula	**ON810705**	6.28	4.99	0.66	1.45
Paratype	RMBH Hir_0265_2	Ethanol-preserved specimen	Taymyr Peninsula	**ON810706**	5.76	4.12	0,55	1.27
Paratype	RMBH Hir_0488	Ethanol-preserved specimen	Putorana Plateau	**ON810755**	2.42	8.38	0.97	3.02
Paratype	RMBH Hir_0449	Ethanol-preserved specimen	Kemerovo Region	**ON810731**	8.33	5.60	0.51	1.92
				Mean	7.07	5.55	0.77	1.77
				s.e.m.	0.95	0.42	0.05	0.18
				min	2.42	4.12	0.51	1.27
				max	11.30	8.38	0.97	3.02

Species and status of specimen	Voucher no.	Type of voucher	Region of Northern Eurasia	Reference *COI* sequence acc. no.	Measurements (mm)			
					Body length (BL)	Body width (BW)	Width of anterior sucker (AW)	Width of posterior sucker (PW)
Hyperboreomyzon polaris **gen. & sp. nov.**								
Holotype	RMBH Hir_0486-H	Ethanol-preserved specimen	Putorana Plateau	**ON810753**	18.66	8.74	1.49	3.36
Paratype	RMBH Hir_0216	Ethanol-preserved specimen (dissected)	Kolguev Island	**ON810677**	20.58	8.10	1.72	3.20
Paratype	RMBH Hir_0689	Series of permanent slides	Putorana Plateau	N/A	20.51	6.55	1.47	3.40
				Mean	19.92	7.80	1.56	3.32
				s.e.m.	0.77	0.80	0.10	0.07
				min	18.66	6.55	1.47	3.20
				max	20.58	8.74	1.72	3.40
Helobdella okhotica **sp. nov.**								
Holotype	RMBH Hir_0251_1-H	Ethanol-preserved specimen	Chukotka Peninsula	N/A	6.87	3.49	0.51	1.49
Paratype	RMBH Hir_0251_1	Ethanol-preserved specimen	Chukotka Peninsula	**ON810688**	7.73	4.39	0.53	1.17
Paratype	RMBH Hir_0251_1	Ethanol-preserved specimen	Chukotka Peninsula	N/A	6.66	3.41	0.56	1.26
Paratype	RMBH Hir_0251_1	Ethanol-preserved specimen	Chukotka Peninsula	N/A	3.89	1.72	0.44	0.69
Paratype	RMBH Hir_0294	Ethanol-preserved specimen	Kamchatka Peninsula	**ON810719**	7.16	3.42	0.64	1.41
Paratype	RMBH Hir_0003_2	DNA	Khabarovsk Region	MN295372	7.51	4.98	0.68	1.57
Paratype	RMBH Hir_0003_2	DNA	Khabarovsk Region	MN393255	5.96	3.48	0.74	1.48
Paratype	RMBH Hir_0003_2	DNA	Khabarovsk Region	N/A	3.32	2.31	0.53	0.91
Paratype	RMBH Hir_0295	Ethanol-preserved specimen	Kamchatka Peninsula	**ON810720**	7.05	4.31	0.66	N/A
Paratype	RMBH Hir_0295	Ethanol-preserved specimen	Kamchatka Peninsula	N/A	5.75	2.24	0.44	0.95

Species and status of specimen	Voucher no.	Type of voucher	Region of Northern Eurasia	Reference *COI* sequence acc. no.	Measurements (mm)			
					Body length (BL)	Body width (BW)	Width of anterior sucker (AW)	Width of posterior sucker (PW)
Paratype	RMBH Hir_0295	Ethanol-preserved specimen	Kamchatka Peninsula	N/A	3.00	1.43	0.43	0.64
Paratype	RMBH Hir_0491_1	Series of permanent slides	Kamchatka Peninsula	N/A	5.1	2.6	0.34	0.58
				Mean	5.83	3.15	0.54	1.10
				s.e.m.	0.48	0.32	0.03	0.11
				min	3.00	1.43	0.34	0.58
				max	7.73	4.98	0.74	1.57

N/A – not available.

Table S3. Morphological and anatomical characters of Glossiphoniidae genera

Subfamily and genus	Pairs of eyespots	Number of annuli between the gonopores	Pairs of testisacs	Salivary cells	Position of proboscis pore	Dorsal annulation of mid-body somites	Reference
Hyperboreomyzon **gen. nov.**	2	2	6	Compact	Subapical (massive velar fold in oral sucker close to its anterior margin)	Secondarily sexannulate	This study
Alboglossiphonia Lukin, 1976	3	0-2	6	Diffuse	Central (center of oral sucker)	Triannulate	Light & Siddall [1]; Nesemann & Neubert [2]
Batracobdella Viguier, 1879	1	2	6	Diffuse	Central (center of oral sucker)	Triannulate	Nesemann & Neubert [2]
Batracobdelloides Oosthuizen, 1986	2	2	6	Diffuse	Central (center of oral sucker)	Triannulate	Bolotov et al. [3]
Glossiphonia Johnson, 1816	2-3	2	6	Diffuse	Central (center of oral sucker)	Triannulate (biannulate in one species)	Light & Siddall [1]
Hemiclepsis Vejdovsky, 1884	2-3	2	6	Diffuse	Subcentral (posterior part of oral sucker)	Triannulate	Bolotov et al. [3]
Theromyzon Philippi, 1867	4	2-5	6	Diffuse	Apical (anterior rim of oral sucker)	Triannulate (with shallow secondary grooves in a few species)	Livanow [4]; Oosthuizen & Davies [5]
Torix Blanchard, 1893	2	1	6	Compact	Apical (anterior rim of oral sucker)	Biannulate	Kambayashi et al. [6]
Haementeria De Filippi, 1849	1	1-3	5-6	Compact	Apical (anterior rim of oral sucker)	Triannulate (with shallow secondary grooves in a few species)	Sawyer [7]; Oceguera-Figueroa [8]
Helobdella Blanchard 1896	1	1	6	Diffuse (compact in a few species)	Central (center of oral sucker)	Triannulate	Sawyer [7]; Siddall et al. [9]
Placobdella Blanchard, 1893	1-2	1-3	6	Compact (diffuse in one species)	Apical (anterior rim of oral sucker)	Triannulate (biannulate in one species)	Nesemann & Neubert [2]; Siddall et al. [9]

Subfamily and genus	Pairs of eyespots	Number of annuli between the gonopores	Pairs of testisacs	Salivary cells	Position of proboscis pore	Dorsal annulation of mid-body somites	Reference
Actinobdella Moore, 1901	1	2	6	Diffuse	Apical (anterior rim of oral sucker)	Secondarily sexannulate but this pattern has intraspecific variability and is not expressed in some individuals	Sawyer [10]; Daniels & Freeman [11]
Adaetobdella Ringuelet, 1978	1	1	5-6	Compact	Subapical (base of anterior rim of oral sucker)	Triannulate	Ringuelet [12]
Baicaloclepsis Lukin & Epstein, 1960	0-2	2	N/A	N/A	Subapical	Triannulate	Lukin [13]
Gloiobdella Ringuelet, 1978	1	1	6-7	Diffuse	Central (center of oral sucker)	Triannulate	Sawyer [7]; Ringuelet [12]
Marsupiobdella Goddard & Malan, 1912	1	3	6	Diffuse	Central (center of oral sucker; surrounded by shallow velar folds)	Triannulate	Van Der Lande & Tinsley [14]; Oosthuizen & Siddall [15]
Marvinmeyeria Soos, 1969	1	0	5	?Diffuse	Apical (anterior rim of oral sucker)	Triannulate	Meyer & Moore [16]
Oosthuizobdella Sawyer, 1986	4	2	6	Compact	Apical (anterior rim of oral sucker)	Triannulate	Sawyer [7]; Oosthuizen [17]
Parabdella Autrum, 1936	2	2	6	Compact	Apical (anterior rim of oral sucker)	Triannulate	Sawyer [7]; Yang [18]
Paraclepsis Harding, 1924	3	2	5	Compact	Subapical	Triannulate	Harding [19]
Placobdelloides Sawyer, 1986	1	2	6	Compact (diffuse in a few cases)	Apical (anterior rim of oral sucker)	Usually triannulate	Trivalairat et al. [20]; Chiangkul et al. [21]
Tribothrynobdella Ringuelet, 1976	1	1	6	Diffuse	Central (center of oral sucker)	Triannulate	Ringuelet [12]

Supplementary References

1. Light, J. E. & Siddall, M. E. Phylogeny of the leech family Glossiphoniidae based on mitochondrial gene sequences and morphological data. *Journal of Parasitology* **85**, 815–823; https://doi.org/10.2307/3285816 (1999).

2. Nesemann, H. & Neubert, E. *Süßwasserfauna von Mitteleuropa, Bd. 6/2, Annelida: Clitellata: Branchiobdellida, Acanthobdellea, Hirudinea* (Spektrum Akademischer Verlag, 1999).

3. Bolotov, I. N. et al. Freshwater mussels house a diverse mussel-associated leech assemblage. *Scientific Reports* **9**, 16449; https://doi.org/10.1038/s41598-019-52688-3 (2019).

4. Livanow, L. Die Hirudineen-Gattung *Hemiclepsis* Vejd. *Zoologische Jahrbücher*. *Abtheilung für Systematik, Geographie und Biologie der Thiere* **3**, 339–362 (1902).

5. Oosthuizen, J. H. & Davies, R. W. A new species of *Theromyzon* (Rhynchobdellida: Glossiphoniidae), with a review of the genus in North America. *Canadian Journal of Zoology* **71**, 1311–1318; https://doi.org/10.1139/z93-18 (1993).

6. Kambayashi, C., Kurabayashi, A. & Nakano, T. Topotype-based redescription of the leech *Torix tukubana* (Hirudinida: Glossiphoniiformes: Glossiphoniidae). *Proceedings of the Biological Society of Washington* **133**, 59–71; https://doi.org/10.2988/20-00003 (2020).

7. Sawyer, R. T. *Leech Biology and Behaviour. Vol. 2. Feeding Biology, Ecology, and Systematics* (Clarendon Press, 1986).

8. Oceguera-Figueroa, A. A new glossiphoniid leech from Catemaco Lake, Veracruz, Mexico. *Journal of Parasitology* **94**, 375–380; https://doi.org/10.1645/GE-1240.1 (2008).

9. Siddall, M. E., Budinoff, R. B. & Borda, E. Phylogenetic evaluation of systematics and biogeography of the leech family Glossiphoniidae. *Invertebrate Systematics* **19**, 105–112; https://doi.org/10.1071/IS04034 (2005).

10. Sawyer, R. T. *North American freshwater leeches, exclusive of the Piscicolidae, with a key to all species* (University of Illinois Press, 1972).

11. Daniels, B. & Freeman, R. S. A review of the genus Actinobdella Moore, 1901 (Annelida, Hirudinea). *Canadian Journal of Zoology* **54**, 2112–2117; https://doi.org/10.1139/z76-244 (1976).

12. Ringuelet, R. Annulata Hirudinea. *Fauna de agua dulce de la Republica Argentina* **17**, 1–321 (1985).

13. Lukin, E. I. Leeches of fresh and brackish water bodies. *Fauna of the USSR* **109**, 1–484 (1976).

14. Van Der Lande, V. & Tinsley, R. C. Studies on the anatomy, life history and behaviour of *Marsupiobdella africana* (Hirudinea: Glossiphoniidae). *Journal of Zoology* **180**, 537–563; https://doi.org/10.1111/j.1469-7998.1976.tb04703.x (1976).

15. Oosthuizen, J. H. & Siddall, M. E. Chapter 14: Hirudinea. In *Guides to the freshwater Invertebrates of Southern Africa. Vol. 5: Non-Arthropods* (eds Day, J. A. & De Moor, I. J.) 237–263 (Water Research Commission, 2003).

16. Meyer, M. C. & Moore, J. P. Notes on Canadian leeches (Hirudinea), with the description of a new species. *Wasmann Journal of Biology* **12**, 63–96 (1954).

17. Oosthuizen, J. H. An annotated check list of the leeches (Annelida: Hirudinea) of the Kruger National Park with a key to the species. *Koedoe* **34**, 25–38 (1991).

18. Yang, T. *Annelida Hirudinea (Fauna Sinica)* (Beijing, Science Press, 1996).

19. Harding, W.A. XLIX – Descriptions of some new leeches from India, Burma, and Ceylon. *Annals and Magazine of Natural History* **14**, 489–499; https://doi.org/10.1080/00222932408633143 (1924).

20. Trivalairat, P., Chiangkul, K. & Purivirojkul, W. *Placobdelloides sirikanchanae* sp. nov., a new species of glossiphoniid leech and a parasite of turtles from lower southern Thailand (Hirudinea, Rhynchobdellida). *ZooKeys* **882**, 1–24; https://doi.org/10.3897/zookeys.882.35229 (2019).

21. Chiangkul, K., Trivalairat, P., Srakaew, N. & Purivirojkul, W. Fine morphological and histological characteristics of *Placobdelloides siamensis* (Annelida: Glossiphoniidae). *Zoologischer Anzeiger* **295**, 34–42; https://doi.org/10.1016/j.jcz.2021.09.003 (2021).
