# Supplementary material for: Cryptic taxonomic diversity and high-latitude melanism in the glossiphoniid leech assemblage from the Eurasian Arctic
Source: Sci Rep. 2022 Nov 30;12:20630. doi: 10.1038/s41598-022-24989-7 (PMC9712395; doi:10.1038/s41598-022-24989-7)
Supplement: Supplementary file 6 — Supplementary Information 6. [file 41598_2022_24989_MOESM6_ESM.pdf]

Dataset S4. The COI sequence-based identification of *Theromyzon tessulatum* (O. F. Müller, 1773) using the Barcoding of Life Database (BOLD IDS)

BOLD TaxonID Tree

```
Title            : COI FULL DATABASE includes records without species designati...
Date             : 3-October-2022
Data Type        : Nucleotide
Distance Model   : Kimura 2 Parameter
Marker           : COI-5P
Codon Positions  : 1st, 2nd, 3rd
Labels           : Extra Info, Country & Province, Family
Filters          : Length > 200
Attachment       : Photographs & Spreadsheet

Sequence Count   : 101
Species count    : 13
Genus count      : 5
Family count     : 1
Unidentified     : 36
```

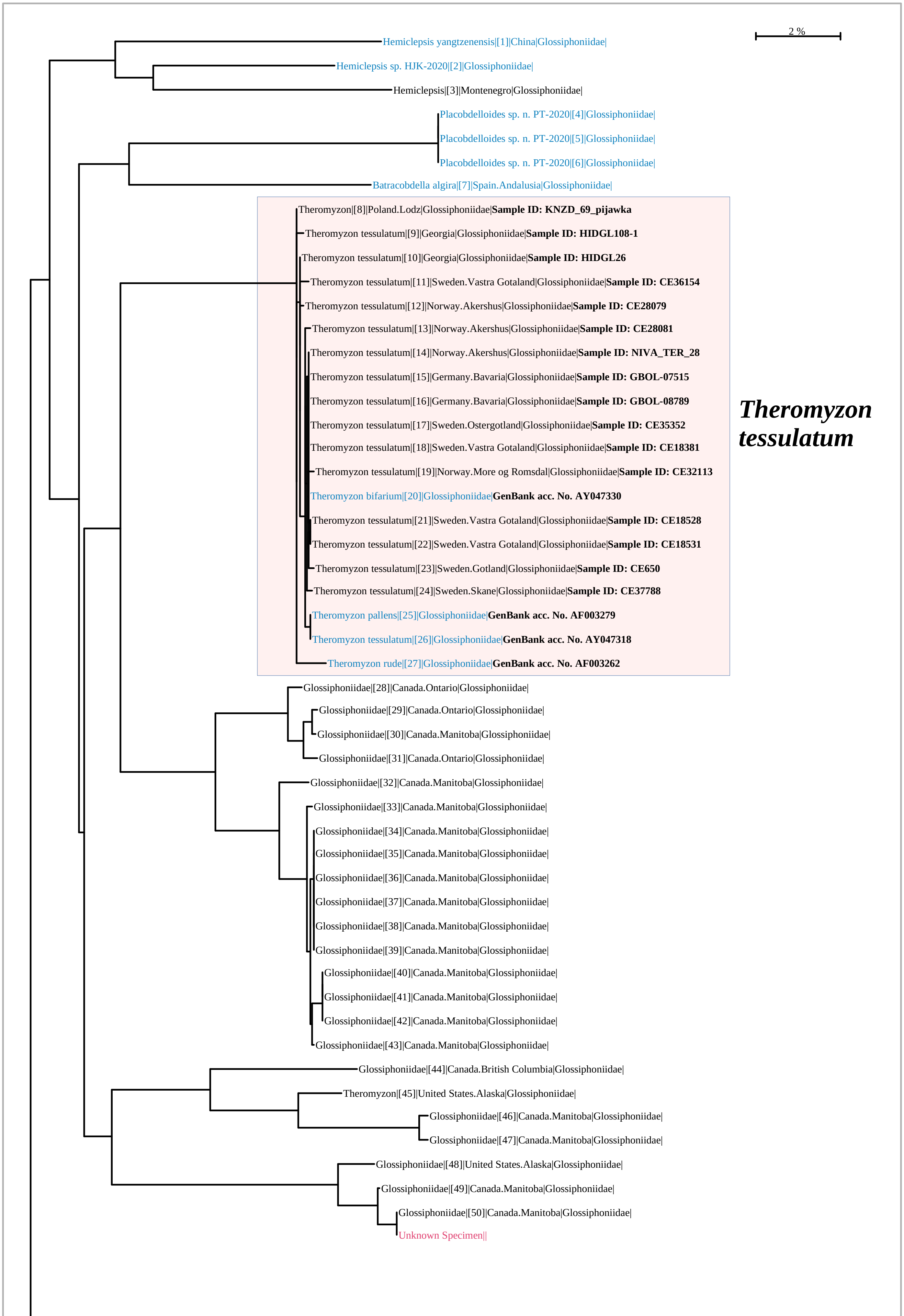
2 %
Hemiclepsis yangtzenensis|[1]|China|Glossiphoniidae|
Hemiclepsis sp. HJK-2020|[2]|Glossiphoniidae|
Hemiclepsis|[3]|Montenegro|Glossiphoniidae|
Placobdelloides sp. n. PT-2020|[4]|Glossiphoniidae|
Placobdelloides sp. n. PT-2020|[5]|Glossiphoniidae|
Placobdelloides sp. n. PT-2020|[6]|Glossiphoniidae|
Batracobdella algira|[7]|Spain.Andalusia|Glossiphoniidae|
Theromyzon|[8]|Poland.Lodz|Glossiphoniidae|Sample ID: KNZD_69_pijawka
Theromyzon tessulatum|[9]|Georgia|Glossiphoniidae|Sample ID: HIDGL108-1
Theromyzon tessulatum|[10]|Georgia|Glossiphoniidae|Sample ID: HIDGL26
Theromyzon tessulatum|[11]|Sweden.Vastra Gotaland|Glossiphoniidae|Sample ID: CE36154
Theromyzon tessulatum|[12]|Norway.Akershus|Glossiphoniidae|Sample ID: CE28079
Theromyzon tessulatum|[13]|Norway.Akershus|Glossiphoniidae|Sample ID: CE28081
Theromyzon tessulatum|[14]|Norway.Akershus|Glossiphoniidae|Sample ID: NIVA_TER_28
Theromyzon tessulatum|[15]|Germany.Bavaria|Glossiphoniidae|Sample ID: GBOL-07515
Theromyzon tessulatum|[16]|Germany.Bavaria|Glossiphoniidae|Sample ID: GBOL-08789
Theromyzon tessulatum|[17]|Sweden.Ostergotland|Glossiphoniidae|Sample ID: CE35352
Theromyzon tessulatum|[18]|Sweden.Vastra Gotaland|Glossiphoniidae|Sample ID: CE18381
Theromyzon tessulatum|[19]|Norway.More og Romsdal|Glossiphoniidae|Sample ID: CE32113
Theromyzon bifarium|[20]|Glossiphoniidae|GenBank acc. No. AY047330
Theromyzon tessulatum|[21]|Sweden.Vastra Gotaland|Glossiphoniidae|Sample ID: CE18528
Theromyzon tessulatum|[22]|Sweden.Vastra Gotaland|Glossiphoniidae|Sample ID: CE18531
Theromyzon tessulatum|[23]|Sweden.Gotland|Glossiphoniidae|Sample ID: CE650
Theromyzon tessulatum|[24]|Sweden.Skane|Glossiphoniidae|Sample ID: CE37788
Theromyzon pallens|[25]|Glossiphoniidae|GenBank acc. No. AF003279
Theromyzon tessulatum|[26]|Glossiphoniidae|GenBank acc. No. AY047318
Theromyzon rude|[27]|Glossiphoniidae|GenBank acc. No. AF003262
Theromyzon tessulatum
Glossiphoniidae|[28]|Canada.Ontario|Glossiphoniidae|
Glossiphoniidae|[29]|Canada.Ontario|Glossiphoniidae|
Glossiphoniidae|[30]|Canada.Manitoba|Glossiphoniidae|
Glossiphoniidae|[31]|Canada.Ontario|Glossiphoniidae|
Glossiphoniidae|[32]|Canada.Manitoba|Glossiphoniidae|
Glossiphoniidae|[33]|Canada.Manitoba|Glossiphoniidae|
Glossiphoniidae|[34]|Canada.Manitoba|Glossiphoniidae|
Glossiphoniidae|[35]|Canada.Manitoba|Glossiphoniidae|
Glossiphoniidae|[36]|Canada.Manitoba|Glossiphoniidae|
Glossiphoniidae|[37]|Canada.Manitoba|Glossiphoniidae|
Glossiphoniidae|[38]|Canada.Manitoba|Glossiphoniidae|
Glossiphoniidae|[39]|Canada.Manitoba|Glossiphoniidae|
Glossiphoniidae|[40]|Canada.Manitoba|Glossiphoniidae|
Glossiphoniidae|[41]|Canada.Manitoba|Glossiphoniidae|
Glossiphoniidae|[42]|Canada.Manitoba|Glossiphoniidae|
Glossiphoniidae|[43]|Canada.Manitoba|Glossiphoniidae|
Glossiphoniidae|[44]|Canada.British Columbia|Glossiphoniidae|
Theromyzon|[45]|United States.Alaska|Glossiphoniidae|
Glossiphoniidae|[46]|Canada.Manitoba|Glossiphoniidae|
Glossiphoniidae|[47]|Canada.Manitoba|Glossiphoniidae|
Glossiphoniidae|[48]|United States.Alaska|Glossiphoniidae|
Glossiphoniidae|[49]|Canada.Manitoba|Glossiphoniidae|
Glossiphoniidae|[50]|Canada.Manitoba|Glossiphoniidae|
Unknown Specimen||

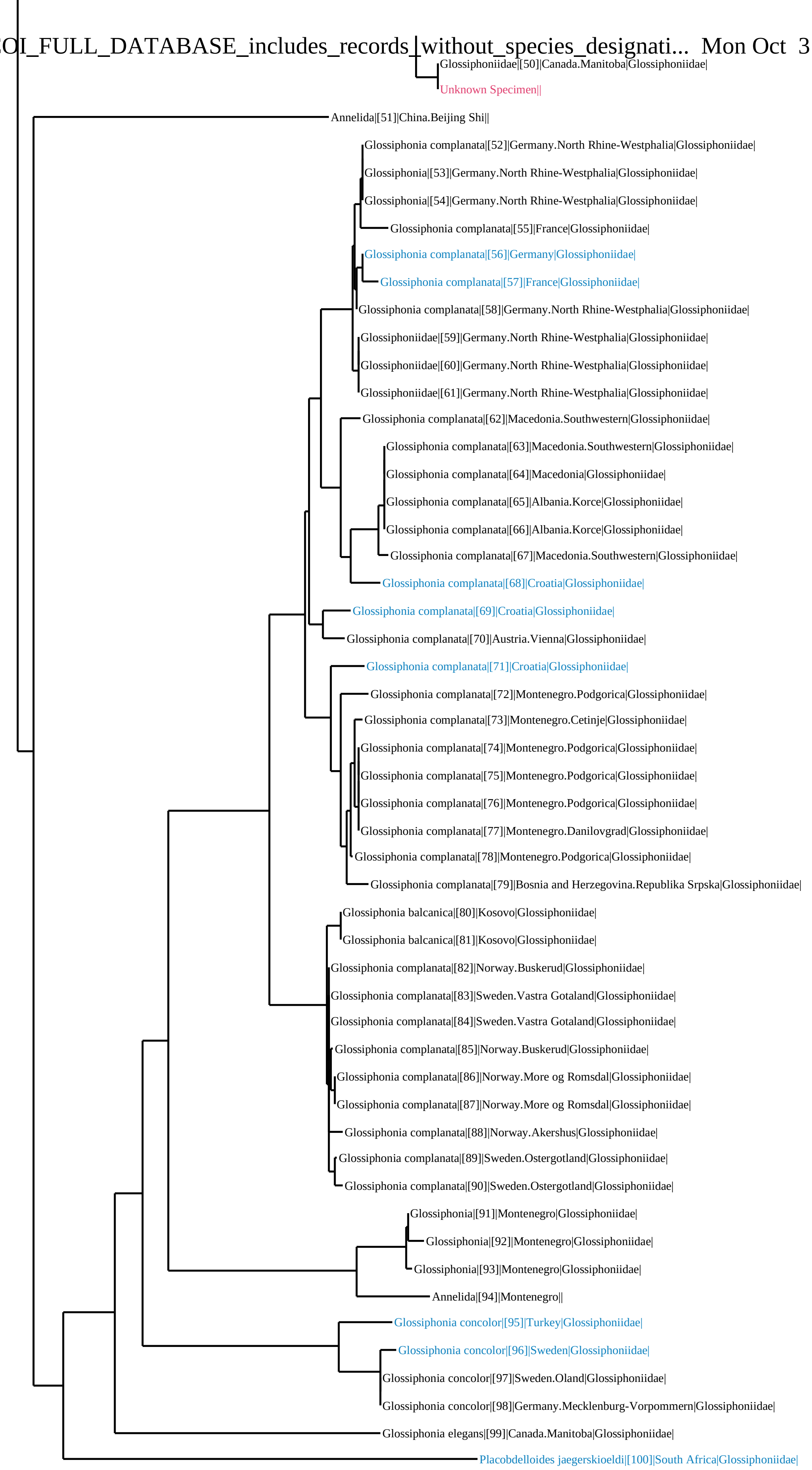
Glossiphoniidae|[50]|Canada.Manitoba|Glossiphoniidae|
Unknown Specimen||
Annelida|[51]|China.Beijing Shi||
Glossiphonia complanata|[52]|Germany.North Rhine-Westphalia|Glossiphoniidae|
Glossiphonia|[53]|Germany.North Rhine-Westphalia|Glossiphoniidae|
Glossiphonia|[54]|Germany.North Rhine-Westphalia|Glossiphoniidae|
Glossiphonia complanata|[55]|France|Glossiphoniidae|
Glossiphonia complanata|[56]|Germany|Glossiphoniidae|
Glossiphonia complanata|[57]|France|Glossiphoniidae|
Glossiphonia complanata|[58]|Germany.North Rhine-Westphalia|Glossiphoniidae|
Glossiphoniidae|[59]|Germany.North Rhine-Westphalia|Glossiphoniidae|
Glossiphoniidae|[60]|Germany.North Rhine-Westphalia|Glossiphoniidae|
Glossiphoniidae|[61]|Germany.North Rhine-Westphalia|Glossiphoniidae|
Glossiphonia complanata|[62]|Macedonia.Southwestern|Glossiphoniidae|
Glossiphonia complanata|[63]|Macedonia.Southwestern|Glossiphoniidae|
Glossiphonia complanata|[64]|Macedonia|Glossiphoniidae|
Glossiphonia complanata|[65]|Albania.Korce|Glossiphoniidae|
Glossiphonia complanata|[66]|Albania.Korce|Glossiphoniidae|
Glossiphonia complanata|[67]|Macedonia.Southwestern|Glossiphoniidae|
Glossiphonia complanata|[68]|Croatia|Glossiphoniidae|
Glossiphonia complanata|[69]|Croatia|Glossiphoniidae|
Glossiphonia complanata|[70]|Austria.Vienna|Glossiphoniidae|
Glossiphonia complanata|[71]|Croatia|Glossiphoniidae|
Glossiphonia complanata|[72]|Montenegro.Podgorica|Glossiphoniidae|
Glossiphonia complanata|[73]|Montenegro.Cetinje|Glossiphoniidae|
Glossiphonia complanata|[74]|Montenegro.Podgorica|Glossiphoniidae|
Glossiphonia complanata|[75]|Montenegro.Podgorica|Glossiphoniidae|
Glossiphonia complanata|[76]|Montenegro.Podgorica|Glossiphoniidae|
Glossiphonia complanata|[77]|Montenegro.Danilovgrad|Glossiphoniidae|
Glossiphonia complanata|[78]|Montenegro.Podgorica|Glossiphoniidae|
Glossiphonia complanata|[79]|Bosnia and Herzegovina.Republika Srpska|Glossiphoniidae|
Glossiphonia balcanica|[80]|Kosovo|Glossiphoniidae|
Glossiphonia balcanica|[81]|Kosovo|Glossiphoniidae|
Glossiphonia complanata|[82]|Norway.Buskerud|Glossiphoniidae|
Glossiphonia complanata|[83]|Sweden.Vastra Gotaland|Glossiphoniidae|
Glossiphonia complanata|[84]|Sweden.Vastra Gotaland|Glossiphoniidae|
Glossiphonia complanata|[85]|Norway.Buskerud|Glossiphoniidae|
Glossiphonia complanata|[86]|Norway.More og Romsdal|Glossiphoniidae|
Glossiphonia complanata|[87]|Norway.More og Romsdal|Glossiphoniidae|
Glossiphonia complanata|[88]|Norway.Akershus|Glossiphoniidae|
Glossiphonia complanata|[89]|Sweden.Ostergotland|Glossiphoniidae|
Glossiphonia complanata|[90]|Sweden.Ostergotland|Glossiphoniidae|
Glossiphonia|[91]|Montenegro|Glossiphoniidae|
Glossiphonia|[92]|Montenegro|Glossiphoniidae|
Glossiphonia|[93]|Montenegro|Glossiphoniidae|
Annelida|[94]|Montenegro||
Glossiphonia concolor|[95]|Turkey|Glossiphoniidae|
Glossiphonia concolor|[96]|Sweden|Glossiphoniidae|
Glossiphonia concolor|[97]|Sweden.Oland|Glossiphoniidae|
Glossiphonia concolor|[98]|Germany.Mecklenburg-Vorpommern|Glossiphoniidae|
Glossiphonia elegans|[99]|Canada.Manitoba|Glossiphoniidae|
Placobdelloides jaegerskioeldi|[100]|South Africa|Glossiphoniidae|
